# Dosing Strategies for High-Alert Medications in Obese Pediatric Patients: A Systematic Review

**DOI:** 10.3390/ph19050766

**Published:** 2026-05-13

**Authors:** Yolanda Hernández-Gago, Pedro J. Alcalá Minagorre, José Germán Sánchez-Hernández, Belén Rodríguez Marrodán, Laura Hernández Sabater, Ana Cristina Rodríguez Negrín, Claudio-Alberto Rodríguez-Suárez

**Affiliations:** 1 Department of Hospital Pharmacy, Insular Maternal and Child University Hospital Complex, 35016 Las Palmas de Gran Canaria, Spain; arodnegz@gobiernodecanarias.org; 2 Department of Pediatric, Dr. Balmis University General Hospital, Alicante Institute for Health and Biomedical Research (ISABIAL), 03010 Alicante, Spain; alcala_ped@gva.es; 3 Department of Hospital Pharmacy, University Hospital of Salamanca, 37007 Salamanca, Spain; jgermansanchez@saludcastillayleon.es; 4 Department of Hospital Pharmacy, Puerta de Hierro University Hospital, 28222 Majadahonda, Spain; brmarrodan@salud.madrid.org; 5 Department of Pediatric, Northwest Regional Hospital, 30400 Caravaca de la Cruz, Spain; laura.hernandez9@carm.es; 6 Research Support Department, Insular Maternal and Child University Hospital Complex, 35016 Las Palmas de Gran Canaria, Spain; claudioalberto.rodriguez@ulpgc.es; 7 Nursing Department, Faculty of Healthcare Science, University of Las Palmas de Gran Canaria (ULPGC), 35016 Las Palmas de Gran Canaria, Spain

**Keywords:** pediatric obesity, high-alert medications, pharmacokinetics, dose adjustment, body size descriptors, drug safety, therapeutic drug monitoring

## Abstract

**Background/Objective**: Childhood obesity induces physiological changes that alter drug distribution and clearance; however, these patients are often excluded from clinical trials, creating a critical safety gap for high-alert medications (HAM). The Objective was to evaluate HAM dosing strategies and pharmacokinetic (PK) alterations in overweight and obese pediatric patients. **Methods**: A systematic review was conducted and registered in PROSPERO (CRD42023452126). A search of MEDLINE, EMBASE, Web of Science, and Cochrane CENTRAL (1990–March 2026) identified studies reporting dosing strategies or PK of HAM in obese or overweight pediatric patients. Studies were included if they reported dosing recommendations or PK parameters. Eligible designs comprised prospective and retrospective, randomized and non-randomized, observational (cohort, case-control, and cross-sectional), case series, case reports, and narrative and systematic reviews. Study selection, data extraction, and quality assessment were conducted independently by two reviewers. Methodological quality was assessed using validated tools, and results were synthesized qualitatively. **Results**: Of 5801 records, 91 studies were included, providing evidence for only 27% of the evaluated HAM. Total body weight (TBW) appeared to be appropriate for insulin and vancomycin, although close monitoring was required. TBW-based dosing was associated with approximately 20% overexposure for enoxaparin, supporting the use of fat-free mass (FFM) or reduced dosing strategies. Increased clearance may justify higher doses for amlodipine and consideration of adult-equivalent dosing for metformin in adolescents. For gentamicin, FFM appeared to be the most appropriate descriptor, while adjusted body weight was used for valproic acid. In anesthetics and sedatives, reduced TBW-based dosing may be considered for propofol, whereas ideal body weight (IBW) or FFM were generally preferred for ketamine and dexmedetomidine. Analgesics such as fentanyl and morphine may require IBW- or FFM-based dosing, and maintenance dosing of paracetamol may require adjustment. **Conclusions**: Evidence remains limited and heterogeneous, with no standardized dosing approach. Model-informed strategies—such as population PK (PopPK) and physiologically based PK model (PBPK) approaches—may be useful for hypothesis generation and exploring PK variability; however, their clinical applicability is constrained by the limited and heterogeneous evidence base, and they should be considered exploratory.

## 1. Introduction

Childhood and adolescent obesity has reached epidemic proportions and is currently recognized as one of the most serious public health challenges of the 21st century [1]. According to data from the World Health Organization (WHO), in 2022 more than 390 million children aged 5 to 19 years were overweight, of whom 160 million were already living with obesity; additionally, in 2024 more than 35 million children under 5 years of age were overweight [2]. If current trends continue, it is estimated that by 2050 the number of children and adolescents with obesity will reach 360 million worldwide [3].

This increase is directly associated with a higher risk of developing complex chronic diseases at earlier ages, including type 2 diabetes mellitus (T2DM), dyslipidemia, non-alcoholic fatty liver disease (NAFLD), arterial hypertension, and polycystic ovary syndrome [4,5]. Consequently, this population generates an increased healthcare demand and a significantly greater use of pharmacotherapeutic resources [5,6].

From a pharmacological perspective, obesity is associated with important physiological alterations, an increased fat mass-to-lean mass ratio, expanded blood volume and cardiac output, reduced tissue perfusion, and altered hepatic and renal function. These changes can significantly modify pharmacokinetic (PK) parameters such as, bioavailability (F), volume of distribution (Vd), and total body clearance (Cl). As a result, drug disposition and therapeutic response may differ substantially in obese pediatric patients compared with their normal-weight counterparts [7,8,9].

Despite the clinical relevance of these alterations, there is still limited evidence regarding the impact of obesity on the pharmacokinetics and pharmacodynamics (PK/PD) of commonly used drugs in pediatric populations [10]. This knowledge gap is due to the limited participation of children with obesity in clinical trials [11]. In daily practice, drug dosing is typically calculated according to total body weight (TBW), assuming a normal body composition. However, this approach may lead to overdosing—sometimes exceeding adult doses—whereas the use of ideal body weight (IBW) or adjusted body weight (AjBW) may result in subtherapeutic exposure [7,12].

### Mechanistic Framework for Selecting Body Size Descriptors

To address the lack of a unifying rationale in the selection of body size descriptors, we propose a structured and mechanistic decision framework that integrates PK principles with the pathophysiology of pediatric obesity. This framework is based on the premise that no universal rule can be applied, as descriptor selection depends on the physicochemical properties of the drug, age-dependent maturation, and the specific therapeutic objective. The first step is to distinguish the dosing scenario, since loading and maintenance doses rely on different biological parameters. For loading doses, which are primarily determined by the Vd to achieve target concentrations rapidly, descriptor selection depends on the drug’s solubility and distribution pattern. For hydrophilic drugs that distribute mainly into extracellular water and lean tissue, IBW or fat-free mass (FFM) is recommended to avoid overdosing. In contrast, lipophilic drugs with affinity for adipose tissue may require TBW or AjBW, although this approach should be applied with caution due to variability in adipose distribution. A Vd threshold of 5 L/kg has been proposed to distinguish these patterns, with higher values generally associated with lipophilicity and TBW-based dosing; however, relevant exceptions exist, such as digoxin, for which IBW is preferred despite a high Vd due to its limited affinity for adipose tissue. Additionally, plasma protein binding should be considered, as alterations in protein levels (e.g., albumin or α1-acid glycoprotein) may modify the unbound fraction of the drug, thereby influencing distribution, particularly for highly protein-bound compounds. For maintenance dosing, which depends on Cl, the use of TBW may lead to overestimation, as elimination capacity is more closely related to lean tissues. Therefore, lean-based descriptors such as IBW, FFM, or lean body weight (LBW) are more appropriate predictors of Cl. In addition, allometric scaling should be applied, using an exponent of 0.75 for Cl, to account for the non-linear relationship between body size and drug elimination. As a unifying concept, normal fat mass (NFM) incorporates both lean mass and a drug-specific fraction of fat mass and may be applicable across different age groups and body compositions. All this information should be integrated with the assessment of renal and hepatic function, as obesity may be associated with glomerular hyperfiltration, as well as NAFLD and alterations in hepatic blood flow and enzyme activity that can modify drug elimination independently of body size. Finally, this framework should incorporate safety considerations, including not exceeding the maximum recommended adult dose and the use of therapeutic drug monitoring (TDM) for drugs with a narrow therapeutic index to support individualized dose adjustment [7,13,14,15].

The lack of standardized dosing adjustment strategies contributes to frequent dosing errors. A systematic review has estimated that up to two-thirds of drugs prescribed in children with obesity result in infra- or supratherapeutic concentrations, thereby increasing the risk of treatment failure or toxicity [9,10]. This issue may be particularly critical for so-called high-alert medications (HAM), defined as drugs that, when used incorrectly, have a higher likelihood of causing serious or even fatal harm to patients [16].

Therefore, the aim of this systematic review was to evaluate the current evidence on dosing strategies for HAM in overweight and obese pediatric patients, with the objective of identifying situations in which dose adjustment is required and determining the most appropriate approaches across different pediatric age groups. In addition, this review aims to analyze obesity-related changes in key PK parameters—including Vd, half-life (t_1/2_), area under the plasma concentration–time curve (AUC), and Cl—and their potential impact on dosing strategies in this population.

## 2. Materials and Methods

A systematic review on pharmacological dosing in pediatric populations with obesity or overweight was conducted in accordance with the Preferred Reporting Items for Systematic Reviews and Meta-Analyses (PRISMA) guidelines, and a PRISMA checklist was completed (Supplementary File S1). The review protocol was prospectively registered in the PROSPERO database (registration number: CRD42023452126; available at: https://www.crd.york.ac.uk/PROSPERO/view/CRD42023452126, accessed on 17 August 2023). No deviations from the registered protocol were made.

### 2.1. Identification of High-Alert Medications

The identification of HAM in children older than 2 years was based on a previous study using a two-round Delphi consensus methodology. This structured consensus process involved an interdisciplinary panel of pediatricians and hospital pharmacists, in which 100 HAM and 24 pharmacological groups were identified. The complete list of included drugs is presented in Table 1 [17].

### 2.2. Search Strategy

A comprehensive literature search was conducted in the following electronic databases: Cochrane CENTRAL, Web of Science (WOS), MEDLINE Ovid SP, and EMBASE Ovid SP. The aim was to identify all available evidence on pharmacological dosing in pediatric populations with obesity or overweight. No language restrictions were applied, and studies published between 1990 and March 2026 were considered.

The search strategy was structured into three conceptual components: (i) target population (children and adolescents aged 0–18 years with obesity or overweight), (ii) intervention/exposure (HAM and their pharmacological classes, including administration and dosage terms), and (iii) clinical context (critical care and pediatric intensive care unit (PICU) settings).

The search strategy included the following combination of controlled vocabulary and entry terms: (obesity OR “morbid obesity” OR overweight) AND (pharmacokinetics OR “drug dose” OR “dose calculation algorithm” OR “dose-response”) AND (infant OR child OR preschool OR school OR adolescent) AND (the specific drugs included in this review). The full electronic search strategy for each database is provided in Supplementary File S2.

To ensure completeness, the search strategy was updated prior to final publication (March 2026). Newly identified records were screened for inclusion and were reflected in the updated PRISMA flow diagram.

### 2.3. Eligibility Criteria and Study Selection

Inclusion and exclusion criteria were predefined prior to study selection in accordance with PRISMA guidelines.

Studies were included if they reported dosing recommendations in obese and/or overweight pediatric patients for the selected HAM, as well as those evaluating PK parameters such as Vd, Cl, and t_1/2_, AUC, and/or plasma concentrations in this population. Eligible study designs included prospective and retrospective studies, randomized and non-randomized comparative studies, observational studies (cohort, case/control, and cross-sectional), case series and case reports, and narrative and systematic reviews.

Studies were excluded if they did not report PK data or dosing strategies in obese pediatric patients for HAM. Excluded designs included editorials, letters to the editor, conference abstracts and studies based on animal models.

### 2.4. Study Selection Process

All retrieved records were exported in RIS or TXT format and imported into Rayyan platform [18] for screening. Duplicate records were removed through automatic detection followed by manual verification by checking title, journal, author, and year. Two reviewers independently screened titles/abstracts and full texts. Discrepances were resolved by a third reviewer after full-text assessment. A standard operating procedure for the study selection provided in the Supplementary File S3.

### 2.5. Data Extraction

Data extraction was performed independently by two reviewers using a standardized data collection form in Microsoft Excel (Microsoft Corporation, Redmond, WA, USA, Version 2604). Discrepancies were resolved by reviewers consensus.

The following variables were extracted:

General study characteristics: number of patients, therapeutic group and drug, study identifier, inclusion or exclusion status, and reasons for exclusion, study title, DOI, journal and first author.

Study design and population: study design and type, clinical condition, age range, definition of obesity, and description of obese and normal-weight groups.

Outcomes: The primary outcomes included PK parameters (Vd, Cl, t_1/2_, AUC), and dosing strategy (TBW, IBW, AjBW, NFM, FFM). Secondary outcomes included clinical outcomes (efficacy, therapeutic failure, adverse effects).

Additional variables: primary and secondary objectives, conclusions and relevant observations.

For each drug, additional PK data were obtained from tertiary sources, including Lexicomp^®^ online drug information database (Wolters Kluwer Health, Riverwoods, IL, USA) and the IBM Micromedex^®^ electronic database (IBM Watson Health, Greenwood Village, CO, USA). Unless otherwise specified, reported values correspond to adult populations and are presented in the original units [19,20].

Given the heterogeneity in study designs, patient populations, pharmacological agents, and reported outcomes, a quantitative synthesis (meta-analysis) was not feasible, and the results were synthesized narratively.

### 2.6. Quality Assessment and Risk of Bias

The methodological quality was assessed using validated tools according to study designs: Joanna Briggs Institute (JBI) critical appraisal tools [21] for observational (retrospective and prospective cohorts, case-control, and cross-sectional) and quasi-experimental studies. Scale for the Assessment of Narrative Review Articles (SANRA) for narrative reviews [22]. European Medicines Agency (EMA) guidelines for PK modeling studies (population PK and physiologically based pharmacokinetic model (PBPK) [23]. These tools were selected based on their suitability for the included study designs.

Assessments were performed independently by multiple reviewers. Disagreements were resolved by consultation with an additional reviewer. Studies were categorized according to methodological quality based on the proportion of positive responses in the corresponding checklists: <60% low quality; 61–80% moderate quality; and >81% high quality. These quality ratings were considered during data synthesis to prioritize higher-quality evidence.

Publication bias was not formally assessed due to the heterogeneity and predominance of non-comparative studies.

### 2.7. Evidence Synthesis and Classification

In addition to the assessment of methodological quality, a structured classification of the included studies was performed to support the synthesis of evidence.

The type of evidence was categorized according to study design as observational, experimental, model-informed (including PopPK and PBPK studies), or review-based.

The amount of evidence for each PK group was qualitatively assessed based on the number of available studies and categorized as very low, low, moderate, or moderate–high.

Consistency of evidence was defined according to the degree of agreement between study findings and categorized as consistent (concordant results across multiple studies), limited (few studies or methodological constraints), or conflicting (inconsistent findings across studies).

## 3. Results

### 3.1. Literature Search and Study Identification

**Figure 1:**
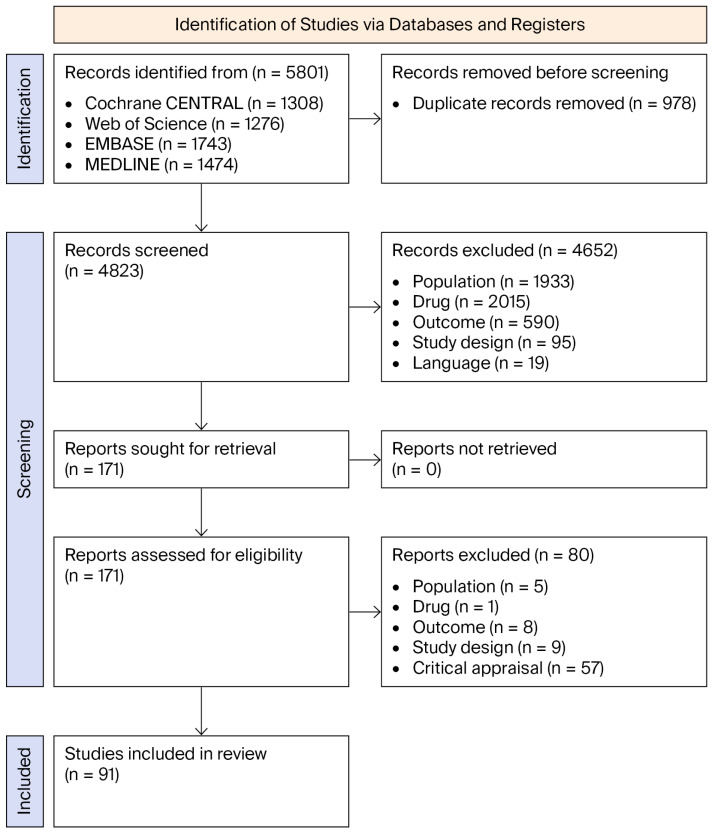
The flowchart of the selection process of literature and reports based on PRISMA.

A total of 5801 records were identified through database searches. After removal of duplicates, 4823 records were screened by title and abstract, leading to the exclusion of 4652 records. A total of 171 full-text articles were assessed for eligibility, of which 80 were excluded for predefined reasons, primarily due to critical appraisal concerns. Ultimately, 91 studies were included in the review. The complete study selection process is detailed in the PRISMA flowchart (Figure 1).

The studies identified regarding dosing or PK in pediatric patients with excess body weight corresponded to 27 of the 100 HAM identified, with the number of studies per drug ranging from 1 to 12. Table 1 details the number of studies identified per drug and pharmacological group for all included HAM.

Where appropriate, the interpretation of dosing strategies was supported by PK considerations, including drug distribution, clearance mechanisms, and physicochemical properties.

### 3.2. Methodological Quality of Included Studies 

Of the 91 studies included in the systematic review, 38 (41.76%) were observational, 21 (23.08%) were experimental and/or quasi-experimental, 20 (21.98%) were PopPK or PBPK studies, and 12 were reviews or meta-analyses (13.18%). Overall, 69.05% of the studies included fewer than 30 patients in the obese group, 44.05% reported PK data, and 59.52% explicitly specified the dosing approach in obese patients. Among the 38 observa-tional studies, 78.95% were retrospective.

Table 2 classifies the studies according to their scientific quality (low, moderate, or high) based on the JBI checklists for observational and experimental studies, SANRA for reviews, and the European Medicines Agency guidelines for PopPK/PBPK studies.

### 3.3. Structured Synthesis of Evidence by Pharmacological Class and Pharmacokinetic Behavior

To enhance interpretability and clinical applicability, the findings were synthesized according to pharmacological class and key PK characteristics, including distribution properties and clearance mechanisms. Pharmacological groupings were adapted from the original classification presented in Table 1 and reorganized according to PK properties and clinical relevance. Specifically, some groups were subdivided (e.g., antibiotics into aminoglycosides and glycopeptides) or combined (e.g., low-molecular-weight heparins and other antithrombotic agents) to facilitate interpretation of dosing strategies. This approach was necessary due to the heterogeneity of PK profiles within certain therapeutic classes and to better reflect drug-specific characteristics such as lipophilicity, distribution, and clearance.

For hydrophilic drugs with limited distribution into adipose tissue (e.g., aminoglycosides and heparins), evidence was consistent, suggesting the use of lean-based descriptors such as FFM or AjBW. TBW-based dosing in these agents was frequently associated with increased exposure, reflecting limited adipose distribution and clearance driven by lean tissues.

For drugs with broader distribution and predominant renal elimination (e.g., vancomycin), evidence was moderate but generally consistent, suggesting that TBW correlates with absolute PK parameters, although weight-normalized values may be reduced or similar in obesity. In these cases, TBW-based dosing combined with TDM, preferably guided by AUC/MIC, may be appropriate.

For drugs with mixed PK profiles (e.g., insulin and metformin), evidence was limited and heterogeneous. Insulin requirements generally correlated with TBW, whereas IBW-based approaches tended to underestimate dosing. In contrast, metformin showed increased clearance in obese adolescents, supporting the potential use of adult-equivalent doses in selected cases.

For highly lipophilic drugs (e.g., anesthetics, sedatives, and opioids such as propofol, ketamine, and fentanyl), the available evidence remained limited and, in some cases, conflicting. Although lipophilicity would theoretically support TBW-based dosing due to increased distribution into adipose tissue, clinical data suggested a more cautious approach. In several cases, IBW or FFM-based dosing was preferred to mitigate the risk of drug accumulation and delayed clearance, particularly during prolonged administration.

Finally, for drugs with high protein binding and hepatic metabolism (e.g., warfarin), evidence was limited, suggesting that obesity-related changes in volume of distribution and metabolic activity may alter dose requirements, although findings remain inconsistent, reinforcing the need for individualized approaches and careful monitoring.

Overall, the available evidence remains uneven across pharmacological classes, with a predominance of small observational studies and model-informed approaches, limiting the ability to establish standardized dosing recommendations. A structured summary of the type, amount, methodological quality, and consistency of evidence across pharmacological groups is presented in Table 3 to facilitate interpretation of these findings.

#### 3.3.1. Parenteral Insulin

Subcutaneous regular human insulin is well absorbed, does not bind to plasma proteins, and is distributed in a relatively low Vd of 0.15 L/kg. Elimination is rapid through enzymatic degradation in the liver, kidney, and adipose tissue/muscle, with a short half-life that varies depending on the formulation (approximately 3.3 h for subcutaneous administration (SC) and 5–15 min for intravenous administration (IV)).

Three articles evaluating parenteral antidiabetic agents in pediatric patients with excess body weight were included in the review, as detailed in Table 4. All three studies are based on empirical PK data derived from observational and clinical settings. Chalk et al. [24] conducted a retrospective cohort study in children and adolescents with newly diagnosed type 1 diabetes mellitus (T1DM), assessing the impact of the weight descriptor on insulin glargine dosing. No statistically significant differences were observed in the initial dose based on TBW (approximately 0.3 U/kg/day), fasting glucose levels, or dose adjustments among underweight, normal-weight, and overweight/obese patients. During follow-up, TBW was identified as the most accurate predictor for dose adjustment in overweight/obese patients, whereas the use of IBW, regardless of the calculation method, underestimated insulin requirements.

Flokas et al. [25] conducted a cross-sectional observational study in a large pediatric cohort with T1DM, evaluating the relationship between body mass index and insulin requirements in patients with optimal glycemic control. In this subgroup, no statistically significant differences were observed in the total daily insulin dose AjBW between obese and normal-weight patients, indicating that children with obesity do not require higher doses per kilogram to achieve adequate glycemic control.

Palomo Atance et al. [26] conducted a cross-sectional observational study in children with T1DM, evaluating the influence of body weight on insulin dosing using different body size descriptors. No statistically significant differences were observed in insulin dose when expressed as units per kilogram of TBW across weight groups. However, when the dose was adjusted by body surface area (BSA) (U/m^2^/day), overweight and obese patients showed significantly higher requirements, indicating that this descriptor is more sensitive for detecting the impact of obesity. Additionally, obese children older than 11 years exhibited a lower estimated rate of glucose disposal, consistent with increased insulin resistance.

#### 3.3.2. Other Antidiabetic Agents

Studies with semaglutide, liraglutide, and metformin were identified, as shown in Table 4.

Semaglutide

Semaglutide is an antidiabetic agent that has already been approved by the FDA for the treatment of patients older than 12 years with obesity or overweight associated with at least one other weight-related comorbidity, at an initial SC dose of 0.25 mg and a maintenance dose of 2.4 mg weekly.

Semaglutide has a high SC F of 89%, extensive plasma protein binding (~99%), a moderate Vd of 12.5 L for the SC route, is metabolized mainly by proteolysis and beta-oxidation, and has a long half-life of approximately 1 week. Pediatric PK show similar exposure, with a slightly shorter tmax and comparable AUC and Cmax values to adults at equivalent doses.

Two studies were identified, van Boxel et al. [27] conducted a retrospective observational study in 50 children aged 10–18 years with obesity treated with once-weekly SC semaglutide titrated to 1 mg. At 6 months, BMI SDS decreased by −0.32 ± 0.27 and body weight by −7.03 ± 7.50 kg (6.4% weight loss), with greater reductions at 12 months (BMI SDS −0.54 ± 0.52; −9.7 kg; 8.9% weight loss). The authors concluded that semaglutide is a safe and effective adjunct for weight reduction in children with obesity. The other one of which [28] developed PBPK models of semaglutide in pediatric patients (10–17 years) to characterize PK and inform safe dosing strategies. Adult data were extrapolated to children, incorporating age and body weight differences. PK simulations showed higher Cmax values in younger children (10–14 years) with normal weight compared to adults, indicating an increased risk of dose-dependent gastrointestinal adverse events (AEs). An inverse relationship between body weight and Cmax was observed. Clinically, fixed dosing strategies may lead to overexposure in lighter patients. The authors concluded that age- and weight-adjusted dosing is necessary to optimize safety and efficacy in pediatric diabetes treatment, although direct clinical validation remains limited.

Overall, available evidence is limited and should be interpreted with caution. In semaglutide, pediatric obesity appears to influence initial distribution (Cmax, Tmax) more than Cl in a proportional manner. Body weight mainly affects exposure but does not support linear TBW-based dose escalation, favoring fixed or cautiously weight-/age-adjusted dosing to minimize overexposure in lower-weight patients.

Liraglutide

Liraglutide is a SC antidiabetic agent approved by the FDA for the treatment of patients older than 12 years with obesity or overweight associated with at least one other weight-related comorbidity and for patients older than 10 years with T2DM. It is administered at an initial dose of 0.6 mg SC and a maintenance dose of 3 mg weekly for obesity and 1.8 mg weekly for T2DM. Liraglutide shows consistent PK across adult and pediatric populations, with slightly higher Cl and exposure in pediatric patients aged 10 to 17 years. The drug has moderate F (55%), high plasma protein binding (>98%), a moderate Vd (0.07 L/kg IV), is metabolized by general protein catabolism without a major organ route and has a t_1/2_ of approximately 13 h.

Six studies [29,30,31,32,33,34] evaluating PK in obese pediatric patients were identified, four of which included patients younger than 12 years. Only one provided conclusive data: a prospective, double-blind experimental study including 24 patients aged 7–11 years. Ascending SC doses of liraglutide ranging from 0.3 mg to 3 mg daily were administered according to tolerance. A lower apparent Cl and higher exposure were observed in this age group compared with adolescents and adults, which was attributed to their lower body weight rather than to significant differences in metabolism or elimination. Based on these results, dose adjustment should be based on body weight [30].

In line with these PK findings, Fox et al. [34] conducted a phase 3 randomized, double-blind, placebo-controlled trial to evaluate the efficacy and safety of liraglutide in children aged 6 to <12 years with obesity. At 56 weeks, liraglutide significantly reduced BMI and body weight, with 46% achieving ≥5% BMI reduction. Overall, and considering the limitations of the available evidence, current data suggest that pediatric obesity has a limited and non-proportional impact on Vd and Cl for liraglutide, with body weight acting primarily as a modulator of systemic exposure. This supports weight-based or stepwise dosing strategies, while avoiding the assumption of a linear relationship between TBW and PK parameters.

Metformin

Oral antidiabetic agent with an F of 50–60%, negligible plasma protein binding, and a large Vd of 596–654 L. It is eliminated renally without metabolism, with a t_1/2_ of 4.19–6.2 h. PK in the pediatric population aged 12–16 years does not show significant differences compared to adults.

A total of 12 studies evaluating the use of metformin in pediatric populations with overweight or obesity were identified. Only two reported PK results. One of them, conducted by van Rongen et al. [35], was a prospective uncontrolled study including 22 adolescent patients with overweight or obesity treated with metformin for 37 weeks. The authors observed that AUC decreased with increasing body weight and that apparent clearance (Cl/F) was similar to that observed in adults and higher than in non-obese children. They concluded that adult doses could be considered in obese adolescent patients if pediatric doses are therapeutically ineffective.

The second study, conducted by Ford et al. [36], consisted of PK modeling and simulations performed in adolescents without obesity, with overweight/obesity, and with severe obesity. As reported by van Rongen et al., a higher Cl/F was observed in adolescents with obesity compared to non-obese children and similar to that of adults. However, as these findings were derived from model-based analyses rather than direct clinical validation, their applicability to dosing recommendations should be interpreted cautiously.

Three systematic reviews [37,38,39] evaluated the use of metformin for weight reduction in children and adolescents with overweight/obesity and reported a modest reduction in BMI, which was not dose-dependent and not sustained over time.

Wang et al. [40] conducted a model-based meta-analysis including children and adolescents treated with metformin for BMI control in pediatric patients with obesity, T1DM, NAFLD, or precocious puberty. They observed a reduction in BMI that was not dose-dependent, with a slow onset and variable magnitude depending on the underlying pathophysiological condition.

Two studies evaluated the use of metformin in adolescents with overweight or obesity and diabetes. Libman et al. [41] conducted a prospective experimental placebo-controlled study to assess the effect of adding metformin to insulin therapy. At 26 weeks, a reduction in insulin requirements was observed, which was not associated with an improvement in hemoglobin A1c (HbA1c). In contrast, the systematic review by Liu et al. [39] found that metformin reduces overall HbA1c (0.37%) and insulin requirements by 0.11 U/kg/day in adolescents with T1DM. Glycemic improvement was significant only in the overall population (−0.53%), whereas weight loss (−1.93 kg) and BMI reduction were more pronounced in the obese subgroup.

Metformin is characterized by a large Vd and primarily reflects distribution into lean tissues rather than adipose tissue; therefore, in pediatric obesity, Vd does not increase proportionally with total body weight. In contrast, Cl, which is mainly renal, tends to be increased in obese adolescents, likely due to obesity-related changes such as enhanced glomerular filtration and renal size, resulting in reduced systemic exposure. This occurs with an increase in elimination that is not proportional to TBW but is sufficient to reduce drug exposure. These findings suggest that higher doses (similar to adult dosing) may be considered in adolescents with obesity when the clinical response is inadequate, while avoiding strictly linear TBW-based dose escalation and taking renal function into account to individualize therapy.

**Table 4:** Summary of studies describing dosing and PK and/or PD aspects of parenteral and oral antidiabetic agents.

	Study/Design	Patients/Sample Size	Methods	Results	Dosing Conclusions
Parenteral Antidiabetics (Insulin)	Chalk et al., 2019 [24] Observational, Retrospective cohort	0 years–adolescents (T1DM); n = 81; 24 overweight/obesity (BMI > P85), 57 normal weight (BMI P5–P85)	Insulin glargine dosing (initiation and follow-up), fasting glucose, post-discharge dose changes Comparison of descriptors (TBW vs. IBW)	Initial dose (~0.3 U/kg/day) and fasting glucose similar across groups No differences in dose changes	TBW best predictor for dose adjustment IBW underestimates requirements in overweight/obesity
	Flokas et al., 2020 [25] Cross-sectional observational	0 years–adolescents (T1DM); n = 2367; 217 obesity (BMI ≥ P95) 430 overweight (BMI P85–94.9) 1720 normal weight (BMI < P85)	Total daily insulin dose (U/kg/day) in patients with optimal glycemic control according to BMI category	Insulin dose per kg similar across groups (obesity: 0.86; normal weight: 0.84 U/kg/day) Lower probability of optimal control with higher BMI No differences in continuous SC insulin infusion and continuous glucose monitoring use	BMI not associated with higher insulin requirements/kg in patients with optimal control
	Palomo Atance et al., 2013 [26] Cross-sectional observational	6–12 years (T1DM); n = 115; 21 obesity (BMI ≥ P97) 12 overweight (BMI ≥ P90) 82 normal weight	Insulin dose (U/kg/day and U/m^2^/day) eGDR Predominance of glargine for basal insulin and aspartic for rapid-acting insulin	No differences in dose per kg (≈0.8–1.02 U/kg/day) Higher doses in overweight/obesity when adjusted by BSA (37.7 vs. 36.1 vs. 29.4 U/m^2^/day; *p* < 0.007) eGDR similar overall but lower in obese ≥ 11 years HbA1c similar LDL higher in obesity	BSA (m^2^) more sensitive than TBW (kg) to reflect insulin requirements in obesity
Semaglutide	Empirical studies (PK and/or clinical data)				
	Van Boxel et al., 2024 [27] Retrospective observational study	Children 10–18 years n = 50 BMI: SDS > 2 with at least one weight-related comorbidity	Semaglutide SC once weekly; Titrated over 8 weeks up to 1 mg/week Change in weight, BMI SDS, % TBW	At 12 months BMI SDS: decrease 0.54 ± 0.52 (*p* < 0.001) Body weight: decrease 9.7 ± 10.8 kg (*p* < 0.001) % total weight loss: 8.9 ± 10.0% (*p* < 0.001) AEs: mild gastrointestinal, 1 case of gallstones, 5 patients discontinued treatment	Semaglutide appears to be a safe and effective adjunct therapy for weight loss: 1 mg/week in children with obesity when used within a multidisciplinary weight management program
	PopPK/PBPK modeling studies				
	Machado et al., 2022 [28] Pharmacokinetic modeling	Virtual pediatric population 10–17 years (simulated). Subgroups: 10–12, 13–14, 15–17 years. Normal weight and obesity. (virtual n = 100 per subgroup in PBPK simulations) Diabetes treatment.	Development and validation of a PBPK model. Extrapolated from adults. Simulation of a single SC dose (0.25–1 mg) and steady state Physiological adjustments for obesity (fat mass, adipose blood flow, tissue volume)	PK data with a 0.5 mg SC dose Cmax: Higher values in children aged 10–14 years with a healthy weight, exceeding the ranges observed in adults. Children 10–12 years (normal weight): 0.076 µg/mL Children 10–12 years (obese): 0.041 µg/mL Tmax: Children with obesity showed delayed absorption, with Tmax values an average of 6.4 h higher than those of children with normal weight. Children 10–12 years (normal weight): 25 h/obese: 31.3 h AUC (0–840 h): Children 10–12 years normal weight: 11.55 µg·h/mL/obese: 11.6 µg·h/mL Body weight is inversely related to Cmax	PBPK modeling suggest that body weight is the most important covariate affecting the PK of semaglutide
Liraglutide	Danne et al., 2017 [29] Experimental controlled double-blind	Adolescent patients with obesity 21 patients	3 mg liraglutide (or maximum tolerated starting at 0.6 mg/week)/placebo (2:1) 5 weeks 14 liraglutide/7 placebo	Apparent Cl: Similar to adults with obesity. AUC 24 h: 1.1 times greater than in adults (90% CI 0.93; 1.31). No significant reduction in BMI-Z score and weight. AEs: Gastrointestinal	Inconclusive, few patients and a single center
	Mastrandrea et al., 2019 [30] Experimental controlled double-blind	Obese patients aged 7–11 years 24 patients	Randomized 2:1 Dosage: up to 3 mg Treatment duration: 7–13 weeks 24 (16 liraglutide/8 placebo) Only 20 patients completed the study (2 from each group were excluded)	CL/F in children: 0.69 L/h (95% CI: 0.60–0.82); adolescents (0.99 L/h) and adults (1.15 L/h). AUC (0–24 h): Children: 1161 h·nmol/L, Adolescents: 808 h·nmol/L, Adults: 697 h·nmol/L BMI z-score: significant reduction with liraglutide compared to placebo. No significant differences in weight. Fasting glucose: slight reduction with liraglutide. No differences in insulin and HbA1c. AEs: gastrointestinal, asymptomatic hypoglycemia.	In children aged 7–11 years, liraglutide behaves similarly to other populations, with linear and predictable PK
	Carlsson et al., 2021 [31] Meta-analysis of several clinical trials in children, adolescents, and adults	Adolescent patients with obesity	Meta-analysis of several clinical trials in children, adolescents, and adults Trials included: Adolescents: 56-week phase 3 study (n = 251) Children: phase 1 (n = 13) Adults: phase 1 and several phase 2/3 trials (total n > 4000)	A one-compartment model with first-order absorption and elimination was fitted ka: 0.0813 h^−1^ (fixed) CL/F: 1.01 L/h Vd/F: 13.8 L Weight was the main factor influencing exposure (age had no influence and sex a slight influence) Children (7–11 years): exposures were slightly higher than adolescents and adults, but this effect disappeared after adjusting for weight No differences were found according to pubertal stage	Adolescent exposures are similar to those of adults when the dose is adjusted to 3 mg Fixed dose of 3 mg without weight adjustment in adolescents
	Diene et al., 2022 [32] Experimental Double-Blind Controlled Study	Patients aged 5–17 years with PWS and obesity 24 children aged 6–11 years 31 adolescents aged 12–17 years	Randomized 2:1 3 mg liraglutide (or maximum tolerated dose); in <45 kg, up to 2.4 mg/day SC. Placebo Placebo: 16 weeks; liraglutide: 52 weeks Exercise and diet program.	Change in BMI SDS at weeks 16 and 52 was not significant in either children or adolescents. No significant changes in body weight were observed. Hyperphagia: In children: no significant changes. In adolescents (week 52), liraglutide reduced the total score (−6.42) and the drive score (−3.87) compared to no treatment. AEs: gastrointestinal, 2 cases of significant hypoglycemia	Inconclusive. The lack of efficacy could be due to the hypothalamic dysfunction characteristic of PWS
	Cornejo-Estada et al., 2023 [33] Systematic review	Patients aged 5–18 years with obesity treated with up to 3 mg/day of liraglutide Total patients 296	Systematic review of the efficacy of liraglutide for the treatment of obesity in the pediatric population	Three randomized clinical trials with a total of 296 patients were included. Only one of the trials included patients < 12 years (n = 24): the trial already discussed [23]	No conclusive data on the use of liraglutide for the treatment of obesity in pediatric patients
	Fox et al., 2025 [34] A Randomized Trial	Children aged 6 to <12 years n = 82 (56 liraglutide, 26 placebo) BMI ≥ P95	SC liraglutide once daily Starting dose: 0.6 mg/day (or 0.3 mg if <45 kg); Escalation: increments of 0.6 mg/week Maximum dose: 3.0 mg/day Placebo + lifestyle intervention 56-week treatment + 26-week follow-up Primary Outcomes: % change in BMI Secondary: % change in body weight, ≥5% BMI reduction	BMI change Liraglutide: −5.8% vs. Placebo: +1.6%; *p* < 0.001 Body weight change Liraglutide: +1.6% vs. Placebo: +10.0%; *p* = 0.001 ≥5% BMI reduction: Liraglutide: 46% vs. Placebo: 9%; OR: 6.3 (95% CI 1.4–28.8; *p* = 0.02) AEs: gastrointestinal (80%), with serious events in 12% and treatment discontinuation in 11% of patient	Liraglutide, in combination with lifestyle interventions, resulted in a significantly greater reduction in BMI and body weight compared with placebo in children aged 6 to <12 years with obesity
Metformin	Empirical studies (PK and/or clinical data)				
	Shiasi Arani et al., 2014 [42] Open-label controlled clinical trial	Obese children and adolescents (4–18 years) with NAFLD n = 376	4 groups: 1 g/day metformin, 1.5 g/day metformin, 400 IU or 800 IU/day vitamin E Duration: 4 months	BMI and BMI-SDS: No differences in any group Ultrasound improvement: with both metformin and vitamin E Vitamin E showed superior effectiveness to metformin AEs: only in the metformin group (gastrointestinal) and 9 dropouts	Inconclusive: There is no comparative data between the two doses of metformin
	Libman et al., 2015 [41] Double-blind controlled clinical trial	Adolescents with type 1 diabetes who are overweight or obese. n = 140	Two groups: insulin + 2000 mg/day metformin (n = 71) vs. insulin + placebo (n = 69) for 26 weeks	At 26 weeks, metformin reduces the amount of insulin needed, but without improving HbA1c. Reduction in weight, body fat, and BMI (modest benefits) AEs: gastrointestinal	Metformin should not be prescribed with the aim of improving glycemic control in adolescents with T1DM
	Liu et al., 2016 [43] Systematic review + meta-analysis	Adolescents with diabetes (n = 301) Overweight/obese subgroup (n = 124)	5 clinical trials were included. A meta-analysis was performed on HbA1c, insulin dose, BMI, and weight. A sub-analysis was conducted comparing overweight/obese adolescents to the general population.	The total daily insulin dose was reduced in both groups. The reduction in BMI and weight was significant in overweight/obese adolescents.	Doses of 2000 mg/day were less well tolerated (greater gastrointestinal effects) and not associated with better glycemic control in obese adolescents
	Handen et al., 2017 [44] Open extension trial	Children and adolescents (6–17 years) 52 participants With autism spectrum disorder treated with atypical antipsychotics and experiencing weight gain	Open-label extension trial of a 16-week, double-blind metformin/placebo clinical trial. 52 participants (22 metformin/30 placebo) Dosage: 6–9 years: 50 mg every 12 h Dosage: 10–17 years: 850 mg every 12 h	A significant reduction in BMI z-score was observed in participants who had not received metformin in the previous phase (−0.1) No significant changes were observed in those coming from metformin. AEs: Generally mild and transient gastrointestinal	Metformin effective in attenuating weight gain associated with antipsychotics in children and adolescents with autism spectrum disorder
	Sam et al., 2017 [45] PK Substudy Intervention Clinical Trial	Obese and hyperinsulinemic children aged 7–14 years with SLC22A1 genotype (28 of the 30 children)	2 groups: 18 reference (wild-type) 10 SLC22A1 variant carriers Metformin: 1000 mg/12 h (less 1 child with 500 mg/12 h	Single-compartment dual absorption model Population parameters: Cl/F = 68.1 L/h; V/F: 28.8 L SLC22A1 eGFR: only significant covariate Smaller reduction in trunk fat percentage in SLC22A1 variant carriers (*p* = 0.035) Reduction in total fat % and trunk fat mass: no differences	In obese children, metformin elimination depends primarily on renal function, not on the SLC22A1 genotype
	Lentferink et al., 2018 [37] Systematic review	Overweight/obese children and adults treated with metformin for 6 months. Exclusion criteria: diabetes mellitus, drug-induced obesity, NAFLD	Systematic review Cochrane (15 pediatric studies (22–151 participants)/14 adult studies between 32–3000 participants)	In the pediatric population: BMI reduction at 6 months (not all statistically significant) No decrease in BMI after 6 months Best responders: patients with hyperinsulinemia	The greatest differences occurred with doses of 1500–2000 mg/day The impact is less in children than in adults
	van Rongen et al., 2018 [35] PK sub-study of a Clinical Trial	Overweight or obese adolescents in treatment Age: 10–16 years Obesity: BMI-SDS > 2.3	22 patients Dosage: 500–1000 mg every 12 h Duration of treatment: 37 weeks	PK Data AUC: decreases with increasing weight AUC (0–8 h) (adjusted to 1000 mg): 603.5 mg·min/L Mean Cmax: 1.8 mg/L CL/F (1.17 L/min): increases significantly with TBW; higher than in non-obese children (0.55 L/min); similar to adults (1.3 L/min) Highly variable absorption; Tmax: 60–240 min No correlation between OCT1 and MATE1 genotypes with Cl/F, AUC, or Ka	The standard dose of 2 g/day may be insufficient in obese adolescents An adult dose of 3 g/day could be considered for obese adolescents
	Hui et al.,2018 [38] Systematic review, network meta-analysis	Overweight or obese adolescents and adults without diabetes mellitus 8461 participants	34 clinical trials with 8461 participants. Metformin at various doses, different interventions Efficacy: changes in BMI, BMI percentile (adolescents), and weight Safety	BMI percentile in adolescents: decreased significantly. BMI and weight: showed a trend (not always significant). Better results with doses ≥ 1000 mg/day, 3–6 months. Network meta-analysis - Adolescents: metformin 2000 mg/day—best pharmacological intervention. Overall efficacy (dose + time): 1000 mg/day for 3 months - Adults: 3000 mg/day for 6 months Safe in the short and medium term in people without diabetes mellitus	Modest but clinically useful effect, greater in adolescents than in adults Optimal dose: 1000 mg/day for 3 months Option for weight management when lifestyle interventions fail.
	Kay et al., 2001 [46] Experimental Double-Blind Controlled Study	Obese adolescents (BMI > 30 kg/m^2^) normoglycemic and hyperinsulinemic n = 24	Hypocaloric diet + metformin (850 mg/12 h)/placebo. 8 weeks n = 24 (1:1)	Weight and fat mass: Metformin resulted in greater weight loss than placebo (−6.1 ± 0.8 kg vs. −3.2 ± 2 kg) *p* < 0.01 and greater fat mass loss (−6 kg vs. −2.7 kg) *p* < 0.001 Insulin AUC decreased three times more in the metformin group than in the control group (*p* < 0.001) Lipids: Decreases (cholesterol, triglycerides, free fatty acids) were observed only in the metformin group Baseline glucose and glucose AUC showed no significant changes. AEs: Mild and transient gastrointestinal effects	Metformin at 850 mg/12 h is safe and effective in normoglycemic and hyperinsulinemic obese adolescents in reducing body weight along with a hypocaloric diet
	Wang et al., 2021 [40] Model-based meta-analysis	Children and adolescents treated with metformin for various conditions 1228 patients were included	Meta-analysis (11 obesity studies, 3 T1DM, 2 NAFLD, 2 precocious puberty) Doses used: Obesity: 1000–2000 mg/day T1DM: 1000–1700 mg/day NAFLD: 1000–1700 mg/day Precocious puberty: 425–850 mg/day	BMI is reduced by 4–10%. Non-dose dependent relationship. Time-dependent response (earlier in precocious puberty). Most effective in obesity and precocious puberty.	Inconclusive
	Masarwa et al., 2021 [39] Qualitative systematic review of randomized clinical trials.	Children and adolescents with obesity n = 1623	Metformin + lifestyle modification was compared to placebo + lifestyle modification. 24 clinical trials were included. High heterogeneity.	BMI and BMI z-score: favorable but modest effects. AEs: gastrointestinal. Limitations: heterogeneity, high loss to follow-up rates, variable quality, differences in lifestyle intervention.	No clear dose-response effect was identified in BMI, BMI z-score, or HOMA-IR. Fewer adverse effects were observed with doses ≤ 1000 mg/day than with 1500–2000 mg/day.
	PopPK/PBPK modeling studies				
	Ford et al., 2022 [36] Pharmacokinetic modeling	Simulations in adolescents without obesity, overweight, obese, and severely obese	The study started with an adult PBPK model and scaled up to children, incorporating physiological changes specific to childhood obesity, as well as real data from 3 previous studies in non-obese girls (n = 6), overweight/obese adolescents (n = 22) and severely obese children (n = 30) Main dosage simulations: 1000 (250/each group) and 500 for safety in obese individuals	CL/F was higher in obese adolescents (~1200 mL/min) than in non-obese children (~1000 mL/min) and similar to that of adults (larger renal volume and higher glomerular filtration rate) Simulated AUC in obese and severely obese adolescents was similar to that of adults for the same doses. Exposure was slightly higher in non-obese adolescents. PK’s of metformin in adolescents was similar to that of adults, supporting the use of adult-like doses.	−500 mg 2 times a day and 1000 mg 2 times a day: similar exposure in obese adolescents to adults −3000 mg doses are only safe if administered 3 times a day (not BID)

AEs: adverse events; AUC: area under the plasma concentration-time curve; BMI-SDS: body mass index standard deviation score; BSA: body surface area; Cl/F: apparent clearance; Cmax: maximum plasma concentration; T1DM: type 1 diabetes mellitus; eGFR: estimated glomerular filtration rate; HbA1c: hemoglobin A1c; HOMA-IR: homeostasis Model Assessment of Insulin Resistance; IBW: ideal body weight; Ka: absorption rate constant; LDL: low-density lipoprotein; MATE1: multidrug and toxin extrusion protein 1; NAFLD: non-alcoholic fatty liver disease; OCT: organic cation transporter 1; P: percentile; PBPK: physiologically based pharmacokinetic model; PD: pharmacodynamics; PK: pharmacokinetics; PWS: Prader–Willi syndrome; SLC22A1: solute carrier family 22 member 1 gene; TBW: total body weight; Tmax: time to reach maximum plasma concentration; Vd: volume of distribution; Vd/F: apparent volume of distribution.

#### 3.3.3. Oral Anticoagulants

Warfarin

Warfarin is almost completely absorbed, with an F of 100%, binds to albumin at 99%, and has a Vd of 0.14 L/kg. It is primarily metabolized by the CYP2C9 enzyme and subsequently excreted in urine (92%), with a t_½_ of approximately 40 h.

Moffett et al. [47] evaluated whether obesity prolongs the time to achieve a therapeutic International Normalized Ratio (INR) through a retrospective cohort study including 30 pediatric patients who received a warfarin loading dose according to institutional guidelines of 0.2 mg/kg (maximum 5 mg) or 0.1 mg/kg (maximum 2.5 mg) in the presence of drug interactions. Obese patients received lower initial and maximum doses per kg (*p* < 0.05), the absolute maximum dose in milligrams was higher in the obese group (7.2 ± 2.9 mg) than in the non-obese group (5.1 ± 1.7 mg) (*p* < 0.01) and the median time to reach the therapeutic range was doubled (6 vs. 3 days; *p* < 0.01), with no significant differences in adverse effects. The authors suggest that obesity may be associated with an increased Vd of warfarin, which alters the dose requirements needed to achieve effective concentrations, along with triglyceride levels, which correlates with higher vitamin K concentrations. It was concluded that traditional guidelines delay anticoagulation targets in obese children, suggesting that removal of initial dose capping may help optimize therapy in this population.

#### 3.3.4. Heparin and Other Antithrombotic Agents

Enoxaparin

Enoxaparin is a low-molecular-weight heparin (LMWH) most commonly used in pediatrics. It has an F close to 100% for the SC route, with a Vd of 0.12 L/kg in pediatrics, close to blood volume. It undergoes metabolism via desulfation and/or depolymerization and is excreted renally, with up to 40% eliminated by glomerular filtration, with a t_1/2_ of 4.5–7 h in adults, being shorter in pediatrics due to increased Cl.

Nine studies evaluating enoxaparin were identified, of which four addressed its use for thromboprophylaxis in pediatric patients. The most relevant aspects of these studies are summarized in Table 5.

Given the heterogeneity of the included studies, findings are presented according to the nature of the underlying evidence, distinguishing between empirical PK/clinical data and model-informed (PopPK/PBPK) approaches. The first six studies are based on empirical PK data derived from observational and clinical settings. Among these, Lewis et al. [48] retrospectively described three clinical cases of obese adolescents treated with enoxaparin for the prevention of thromboembolic events at standard adult doses of 40 mg/24 h for the prevention of venous thromboembolism (VTE). Two of the three patients required higher doses than those used in adults to achieve therapeutic anti-Xa levels; however, the doses per kg of TBW required were lower than the recommended 0.5 mg/kg of TBW used in standard pediatric thromboprophylaxis regimens.

Mushtaq et al. [49] conducted a prospective uncontrolled pilot study in 4 obese adolescents undergoing bariatric surgery treated with enoxaparin for the prevention of VTE. Enoxaparin dosing was stratified based on BMI. Obese patients achieved lower anti-Xa levels than non-obese patients, although within the predefined target range, as well as a lower AUC and a longer time to reach Tmax. The administered doses achieved the therapeutic target, but with lower drug exposure in obese patients.

Vaughns et al. [50] designed a non-controlled clinical trial as an extension of the previous pilot study, including 12 adolescents undergoing bariatric surgery treated with enoxaparin under the same conditions, concluding that BMI-based dosing is effective, achieving adequate anti-Xa levels for VTE prevention in 83% of patients.

Yim et al. [51] reported that standard enoxaparin prophylaxis (30 mg every 12 h) frequently resulted in subtherapeutic anti-Xa levels in obese pediatric patients, with over 50% failing to reach target ranges, while higher doses (mean ~66 mg/day) were required, particularly in those with severe obesity, without an increased incidence of thrombotic events and with low rates of bleeding.

Hoffman et al. [52] conducted a retrospective cohort study in which two treatment groups receiving reduced or standard doses of enoxaparin for the treatment of VTE and/or PE in adolescent patients were compared. They concluded that a reduced enoxaparin dose <0.90 mg/kg achieves therapeutic anti-Xa levels in most patients without increasing the risk of thrombosis progression or bleeding.

Richard et al. [53] conducted a cross-sectional study for the treatment of thromboembolic events in 30 obese patients and 30 non-obese patients dosed based on TBW. Obese patients had significantly higher initial anti-Xa levels than non-obese patients. However, these levels remained within the therapeutic range, and mean maintenance doses based on TBW were lower in obese patients.

The following three studies are based on PK modeling and should therefore be considered exploratory and require prospective validation before informing clinical practice.

Gerhart et al. [54] using real-world data and PBPK modeling, suggest that children with obesity had significantly higher anti-Xa concentrations than non-obese patients at the same dose, with simulations indicating up to ~20% higher exposure under TBW-based dosing due to reduced weight-normalized Cl, this may be interpreted as indicating that excess body mass has little impact on drug clearance; therefore, dosing based on FFM or AjBW is suggested. Similarly, Carreño et al. [55] in a PopPK study, found that TBW-based dosing increased anti-Xa exposure in obese children (13–20%), whereas FFM-based dosing resulted in more comparable exposure across obesity status. Overall, both studies demonstrated higher probability of exposure with TBW-based dosing in obesity and improved comparability using FFM-based approaches.

Derbalah et al. [56] developed a PK model proposing a dosing regimen for the treatment of VTE, PE, and coronary disease based on FFM, consisting of a loading dose (1.8 mg/kg of FFM) followed by a maintenance dose (1.2 mg/kg of FFM twice daily), was associated with an improved probability the rate of achieving the desired therapeutic activity. This approach may simplify the need to categorize patients by weight and simplifies dose calculation in clinical practice. However, robust prospective validation is warranted before this regimen can be recommended in practice.

Unfractionated heparin

Unfractionated heparin (UFH) has a Vd of 0.07 L/kg; this corresponds to the extent of plasma volume, and it appears that the distribution of heparin in the body is confined to the intravascular space. Plasma protein binding is extensive to low-density lipoprotein, globulins (including the α-globulin antithrombin III), and fibrinogen. Cl is 0.5 to 0.6 mL/kg/min, and it undergoes metabolism by N-desulfation and by the reticuloendothelial system.

Three studies in obese pediatric patients were identified, as detailed in Table 5. Taylor et al. [57] conducted a study comparing UFH dosing for treatment based on TBW, concluding that children with obesity require lower UFH doses (both initial and maintenance) to achieve the desired anticoagulation and did not present a higher frequency of supratherapeutic anticoagulation. This is consistent with the hypothesis that UFH distribution is greater in blood volume and lower in adipose tissue, supporting dose adjustments in obese pediatric patients.

Moffett et al. [58] conducted a study comparing dosing efficacy in obese versus non-obese patients undergoing cardiac catheterization, observing no significant differences in anticoagulant response based on activated clotting time (ACT). Therefore, they inferred that dose adjustments are not required in obese patients. However, this finding contrasts with observations in adults, where obese patients show an increased response to UFH. Consequently, they concluded that further studies are needed and recommended monitoring anticoagulant response using anti-Xa rather than ACT, as the latter may not be an appropriate marker.

Kuhn et al. [59] conducted a cohort study including patients younger than 21 years receiving UFH infusion based on TBW without dose capping, guided by anti-Xa levels. A correlation between BMI and anti-Xa levels was observed. Obese patients achieved higher heparin levels with dosing based on actual body weight, which did not appear to increase the risk of clinically significant bleeding. However, caution is advised, and prospective studies are needed to confirm whether avoiding dose capping in obese children is truly safe.

Finally, a retrospective cohort study conducted by Larouche et al. in 2026 was identified [60] to evaluate the impact of obesity on heparin dosing and clinical outcomes in pediatric patients with VTE. The study included 212 patients aged <18 years (23.6% obese/overweight) treated with LMWH or UFH, comparing dosing adjustments and outcomes between groups. Obese/overweight children required significantly lower dose increases from initial dosing (3.2% vs. 11.3%, *p* < 0.001), with more frequent dose reductions (11.1% vs. 2.1%). Rates of thrombotic recurrence/progression (12.0% vs. 10.5%) and clinically relevant bleeding (2.0% vs. 3.1%) were low and similar between groups. The authors concluded that obesity is associated with lower anticoagulation requirements, supporting the need for individualized dosing strategies in this population.

Dalteparin

Dalteparin has a high F of 87%, minimal plasma protein binding, and has a Vd in pediatrics of 0.16–0.18 L/kg. It is primarily eliminated via the renal route, with a t_1/2_ in pediatrics of 2.25–6.28 h.

Warad et al. [61] conducted a retrospective analysis of dalteparin use in pediatrics, including obese children in whom dalteparin was dosed based on AjBW. Dalteparin was shown to be an effective and safe option for the treatment and prophylaxis of VTE in pediatric patients. Infants (<1 year) require significantly higher doses per kg of body weight to achieve therapeutic anti-Xa activity levels compared to older children and adolescents. Obese patients received dosing based on AjBW, achieving therapeutic levels without hemorrhagic complications. However, no cohort analysis comparing obese and non-obese patients was performed, and the method used for dose adjustment was not specified. Detailed results are presented in Table 5.

Fondaparinux

Fondaparinux has an F of 100% for the SC route, with distribution in blood and minor distribution into extravascular fluid, a Vd of 7–11 L, and 94% plasma protein binding, mainly to antithrombin. It is excreted unchanged 77% via the renal route, with a t_1/2_ of 17–21 h.

Johnson et al. [62] reported a case of an obese pediatric patient, in whom dose reduction was required due to bleeding events, likely influenced by multiple confounding factors (critical illness, comorbidities, and renal impairment). Fondaparinux may represent an alternative in pediatric patients with heparin-induced thrombocytopenia, although close monitoring is warranted. Detailed results are presented in Table 5.

**Table 5:** Summary of studies describing the dosing and PK and/or PD aspects of antithrombotic agents.

	Study/Design	Patients/Sample Size				Methods	Results				Dosing Conclusions
Enoxaparin	Empirical studies (PK and/or clinical data)										
	Lewis et al., 2011 [48] Case report	Case 1: 16 years old; weight: 358.6 kg; BMI: 105.9; Immobility due to morbid obesity Case 2: 16 years old; 294 kg; BMI: 95.7; Status asthmaticus in intensive care. Case 3: 11 years old; 81.5 kg; BMI: 29.9; Perforated appendicitis with abscess Prophylaxis of thromboembolic events				Case 1: Initial dose 40 mg/day (0.11 mg/kg) Case 2: Initial dose 40 mg/day (0.14 mg/kg) Case 3: Initial dose 40 mg/day (0.49 mg/kg) Target anti-Xa level: 0.1–0.3 IU/mL	Case 1: Anti-Xa < 0.02 (subtherapeutic) Final adjusted dose 100 mg/day (0.35 mg/kg) Case 2: Anti-Xa not measured; final adjusted dose 45 mg/day (0.15 mg/kg) Case 3: Anti-Xa: not measured; final adjusted dose 40 mg/day (0.49 mg/kg) AEs: 1 patient experienced a mild adverse effect: hematoma at the injection site.				The doses required per kg of weight to achieve the anti-Xa target are lower in 2 of the patients than the pediatric dose recommendations of 0.5 mg/kg; but higher than the standard adult dose
	Mushtaq et al., 2015 [49] Prospective pharmacokinetic clinical study (non-randomized)	4 obese adolescents undergoing bariatric surgery received enoxaparin for VTE prophylaxis				Dosing was stratified according to BMI: BMI ≤ 50 kg/m^2^: 40 mg SC every 12 h BMI > 50 kg/m^2^: 60 mg SC every 12 h Plasma anti-Xa activity was used as a surrogate PK endpoint for enoxaparin for the calculation of Cmax, Tmax, and AUC	Anti-Xa levels: obese adolescents had significantly lower levels than non-obese young patients despite receiving similar doses Tmax: this was longer in obese patients, with a mean of 6 h vs. 3 h in non-obese young adults Despite the lower anti-Xa levels, no thrombotic events were observed in the patients				The current regimen of 40 mg (BMI ≤ 50 kg/m^2^) and 60 mg (BMI > 50 kg/m^2^) achieves anti-Xa levels within the recommended therapeutic range, with lower drug exposure in obese patients
		Patient	Age	Weight	BMI		Group	Cmax anti-Xa (IU/mL)	Tmax (h)	AUC (IU/mL)	
		1	16	174	58.1						
		2	16	117.8	44.9		Obese adolescents	0.2–0.3	4.21–7.51	1.08–2.02	
		3	18	145.7	51.6						
		4	17	149.8	55.7		Young adults	0.38–0.53	3.0–3.5	2.63–4.92	
		3 comparator groups were based on historical data from non-obese young adults.									
	Vaughns et al., 2019 [50] Estudio clínico prospectivo PK (cohorte con muestreo intensivo)	Continuation study of the previous one, with the same objective and methodology, expanded to 12 adolescent patients undergoing bariatric surgery.				Same dosing regimen. Blood samples were collected at the following time points: pre-dose, and 1, 2, 4, 6, and 12 h after administration for the measurement of plasma anti-Xa activity	Mean BMI: 49.9 kg/m^2^ (range: 38.4–58 kg/m^2^). Mean body weight: 140.8 kg (range: 93.7–174 kg). No VTE events or significant bleeding complications occurred during hospitalization. Anti-FXa Cmax: median 0.205 IU/mL (range: 0.14–0.30 IU/mL). Tmax: median 5.67 h (range: 3.78–7.52 h). AUC: median 1.00 IU/mL (range: 0.42–1.67 IU/mL). 83% of patients achieved adequate anti-Xa levels for VTE prophylaxis				Same as the previous study.
	Yim et al., 2024 [51] Retrospective cohort observational	Patients ≤ 18 years n = 116 BMI ≥ P95; BMI P95–P99; BMI ≥ body weight (≤100 kg vs. >100 kg). Prophylactic				Initial standard prophylactic dose: Enoxaparin 30 mg every 12 h Dose adjustments based on anti-Xa levels 0.2–0.4 IU/mL	Subtherapeutic anti-Xa (<0.2 IU/mL): 53%: BMI > P99 54%: >100 kg Dose required to reach target anti-Xa: 66 ± 13 mg/day AEs: Major bleeding: 1 case; Minor bleeding: 3 cases				Adolescents with severe obesity require higher enoxaparin doses than standard prophylactic regimens An initial dose of 40 mg every 12 h in adolescents with BMI ≥ P99 or body weight > 100 kg
	Hoffman et al., 2017 [52] Retrospective cohort observational study	Patients > 12 years and <18 years with overweight and obese BMI > P85 for sex and age. On VTE and/or PE treatment. A total of 30 patients (19 on reduced dose and 11 on standard dose).				Comparison between patients on reduced dose (RD < 0.90 mg/kg/12 h) and patients on standard dose (SD ≥ 0.90 mg/kg/12 h). Therapeutic target anti-Xa level is between 0.5–1.0 IU/mL.	Baseline anti-Xa levels: RD group: 0.68 ± 0.20 IU/mL. SD group: 0.73 ± 0.28 IU/mL (*p* = 0.640). No significant differences. Dose adjustments and final anti-FXa levels: RD group: 53% did not require dose adjustments. SD group: 81.8% required dose adjustments (*p* = 0.063). Dose reduction in the SD group: 72.7% reduced the final dose compared to the initial dose (*p* = 0.009). Thrombosis progression: 2 patients in the RD group (10.5%) and 1 patient in the SD group (9.1%) (*p* > 0.999). Bleeding: 2 patients in the RD group (10.5%) and 3 patients in the SD group (27.3%) (*p* = 0.327). All bleeding episodes were minor. At the end of treatment: 83% of patients were in the RD group with a dose of 0.73 ± 0.11 mg/kg.				The reduced dose of enoxaparin (<0.90 mg/kg) achieved therapeutic levels of anti-Xa in most patients without increasing the risk of progression of thrombosis or bleeding.
	Richard et al., 2013 [53] Retrospective cohort observational study	Patients >2 years old–adolescents. 30 obese (BMI > 95P) versus 30 non-obese (BMI: 25th–75th percentile) Treatment of thromboembolic events.				Dosage: 1 mg/TBW/12 h SC. Measure anti-Xa levels 4–6 h after the second dose. Target anti-Xa level: 0.5–1 IU/mL	Anti-Xa levels: Obese patients had significantly higher baseline levels than non-obese patients (0.67 ± 0.27 vs. 0.53 ± 0.24 units/mL, *p* = 0.028). Levels were within the therapeutic range. The mean therapeutic dose was lower in obese patients compared to non-obese patients (0.81 ± 0.19 vs. 1.1 ± 0.4 mg/kg, *p* = 0.005). 1 obese patient experienced mild gingival bleeding.				Obese patients may require dose reductions to avoid supratherapeutic levels and bleeding events.
	PopPK/PBPK modeling studies										
	Gerhart et al., 2022 [54] Retrospective cohort observational	Obese children aged 2 years to adolescents receiving enoxaparin treatment or prophylaxis 596 children were included, with 2825 measurements of anti-Xa activity 415 obese vs. 104 non-obese				Data from electronic health records of the Pediatric Trials Network, a multicenter database from nine US hospitals between 2013 and 2017, and PBPK models were used to characterize differences in enoxaparin exposure between children with and without obesity. Anti-Xa activity was used as an indirect biomarker of enoxaparin concentration in blood.	Anti-Xa levels at 4 h post-dose: Without obesity: 0.67 ± 0.28 IU/mL. With obesity: 0.78 ± 0.29 IU/mL (*p* < 0.001). Children with obesity had 20% greater exposure. According to PKPB modeling. Total Vd and Cl: higher in children with obesity in absolute terms, but lower when normalized for weight. Dose based on TBW: 20% greater enoxaparin exposure in children with obesity aged 12 to 18 years. Dose based on FFM: better correspondence in anti-Xa levels between children with and without obesity.				Using FFM as the basis for dosing may be a better strategy to achieve comparable exposure in children with and without obesity.
	Carreño et al., 2024 [55] PopPK study based on retrospective real-world data	Aged 1 day to 18 years n = 1293 BMI ≥ P95 The specific indication (prophylaxis vs. treatment) was not reported or stratified in the analysis.				NONMEM PopPK analysis One-compartment model with first-order absorption and elimination Scaling: TBW (<2 years) FFM (≥2 years)	Model: one-compartment with linear absorption/elimination CL/F: 9.03 mL/h Vd/F: 179 mL Allometric scaling: CL/F exponent: 0.75; Vd/F exponent:1 20% higher anti-Xa concentrations in obese vs. non-obese FFM: Reduced risk of supratherapeutic concentrations				FFM-based dosing and twice-daily regimens may reduce overdosing risk
	Derbalah et al., 2022 [56] Population Pharmacokinetics PopPK Study: Retrospective Cohort Observational Study	160 patients where the performance of published PK models is evaluated in a dataset of normal weight and overweight/obese children treated with enoxaparin				Comparison of PK models (two of them assuming that enoxaparin elimination is proportional to TBW and another model developed by the researchers: a one-compartment model with linear elimination where they assumed a Vd proportional to FFM and Cl: with an allometric exponent of 0.712	Population PK parameters normalized to 20 kg of FFM CL mL/h: 4.09 (3.81–4.39) Allometric exponent in Cl (FFM): 0.712 (0.66–0.76) Vd mL: 34.3 mL (29–39.6) Proposed dosing regimen Loading dose: 1.8 mg/kg FFM (71.5% and 72.5% of normal weight and obese patients within therapeutic range, respectively) Maintenance: 1.2 mg/kg FFM/12 h (77.5% and 76.7% of normal weight and obese patients within therapeutic range, respectively).				A dosing regimen based on FFM is proposed for the loading and maintenance doses.
Unfractionated heparin	Taylor et al., 2013 [57] Retrospective cohort observational study	Children aged 2 years to adolescents. 25 obese patients (BMI > P95) 25 normal-weight patients (BMI 27th–75th percentile) Treatment of thromboembolism, prosthetic value, stroke, atrial fibrillation, antiphospholipid syndrome, and presence of a pacemaker				Patients receiving UFH infusion for therapeutic anticoagulation in TBW At least one aPTT or anti-Xa value measured ≥ 4 h after the start of the infusion is determined. aPTT (70–101 s) anti-Xa (0.35–0.7 U/mL)	Initial UFH dose (U/kg/h) Obese children: 17.4 vs. 20.2 in non-obese children (*p* = 0.013) Maintenance dose: Obese children: 19.1 vs. 24.3 in non-obese children (*p* = 0.033) Initial response: There was no difference in initial aPTT between the two groups Obese children had higher initial anti-Xa levels (0.45 vs. 0.29 U/mL, *p* = 0.045) Time to achieve stable anticoagulation: Shorter in obese children with anti-Xa antibodies: 27.3 vs. 44 h (*p* = 0.045) No significant difference in time to aPTT (39.2 vs. 42.6 h, *p* = 0.807). AEs: Only one case of postoperative bleeding in the non-obese group				Dosage adjustments are necessary because obese patients require lower doses of UFH (both initial and maintenance) to achieve the desired anticoagulation.
	Moffett et al., 2011 [58] Prospective cohort observational study (multicenter cohort sub-analysis)	Children aged 2 years to adolescents. 39 obese patients (BMI > P95) 39 normal-weight patients Undergoing cardiac catheterization				Standard dose in cardiac catheterization: 50–100 U/kg/dose by TBW Target ACT: ≥250 s at 1 h post-bolus Maximum recommended: 5000 U	UFH dose: no significant differences between the administered doses ACT measurement time after UFH: Similar in both groups. Obese: 56 ± 26 min vs. Non-obese: 52 ± 26 min; *p* = 0.59 Baseline ACT values similar between obese and non-obese: Obese: 121.9 ± 31.3 s. vs. Non-obese: 126.6 ± 35 s.; *p* = 0.53 (Not significant) There were no major bleeding events in either group				Adjusting the UFH dose in obese children is not justified in this context. However, monitoring with anti-Xa antibodies is recommended instead of ACT.
	Kuhn et al., 2021 [59] Prospective Cohort Observational Study (multicenter observational cohort sub-analysis)	Children aged 2 years to under 21 years 22 obese patients (BMI > P95) 34 normal weight patients				Pediatric patients who received a TBW-based UFH infusion, without a maximum dose limit (“no dose capping”), over a 20-month period at Nationwide Children’s Hospital The dose was adjusted using an institutional nomogram based on anti-FXa levels, with a therapeutic target of 0.35–0.7 IU/mL	Supratherapeutic baseline anti-Xa levels were associated with significantly higher BMI: Median BMI: 28.7 vs. 20.4 (*p* = 0.0042) BMI was associated with a higher likelihood of supratherapeutic levels at any time point: OR: 1.17 (95% CI: 1.05–1.30; *p* = 0.0038) No significant difference was observed in the rate of clinically relevant or major bleeding between groups (*p* = 0.99). Only 2 major bleeding events were recorded, 1 in each group				Obese patients require a lower dose of UFH to avoid supratherapeutic doses of heparin
Dalteparin	Warad et al., 2015 [61] Retrospective cohort observational study	Patients aged 0 to 18 years. Patients were grouped into three age categories: 132 non-obese 21 obese patients: defined as those whose actual weight exceeded their ideal weight for age, sex, and height by more than 20%				Review of EHRs at a single institution over 1 year Dalteparin dosage in obese patients: Calculated using the AjBW	There were no significant differences in: The final doses required to reach therapeutic levels and in the frequency of bleeding events Obesity did not impact the need for higher doses or safety				The dose in obese patients by AjBW and the presence of malignancy did not significantly affect the required doses of dalteparin
Fondaparinux	Johnson et al., 2013 [62] Clinical case	Patient, 9 years old, 68 kg and Height: 147 cm. BMI 31.5 kg/m^2^) Presented multiple intestinal perforations, massive bleeding, hemorrhagic shock that developed into HIT				Fondaparinux SC was initiated at a dose of 7.5 mg daily (0.11 mg/kg). A fondaparinux-specific anti-factor Xa assay was implemented for monitoring	The patient was successfully treated with SC fondaparinux but required frequent dose adjustments due to bleeding episodes. Initial dose: 7.5 mg daily (0.11 mg/kg/day). Final dose: reduced to 4.5 mg daily (0.066 mg/kg/day) following hemorrhagic complications. The plasma concentration of fondaparinux (specific anti-factor Xa) was below the therapeutic range in adults (1.2–1.26 mg/L), but the dose was not increased due to the history of bleeding				The required dose may be lower in obese children or those with kidney dysfunction, as was observed in this case

ACT: activated clotting time; AEs: adverse events; AjBW: adjusted body weight; anti-Xa: anti-activated factor Xa; aPTT: activated partial thromboplastin time; AUC: Area under the plasma concentration-time curve; BMI: body mass index; Cl: total body clearance; Cl/F: Apparent clearance; Cmax: maximum plasma concentration; EHRs: electronic health record; FFM: fat-free mass; HIT: heparin-induced thrombocytopenia; NONMEM: nonlinear mixed effects modeling; P: percentile; PE: pulmonary embolism; PBPK: physiologically based pharmacokinetic models; PD: pharmacodynamics; PK: pharmacokinetics; RD: reduced dosing; SC: subcutaneous; SD: standard dosing; TBW: total body weight; Tmax: time to reach maximum concentration; UFH: unfractionated heparin; Vd: volume of distribution; Vd/F: apparent volume of distribution; VTE: venous thromboembolism.

#### 3.3.5. Cardiac Stimulants, Including Glycosides

Two studies evaluating IM epinephrine administration in the context of anaphylaxis were identified. The first, conducted by Duong et al. [63], aimed to determine the needle length required to ensure effective IM injection in the thigh in pediatric patients with different weights and BMI, based on ultrasound measurement of the distance from the skin to the muscle compartment and bone. The data showed that children with higher BMI have a greater likelihood that the autoinjector needle does not reach the muscle, placing these patients at risk of SC administration, with the potential for reduced clinical efficacy.

Brown et al. [64] conducted a review to analyze dose, injection depth, and the design of epinephrine autoinjector devices in the treatment of anaphylaxis, particularly in low-weight pediatric patients (<15 kg) and patients with obesity. They concluded that individual anatomical factors should be considered when selecting an autoinjector devices, rather than relying solely on body weight, especially in individuals with obesity (Table 6).

#### 3.3.6. Antihypertensive Agents

Regarding antihypertensive agents, two studies were identified. One, conducted by Hanafy et al. [65], included calcium channel blockers (CCB) such as nifedipine and amlodipine, and angiotensin-converting enzyme inhibitors (ANGI) such as enalapril and ramipril, with the aim of describing the effect of obesity on the PD of these antihypertensives in children with renal disease. The study concluded that obesity reduces the efficacy of CCB in children with renal disease, particularly in lowering systolic blood pressure, suggesting the potential need for higher doses in obese patients. Corticosteroid treatment also reduced the diastolic response to CCB, which may be related to their effects on sodium retention, catecholamine sensitivity, and activation of the central nervous system. Obesity did not affect the response to ANGI, which is consistent with findings in adults, and these drugs appear to maintain their efficacy even in the presence of inflammation.

The second study was a PK modeling study with amlodipine conducted by Burhanuddin et al. [66], aimed at evaluating how pediatric obesity affects the PK of amlodipine using PBPK models, as well as determining the necessary dose adjustments in children with obesity. It was found that Vd at steady state and Cl were significantly higher in obese children compared to non-obese children across all age groups, both with fixed dosing and TBW-based dosing. This contributes to reduced systemic drug exposure in obese patients. The study concluded that, to achieve the same concentrations as in normal-weight patients, dosing in obese patients aged 2–12 years should be based on TBW; alternatively, if fixed dosing is used, obese patients should receive 1.25–1.5-fold higher doses than non-obese patients, without exceeding 10 mg/day. Nevertheless, it should be acknowledged that a key limitation lies in the model-based nature of the study, as PBPK simulations, despite validation, cannot fully capture the complexity and heterogeneity of real-world paediatric obesity (Table 6).

#### 3.3.7. Neuromuscular Blocking Agents

Succinylcholine

Succinylcholine is rapidly distributed throughout the extracellular fluids in the body and is rapidly hydrolyzed by plasma cholinesterase to form succinylmonocholine (a metabolite with significantly lower neuromuscular blocking activity, approximately 20- to 80-fold less potent) and choline. Less than 10% is excreted unchanged in the urine, and the plasma t_1/2_ is approximately 3 min.

Only one study on succinylcholine was identified, conducted by Rose et al. [67], aimed at determining the potency of succinylcholine in obese adolescents (BMI > 30 kg/m^2^), expressed as the dose required to achieve 50%, 90%, and 95% depression of neuromuscular function (ED50, ED90, and ED95), calculated based on TBW. The study concluded that the potency of succinylcholine in obese adolescents is similar to that in non-obese adolescents when dosing is based on TBW (Table 6).

#### 3.3.8. Aminoglycoside Antibiotics

Gentamicin exhibits high hydrophilicity, with predominant distribution in extracellular fluids, high concentrations in the renal cortex, and minimal penetration into the cerebrospinal fluid. It has a Vd of 0.4 L/kg in infants, decreasing to 0.3 ± 0.1 L/kg in adolescents, <30% plasma protein binding, and a t_1/2_ of 4 h in infants and 1.5 ± 1 h in adolescents. It is excreted unchanged in the urine in more than 70%. Two studies on gentamicin were identified. Table 7 summarizes the included studies.

The retrospective cohort analysis conducted by Choi et al. [68] showed that obese pediatric patients develop significantly higher serum gentamicin concentrations and a markedly lower Vd compared to normal-weight individuals. These findings increase the risk of achieving supratherapeutic levels even with lower doses per kilogram, supporting the need for individualized dosing and TDM to ensure clinical safety and optimize treatment efficacy in this population.

Another study by Moffett et al. [69], a population PK analysis including 520 pediatric patients, identified that FFM with allometric scaling best describes gentamicin disposition, outperforming TBW and other traditional adjustment formulas. The model-based results suggested that the use of FFM improved the prediction of Cl and Vd, potentially facilitating the achievement of therapeutic targets. These results are consistent with the hydrophilic nature and limited distribution of aminoglycoside agents.

#### 3.3.9. Glycopeptide Antibiotics

Vancomycin is widely distributed in body tissues and fluids, except in the cerebrospinal fluid. It has a Vd of 0.56 L/kg in infants, 0.47 L/kg in children, and 0.49 L/kg in adolescents, with 55% plasma protein binding. It is eliminated by glomerular filtration, with a t_1/2_ ranging from 2.8 h in infants to 3.2 h in adolescents.

A total of 11 articles describing the behavior of vancomycin in obese pediatric patients were identified. The studies are summarized in Table 7. In light of the heterogeneity across studies, findings are presented according to the nature of the underlying evidence, distinguishing between empirical PK/clinical data and model-informed (PopPK/PBPK) approaches. The first seven studies were based on empirical PK/clinical data, whereas the remaining four relied on PopPK modeling approaches.

Moffett et al. [70] evaluated the impact of obesity by determining vancomycin trough levels in a retrospective study of 24 matched patients receiving 15 mg/kg based on TBW. The results showed no significant differences in trough concentrations. The Vd and t_1/2_ calculated in 4 obese patients were similar to those in normal-weight patients, supporting dosing based on TBW.

Miller et al. [71] compared, in a retrospective study of 187 children (2–17 years), the achievement of vancomycin trough levels (5–15 µg/mL). PK results showed higher trough levels in overweight/obese patients (9.6 vs. 7.4 µg/mL; *p* = 0.03); however, clinically, obese patients were less likely to achieve the target (OR 0.34; *p* = 0.037). It was concluded that every-8-h dosing regimens are more effective, extending to every 6 h in more complicated infections. Nevertheless, routine monitoring is recommended in obese patients due to the greater difficulty in achieving therapeutic levels.

Nassar et al. [72] prospectively evaluated, in 51 children, the effectiveness of a dosing regimen administered every 12 h according to weight categories. Only 3% achieved target trough levels, with no significant differences in PK parameters between the studied groups. It was concluded that excess body weight does not alter vancomycin PK in pediatrics and that current regimens are insufficient, requiring higher and more frequent dosing.

Heble et al. [73] compared, in 126 children, the effect of vancomycin dosing based on TBW, finding significantly higher concentrations in patients with obesity (14.4 vs. 10.5 µg/mL). Clinically, this resulted in excessive levels (>20 µg/mL) with a potential risk of nephrotoxicity. It was concluded that dosing based on TBW in obese patients may increase initial levels, making TDM essential to adjust therapy and avoid toxicity.

Eiland et al. [74] conducted a study in 98 children to optimize vancomycin dosing according to body weight. Through a retrospective review, only 14.2% achieved target levels, with no significant differences in the required dose between normal-weight and obese patients. The risk of renal injury was minimal, occurring in only one patient. It was concluded that TBW should be used to calculate doses and that current regimens are often insufficient to achieve the desired therapeutic ranges.

Khare et al. (2021) [75] analyzed, in a multicenter retrospective study including 1099 children, the influence of weight metrics on vancomycin dosing, finding that 75% presented subtherapeutic trough levels. The results indicated that children with obesity achieved higher trough concentrations and AUC (9.2 µg/mL and 409 µg·h/mL) than normal-weight children, despite receiving lower doses based on TBW, with no significant renal toxicity reported due to the generally low exposure observed. It was concluded that AUC/MIC is the most appropriate predictor of drug exposure and that TBW is an effective metric for its calculation across all weight categories.

Khare et al. (2020) [76] conducted a meta-analysis including 521 children to evaluate the impact of obesity on vancomycin dosing. The results showed that the use of TBW leads to trough levels 2.2 µg/mL higher and lower drug Cl compared to normal-weight children. Although no significant differences in nephrotoxicity were identified due to limited data, the available meta-analysis data have several limitations, including variability in methodological quality, small sample sizes, and heterogeneity in dosing practices.

Le et al. [77] evaluated the most appropriate body size descriptor for vancomycin PK in 174 children using a Bayesian one-compartment model. Obese patients received a mean dose of 41.9 mg/kg/day, with a Cl of 0.11 L/kg/h and a Vd of 0.56 L/kg, with no significant differences compared to controls; therefore, the minor PK differences observed may not warrant variations in clinical dosing between the studied groups.

Moffett et al. (2019) [78] conducted a retrospective population PK modeling study including 196 pediatric patients weighing ≥70 kg to determine an optimal vancomycin dosing approach. Using NONMEM, a one-compartment model including FFM and serum creatinine as covariates best described CL model-based simulations suggested that 20 mg/kg based on FFM every 6 h was the regimen most likely to achieve the predefined target (AUC of ≥400 mg·h/L and a trough concentration of <20 mg/L), potentially reducing the risk of toxicity or underexposure associated with inappropriate dosing. However, these findings should be interpreted cautiously until supported by prospective clinical validation.

Smit et al. [79] characterized vancomycin PK in 1892 children (1–18 years) with varying degrees of obesity and renal function to develop dosing guidelines aimed at achieving an AUC of 400–700 mg·h/L. Using a two-compartment model, they demonstrated that Cl depends on TBW and renal function (creatinine clearance according to the Schwartz formula), proposing a dosing strategy that achieves therapeutic levels from the first day and minimizes the risk of nephrotoxicity compared to previous guidelines that overexpose obese patients or those with renal impairment. It was suggested that integrating body weight and renal function into dosing ensures effective and safe exposure across the pediatric population.

Zhang et al. [80] investigated vancomycin PK in 125 adolescents and 81 adults with overweight or obesity to assess whether adult Cl predicts that of adolescents. Using population PK modeling, they determined that Cl is higher in adolescents and correlates more strongly with standard body weight (based on age and height) than with excess weight due to obesity; additionally, Cl was 21% higher in male adolescents and decreased with age in adults. It was suggested that adult Cl may not reliably predict adolescent Cl, and therefore dosing should not be directly extrapolated between these populations.

Considering the previous studies, it can be stated that in children with obesity, absolute Vd tends to increase with TBW, while weight-normalized Vd decreases due to the low solubility of vancomycin in adipose tissue. At the same time, absolute Cl increases, possibly related to increased cardiac output and renal blood flow associated with obesity; however, when normalized by body weight, Cl values are typically lower or similar in obese patients, and remain higher in adolescents than in adults. These PK parameters correlate well with TBW or allometric weight and renal function (Schwartz equation), supporting AUC/MIC-guided monitoring over trough concentrations to optimize therapy. However, as outlined above, part of the evidence supporting these findings appears to rely primarily on PK modeling approaches, with limited direct clinical data available. 

#### 3.3.10. Antiepileptic Drugs

Valproic acid

Five studies in obese pediatric patients receiving antiepileptic drugs were identified: three with valproic acid (VPA) and two with phenytoin (PFH). The studies are described in Table 8.

Sodium valproate has an F close to 100%; protein binding is saturable (74–93%), mainly to albumin, with a Vd of 0.14–0.23 L/kg. The main metabolic pathway is glucuronidation, with a t_1/2_ of 16 h in adults and 1–13 h in pediatrics.

The study conducted by Suemaru et al. [81] with carbamazepine (CBZ) and VPA included children older than 12 years. VPA concentrations decreased slightly with increasing IBW, but not with TBW or BMI. This suggests that IBW could be the most appropriate descriptor for VPA dose adjustment, as the other descriptors did not correlate well with serum levels. However, these findings are not conclusive due to the very small sample size (5 obese/12 normal-weight), and TDM is recommended in patients at extremes of body weight.

Arya et al. [82] conducted a secondary analysis derived from a randomized double-blind clinical trial to determine whether BMI affects treatment response to ethosuximide (ETX), VPA, and lamotrigine (LTG) in patients with absence seizures. A higher prevalence of overweight and obesity was observed in children with absence epilepsy compared to controls. In obese children, efficacy was greater with ETX and VPA compared to LTG, and this pattern was more pronounced with higher BMI z-scores, as shown in regression analysis. Regarding PK parameters (Cmin and AUC), no significant differences were observed in more than 90% of children in each treatment group for any of the three drugs. Although obese children received higher absolute doses, blood levels did not differ significantly. Therefore, differences in efficacy are not explained by PK but are likely related to other factors such as PD, biological, or metabolic mechanisms not yet fully understood. One potential factor could be drug lipophilicity: ETX is the least lipophilic (log *p* = 0.38), whereas LTG (log *p* = 1.87) and VPA (log *p* = 2.75) are more lipophilic; however, these differences alone are insufficient to explain the observed effect.

Good et al. [83] conducted a cohort study to determine whether an oral loading dose of divalproex sodium of 15 mg/kg/day is well tolerated and allows rapid achievement of therapeutic valproate levels in pediatric psychiatric patients aged 6 to 12 years. With this dose, therapeutic levels were achieved in most patients (81.3%) by day 5 of treatment. Overweight children receiving doses calculated based on TBW were significantly more likely to reach supratherapeutic valproate levels and experience a higher incidence of adverse effects, particularly sedation. In contrast, dosing based on AjBW (IBW + 40% of excess weight) resulted in more appropriate serum levels, fewer adverse effects, and better tolerability. The mechanism by which excess body weight influences drug levels remains unclear; an increased Vd in adipose tissue has been hypothesized but not confirmed.

Phenytoin

Phenytoin has an oral absorption of 80%, with high plasma protein binding of 92.2% and a Vd of 0.95 L/kg. It undergoes hepatic metabolism via CYP2C9 and CYP2C19 enzymes, a process that is inducible and saturable, with a t_1/2_ of 10–15 h.

Regarding PFH, two studies were identified referring to fosphenytoin, a prodrug of PFH. Messinger et al. [84] evaluated whether BMI influences serum PFH concentrations after a fosphenytoin loading dose in pediatric patients. They concluded that obesity does not significantly affect serum PFH concentrations in children receiving a weight-based fosphenytoin loading dose. No adjustments in fosphenytoin loading dose are required in obese pediatric patients, in contrast to findings in adults.

Prusakov et al. [85] conducted a study to determine whether there are significant differences in Vd between obese and non-obese children receiving an IV fosphenytoin loading dose, concluding that Vd is similar between obese and non-obese children. Therefore, adjustment of the fosphenytoin loading dose based on obesity status in pediatric patients is not justified. These findings contrast with studies in adults, where an increased Vd has been observed in obese patients

#### 3.3.11. Anesthetic Agents

Ketamine

Ketamine is a dissociative anesthetic that shows high IM F (90%), moderate protein binding (47%), a Vd of approximately 2–3 L/kg, and a half-life of about 2.5 h. It is extensively metabolized hepatically to the active metabolite norketamine, mainly via CYP3A4 and CYP2B6. Pediatric patients have higher Cl and faster metabolism, resulting in somewhat different PK compared to adults, with faster onset and shorter drug residence times.

One study in obese pediatric patients with ketamine was identified in this review, as summarized in Table 9. Street and Gerard [86], conducted a prospective observational cohort study including 43 patients to evaluate a fixed-dose IV ketamine protocol and to compare its use in normal-weight versus overweight/obese patients. A standardized regimen was applied consisting of an initial 50 mg IV bolus administered over 30–60 s, followed by 25 mg IV boluses as needed to achieve and maintain a Ramsay Sedation Score (RSS) ≥ 5.

With the initial 50 mg dose, RSS ≥ 5 was achieved in 35/43 patients (81.4%), with the remaining patients achieving this after the first additional 25 mg bolus. The total dose was higher in absolute terms in patients with obesity (median 100 mg vs. 75 mg), but when normalized by TBW, obese patients received lower doses per kg (median 1.16 mg/kg vs. 1.31 mg/kg, *p* = 0.01). When recalculated based on IBW, both groups received similar doses per kg (1.35 vs. 1.31 mg/kg; *p* = 0.67), suggesting that the fixed-dose protocol may approximate IBW-based dosing in patients with excess body weight.

These findings indicate that a fixed-dose regimen with titration to effect can achieve adequate sedation in most patients regardless of BMI. However, as the evidence is based on a single study conducted in a specific clinical setting, these results should be interpreted with caution and do not allow for definitive conclusions regarding optimal dosing strategies in obese pediatric patients.

Propofol

Propofol is a hypnotic-sedative administered exclusively via the IV route due to its very low oral F and high hepatic extraction (>90%). It exhibits high plasma protein binding (97–99%), a large Vd that is higher in children (5–11 L/kg) than in adults (3–6 L/kg), primary hepatic metabolism via conjugation, and a variable t_1/2_ that is shorter in children (3.5 h) than in adults (up to 12 h). Pediatric patients clear propofol faster and display distinct Pk profiles requiring careful dosing considerations.

Eight studies in obese pediatric patients receiving propofol were identified in this review, as summarized in Table 9. The available evidence includes both empirical PK/clinical data and model-based approaches, which are described separately. Olutoye et al. [87] showed that the dose required to induce loss of consciousness in 95% of patients (ED95) was significantly lower in obese children than in normal-weight children (2.0 vs. 3.2 mg/kg TBW). Standard TBW-based dosing (2.5–3.5 mg/kg) may cause exaggerated responses in obese patients; therefore, a reduced induction dose of 2.0 mg/kg TBW is recommended; however, as these results derive from a specific study, they should be interpreted cautiously and do not allow for definitive dosing recommendations.

Chidambaran et al. [88] conducted an observational study (2013) evaluating total intravenous anesthesia with propofol in adolescents with obesity, assessing anesthetic depth, plasma concentrations, emergence, and respiratory events. The observational study reported deeper-than-target anesthesia and delayed recovery, suggesting potential overexposure in this population.

Rogerson et al. [89] analyzed 1976 patients (2–21 years) with cancer to assess the impact of BMI on propofol sedation. Overweight and obese children received lower doses per kg of TBW to achieve adequate sedation compared to normal-weight patients, whereas underweight patients showed a higher rate of adverse events (10.6% vs. 3.5%), mainly hypoxia and apnea. These findings highlight variability in dose requirements across BMI groups, although the observational nature of the study limits the ability to establish causal relationships or define optimal dosing strategies.

Sahinovic et al. [90] reviewed recent advances in propofol PK/PD and supported the use of unified models such as that of Eleveld et al. They highlighted that V1 does not increase linearly with weight but follows a sigmoidal Emax function, reaching a plateau. This behavior may help explain differences in dose requirements observed across weight groups. TBW was identified as the best descriptor of Vd, whereas FFM better predicts Cl, suggesting that different body size descriptors may be relevant for different PK parameters. The review also noted that Cl in neonates and young children is limited by enzymatic immaturity, incorporated in the Eleveld model through a maturation function, and emphasized the risk of propofol infusion syndrome (PRIS) with prolonged exposure or high doses, reinforcing the need for cautious dosing and close monitoring in high-risk pediatric patients.

Four PopPK/PBPK modeling studies were identified. Diepstraten et al. (2012) [91] addressed, through a prospective PK study, the lack of dosing guidance for propofol in children and adolescents with morbid obesity. They found that TBW was the most predictive covariate for propofol CL outperforming IBW and LBW; accordingly, maintenance anesthesia dosing appeared to be better described using actual body weight with allometric scaling. The estimated exponent (0.8) indicates that elimination capacity increases with body weight in a non-linear manner, supporting the avoidance of simple mg/kg TBW dosing schemes. Subsequently, in 2013, the same authors [92] expanded this framework through a population PK meta-analysis including patients with obesity across a wide age range, again showing that clearance increased allometrically with TBW. The study also identified age as an independent contributor to variability in clearance, reinforcing that, in pediatric populations—particularly at extremes of body size—maintenance dosing should take into account both body size and age. Although these findings provide a mechanistically plausible basis for dose individualization, their translation into routine clinical practice should still consider the limited availability of prospective outcome-based validation.

Eleveld et al. [93] developed a PK model designed to be robust across populations. They found that Cl scales allometrically with body weight (exponent 0.75) and that Vd1 stabilizes beyond 30 kg. These findings suggest that increases in central Vd do not follow a linear relationship with body weight, particularly in larger individuals. Despite initial front-end kinetic overprediction, the model showed better predictive performance than several specialized models and was proposed as a strategy may contribute to improved safety in target-controlled infusion (TCI) systems across pediatric, adult, and obese patients. However, as these conclusions are derived from model-based analyses, their applicability in obese pediatric populations should be interpreted with caution.

Chidambaran et al. [94] conducted a PK/PD modeling simulation study (2015). The authors explored a dosing regimen consisting of an induction dose of 1.4 mg/kg based on adjusted body weight (AjBW) and maintenance with progressively decreasing infusions using allometric scaling of TBW (exponent 0.75). While these simulations provide insight into potential dosing strategies, they are based on model-derived assumptions and should not be interpreted as definitive clinical recommendations. The authors also emphasized the potential role of bispectral index (BIS) monitoring to guide titration and reduce the risk of adverse events.

#### 3.3.12. Non-Opioid Analgesics

Acetaminophen

Acetaminophen has high oral F (85–98%), a Vd in pediatrics of 0.7–1.2 L/kg, and low plasma protein binding (10–25%). Metabolism is predominantly hepatic via conjugation (glucuronidation and sulfation), while a smaller fraction is oxidized via CYP2E1 to form N-acetyl-p-benzoquinone imine (NAPQI), which is detoxified by glutathione under normal conditions. Elimination is mainly renal as conjugated metabolites, with a half-life of 1.5–4.2 h in children and 2.9 h in adolescents.

Three studies evaluating acetaminophen in obese pediatric patients were included (Table 10 summarizes the included studies). Zempsky et al. [95] described the limited availability of robust clinical dosing data in his observational review, and identified PK modeling and simulation as tools used to address this gap. PBPK models showed agreement with observed adult data, although extrapolation to pediatric populations was noted as a limitation. Clinically, this study also reported the potential risk of underexposure with standard dosing, while noting that dose escalation may increase the risk of hepatotoxicity.

The following two studies are based on PK modeling and should therefore be considered exploratory and require prospective validation before informing clinical practice. Hakim et al. [96] used a two-compartment model with NONMEM to characterize acetaminophen PK in severely obese adolescents. Their model incorporated allometric scaling and identified TBW as the best predictor of Cl, while NFM, with a high contribution of fat mass, more accurately described Vd. Model-based simulations suggested higher doses may be required in obese adolescents to achieve exposure comparable to that in normal-weight individuals. Similarly, Anderson and Cortinez [97] applied PBPK and PK models, including an Emax relationship, to define target concentrations associated with analgesic effect. Their approach indicated that the contribution of fat mass to Cl is relatively high for acetaminophen and supported the use of TBW with allometric scaling for maintenance dosing.

Taken together, the included studies describe a nonlinear relationship between body weight and acetaminophen PKs in obesity, with evidence derived predominantly from model-based analyses and limited clinical observations.

#### 3.3.13. Opioid Analgesics

Fentanyl

Fentanyl has an intranasal F of 20%, buccal F of 65%, and sublingual F of 54%, with 80–85% binding to alpha-1-acid glycoprotein. It is highly lipophilic, with redistribution into muscle and adipose tissue, and has a Vd in pediatrics of 5–30 L/kg (mean 15 L/kg) and 4–6 L/kg in adults. Distribution is multicompartmental, with predominant hepatic metabolism via CYP3A4 to inactive metabolites, and elimination occurs almost exclusively by biotransformation. The t_1/2_ in pediatric patients aged 6 months to 14 years (after long-term continuous infusion) is 11–36 h, and 2–4 h in adults; when administered as a continuous infusion, the half-life is prolonged with infusion duration due to the large Vd.

Six studies evaluating fentanyl using different PK models were included. (Table 10 summarizes the included studies). The first three studies are based on empirical PK data derived from observational and clinical settings. Among these, Okada et al. [98] evaluated the PK of a single intravenous dose of fentanyl (1 µg/kg TBW), observing significantly higher plasma concentrations in obese children at 5 min (0.88 vs. 0.53 ng/mL; *p* = 0.01). The model identified FFM as the best predictor of central Vd, and model-based simulations suggested peaks 60% higher when dosing was based on TBW compared to FFM. Johnson et al. [99], in a retrospective study in critically ill patients, observed high variability in response to fentanyl infusion. In non-obese children, higher initial doses reduced the probability of achieving target sedation (−19% per 10 µg/h), whereas in obese patients no significant association between initial dose and response was observed. Vaughns et al. [100] analyzed adolescents with severe obesity undergoing bariatric surgery, observing an increase in absolute Cl. The study used IBW-based dosing for induction (1–2 µg/kg), with PK parameters suggesting a higher risk of accumulation if TBW is used for maintenance dosing.

The following three studies are based on PK modeling and should therefore be considered exploratory and require prospective validation before informing clinical practice. Among these, Maharaj et al. [101] conducted a population PK model in obese children receiving continuous fentanyl infusion, observing that weight-normalized Cl decreases as the degree of obesity increases. Model-based simulations suggested that fixed dosing of 1 µg/kg/h based on TBW was associated with a higher probability of supratherapeutic concentrations (>3 ng/mL) with increasing body weight (15% at 16 kg vs. 43% at 164 kg), supporting the use of an allometric dosing approach. Lim et al. [102], through simulations in 4376 patients using a semi-physiological model, observed that absolute Cl is slightly higher in obese patients > 6 years, but weight-normalized Cl decreases by 11–30%. The steady-state Vd was significantly higher in obese patients, with model-predicted steady-state concentrations 25–77% higher and a t_1/2_ 2–4 times longer when dosing was based on TBW.

Gerhart et al. [103] developed a PBPK model for fentanyl and methadone. For fentanyl, the model predicted that absolute Cl increased, but weight-normalized Cl decreased in obesity, and model-based simulations suggested higher steady-state concentrations with TBW-based dosing, supporting dose adjustments according to age and obesity status. Regarding methadone PK, it is characterized by a high Vd (1–8 L/kg), high protein binding, and complex hepatic metabolism mainly mediated by CYP2B6, with formation of inactive metabolites and a prolonged and highly variable t_1/2_ (8–59 h). This information is primarily derived from model-based analyses. The model highlighted that exposure was primarily determined by CYP2B6 genotype, with a potentially higher risk of overexposure in slow metabolizers.

We acknowledge that a proportion of these studies are based on PK modeling approaches; therefore, the empirical evidence supporting these findings may be limited and heterogeneous.

Morphine

Morphine has an F < 40%, with plasma protein binding of 20–35%. In contrast to fentanyl, it is more hydrophilic, with a lower Vd of 1–4.7 L/kg for the IV route, and undergoes hepatic metabolism mainly via glucuronidation (UGT2B7) to active metabolites such as morphine 6-glucuronide (M6G) and inactive or potentially antagonistic metabolites such as morphine 3-glucuronide (M3G). Elimination is predominantly renal, with an initial t_1/2_ in pediatrics of 1–2 h and a mean terminal t_1/2_ of 18.6 h.

Among the studies on opioid analgesics, only one article on morphine met the inclusion criteria (Table 8 summarizes the included study). Dalesio et al. [104] evaluated the impact of obesity and obstructive sleep apnea (OSA) on morphine PK in pediatric patients. Following administration of a single dose of 0.05 mg/kg based on IBW, patients with obesity and OSA showed higher peak concentrations and a lower Vd compared to controls, with no relevant differences in Cl. In addition, an increased M3G/morphine ratio and higher M3G concentrations were observed, suggesting faster metabolism toward inactive metabolites. It was concluded that the combination of obesity and OSA enhances morphine metabolism, and these patients may require lower doses with more frequent administration. Despite dosing based on IBW, obese patients with OSA exhibited higher plasma exposure, which may increase the risk of adverse effects.

Finally, one study investigated opioid-based sedation in obese pediatric patients undergoing mechanical ventilation. Ward et al. [105] in a secondary analysis of a multicenter trial, evaluated sedative and opioid use in this population, although weight-based dosing TBW resulted in similar doses per kilogram, obese patients received significantly higher total and cumulative opioid exposure (e.g., fentanyl 7553 vs. 5496 μg; morphine 417.6 vs. 238.1 mg). Obese patients also experienced longer opioid exposure (12 vs. 9 days) and higher rates of inadequate sedation (37% vs. 24%), without differences in withdrawal. In terms of clinical outcomes, obesity was associated with prolonged mechanical ventilation, longer PICU stay, and higher 28-day mortality, particularly under protocolized sedation. Overall, TBW-based dosing was associated with increased drug exposure and poorer clinical outcomes in obese pediatric patients.

#### 3.3.14. Benzodiazepines

Midazolam

Midazolam has an oral F of 36% and a sublingual F of 50%, with 97% plasma protein binding, mainly to albumin, and a Vd in pediatrics of 1.24–2.02 L/kg. It undergoes mainly hepatic metabolism as a CYP3A4 substrate, with a t_1/2_ of 3–4.5 h for the IV route.

All three identified studies were based on PK modeling approaches and should therefore be interpreted in light of their limitations, particularly in the absence of direct clinical validation, of which two [106,107] evaluating PK in obese patients older than 12 years were included. Both showed that body weight increases Vd, delaying the time to reach steady state after IV infusion and increasing the risk of accumulation and higher exposure during prolonged infusions. For IV midazolam dose adjustment in obese adolescents, IBW or LBW should be considered. Gade et al. [107], through model-based simulations after fixed buccal administration, observed lower and shorter-lasting concentrations in obese patients, which may result in subtherapeutic levels.

A recent study, conducted by McCann et al. [108], was an observational multicenter study using real-world data to evaluate midazolam PK in children with and without obesity. A two-compartment PopPK model estimated Cl of 14.9 L/h/70 kg with high interindividual variability (185%), and no significant covariate effects beyond TBW. PBPK simulations suggested slightly higher exposure in obese children, with increases in AUC and steady-state concentrations generally <20% and Cmax differences < 10%. Weight-based dosing resulted in 14% higher exposure in obesity, whereas IBW-based dosing led to underexposure (30% lower). Overall, obesity appeared to have a limited impact on midazolam PK, suggesting that dose adjustment may not be necessary for initial dosing. Nevertheless, due to the high interindividual variability that persists despite weight-based scaling, clinical caution and close monitoring are required. The included studies are summarized in Table 11.

Diazepam

Diazepam has an oral F greater than 90% and a rectal F of 90%, with 95–99% plasma protein binding, and a Vd of 0.8–1.46 L/kg. It undergoes mainly hepatic metabolism as a CYP3A4 and CYP2C19 substrate, with a t_1/2_ 15–21 h in children aged 2–12 years and 18–20 h in adolescent, for the IV route.

Only one study on diazepam was identified, conducted by McCann et al. [109], a multicenter observational study using real-world data to evaluate diazepam PK in children with and without obesity. A two-compartment PopPK model with allometric scaling estimated Cl of 2.29 L/h/70 kg and a large central Vd (58.2 L/70 kg) with high interindividual variability. Obesity did not appear to significantly affect PK beyond TBW, although a slight increase in Vd was observed. Model-based simulations suggest that standard dosing (0.2 mg/kg capped at 8 mg) is associated with higher probability of underexposure in older children with obesity, failing to achieve target concentrations (<200 ng/mL at 10 min). Removal of the dose cap appeared to improve target attainment, with doses up to 32 mg required in some cases. Overall, dose capping, rather than obesity itself, may have contributed to underexposure. These findings are based on PK modeling studies, and their interpretation should take into account the nature of the underlying evidence. Table 11 summarizes the included studies.

#### 3.3.15. Hypnotics and Sedatives

Dexmedetomidine

Dexmedetomidine has good F for intranasal, SC, and sublingual routes, with 94% plasma protein binding and a steady-state Vd in adults of 1.7–2.2 L/kg; in children aged 1–9 years, central Vd is 84.3 L and peripheral Vd is 114 L. It undergoes metabolism via direct glucuronidation (UGT1A4 and UGT2B10), aliphatic hydroxylation by CYP2A6, and N-methylation by CYP2D6, generating inactive metabolites. The elimination half-life is approximately 4.4 h in children aged 1–9 years and 2–2.8 h in adults.

Three studies in obese pediatric patients receiving dexmedetomidine were identified and are summarized in Table 11. These include one empirical study and two model-based studies (PopPK/modeling approaches).

Only one empirical clinical study was identified. Wu et al. [110] conducted a prospective experimental dose-finding study in pediatric patients with and without obesity undergoing elective orthopedic surgery. Sequential IV bolus doses (0.3–0.8 µg/kg TBW) were administered using a biased coin design. The ED95 was similar in both groups: 0.75 µg/kg (95% CI 0.64–0.78) in obese patients and 0.74 µg/kg (95% CI 0.60–0.78) in normal-weight patients, suggesting that dose adjustment may not be required for a single slowly administered bolus when based on TBW in this specific setting. However, this finding is limited to single-dose administration with early sedation assessment and should not be extrapolated directly to maintenance infusions, prolonged sedation, or other clinical contexts.

Two PopPK/PBPK modeling studies were identified. Morse et al. [111] developed a universal PopPK model of dexmedetomidine using pooled pediatric and adult data (3.1–152 kg), including obese adults. A three-compartment model with first-order elimination best described the data, incorporating allometric scaling for body size and a maturation function for age. In this model, obesity was best characterized using FFM as the descriptor for clearance and NFM for volumes of distribution. These findings suggest that TBW-based maintenance dosing may not fully account for PK variability in patients with obesity. However, as these conclusions are derived from population PK modeling, they should be interpreted with caution.

Morse et al. (2021) [112] developed a narrative review that integrated previously published population PK models and PK/PD simulations to optimize TCI systems in pediatrics. Simulations based on a three-compartment model suggested that dosing could be informed by the volume at peak effect (VPe), with infusions over 10–20 min potentially reducing adverse effects such as hypertension and bradycardia. The model incorporated FFM and NFM to adjust Cl and Vd, indicating that integration of maturation, body size, and fat mass may improve the characterization of dexmedetomidine PK. Nevertheless, these findings are derived from PK simulations and should be considered hypothesis-generating rather than definitive guidance for clinical practice.

The available evidence for dexmedetomidine in pediatric patients with obesity remains limited. PopPK model-based studies provide useful information on the potential influence of body composition on clearance and distribution but require specific clinical validation. Direct clinical evidence suggests that a single TBW-based bolus may be appropriate in selected settings, whereas uncertainty persists for prolonged infusions, maintenance dosing, and severe obesity. Therefore, dosing conclusions should remain cautious and should clearly distinguish direct empirical evidence from model-derived findings.

#### 3.3.16. Immunosuppressants

Cyclosporine

Modified cyclosporine has an F in children of 43%, with protein binding of 90–98% to lipoproteins. It is widely distributed, including in the liver, pancreas, and lungs, with a Vd of 4–6 L/kg. It undergoes extensive metabolism via CYP3A4 and P-glycoprotein, with biphasic elimination and a terminal t_1/2_ of 8.4 h (range: 5–18 h). Excretion is mainly biliary, with a minor renal contribution of 6%.

Among the studies on immunosuppressants, only one article on cyclosporine met the inclusion criteria. Kasap et al. [113] conducted a retrospective observational study in pediatric renal transplant patients, evaluating the impact of obesity on cyclosporine dosing. Overweight and obese patients required significantly lower cyclosporine doses, regardless of the body size descriptor used (body weight, BMI, or BSA), to achieve comparable blood concentrations to normal-weight patients. These findings suggest that dosing based solely on TBW may lead to overexposure and increase the risk of toxicity in this population. Table 11 summarizes the included studies.

#### 3.3.17. Electrolytes

Magnesium sulfate

Only one study evaluating magnesium sulfate was identified. This study assessed whether a simplified high-dose magnesium sulfate infusion regimen is as safe and effective as a previously used more complex regimen for treating children with status asthmaticus unresponsive to standard initial therapy. In obese patients, dosing was based IBW, which can be explained by the PK profile of magnesium sulfate as a highly hydrophilic compound that distributes primarily within the extracellular fluid compartment, with minimal penetration into adipose tissue; consequently, its Vd does not increase proportionally with TBW. In parallel, although renal Cl may be modestly increased due to obesity-related changes in glomerular filtration, this does not occur in a linear relationship with body weight, further supporting the use of IBW-based dosing. Serum magnesium levels were measured before and after infusion, showing no statistically significant differences between study groups, and no clinically relevant adverse effects were reported [114]. Table 11 summarizes the included studies.

## 4. Discussion

Our review highlights the limited availability of information on the dosing of HAM in obese pediatric patients. Data were identified for only 27 of the 100 drugs considered, highlighting a significant knowledge gap. This is particularly relevant in a clinical context where the prevalence of childhood obesity continues to increase. Given the associated comorbidities and potential risks, optimizing pharmacotherapy in this population is essential.

In addition, the overall quality of the available evidence is generally low. Most of the studies analyzed were observational and retrospective, with fewer than 30 patients included in the obese group. This substantially limits the robustness of the findings and their clinical applicability.

A further methodological concern relates to the interpretation of model-informed approaches, such as PopPK and PBPK models, within the context of this limited evidence base. These approaches are inherently dependent on the quality, consistency, and representativeness of the underlying data, which in the field of pediatric obesity remain sparse, heterogeneous, and fragmented across drugs and age groups.

Consequently, while model-based analyses may provide valuable exploratory insights, they may also create an impression of precision that is not fully supported by robust empirical evidence. To avoid misinterpretation and unwarranted clinical extrapolation, these findings must be explicitly defined as provisional, non-definitive, and unsuitable for guiding clinical decision-making. This distinction is particularly critical in the context of HAM, where dosing inaccuracies may have significant safety implications.

In this context, model-informed approaches such as PopPK and PBPK models may help to interpret the variability observed across studies and pharmacological groups, particularly when empirical data are sparse or inconsistent. These models can provide a mechanistic framework to explore how physiological changes associated with obesity—such as alterations in body composition, organ size, and clearance pathways—may influence drug exposure.

However, underscoring their exploratory nature, their interpretative value is constrained by the quality and representativeness of the underlying data, and therefore their use in this review is limited to supporting the understanding of observed trends rather than establishing definitive dosing recommendations. Notably, direct empirical evidence and model-based inferences should not be regarded as providing equivalent levels of support for clinical dosing recommendations. Prospective PK studies with appropriate validation remain a mandatory prerequisite before any model-derived strategy can be translated into routine clinical practice.

From a clinical perspective, the following sections summarize practical dosing considerations derived from the available evidence, highlighting preferred approaches and situations where caution is warranted.

### 4.1. Antidiabetic Agents

Obesity in pediatric patients with T1DM is associated with insulin resistance, which has important implications for dosing. For insulin dosing, TBW appears to be the most appropriate descriptor for both treatment initiation and dose adjustment, whereas the use of IBW may underestimate requirements in obese patients [24]. Although no differences are observed in doses expressed as IU/kg/day between normal-weight and obese patients, dosing adjusted by BSA reveals higher requirements in overweight and obese patients, suggesting that this descriptor may better reflect underlying insulin resistance [26]. Consistently, insulin resistance increases during puberty. However, under conditions of optimal glycemic control, weight-adjusted insulin requirements do not differ between groups, suggesting that intensive titration may partially compensate for these alterations [25].

The available evidence indicates that TBW is the recommended descriptor for the initial loading dose, adjusted according to age, pubertal status, and disease severity. For maintenance therapy, some recommendations suggest the use of IBW followed by dose titration based on clinical response, adopting a conservative strategy to minimize the risk of hypoglycemia, with subsequent titration based on clinical response [13,115].

However, unlike other drug classes, insulin dosing in this population is not guided by PK parameters, nor by drug lipophilicity. Instead, dosing strategies rely on empirical, clinically driven approaches based on body size descriptors and real-time glycemic response, reflecting the predominant role of pharmacodynamic factors—particularly insulin resistance—over classical PK determinants.

Importantly, the available evidence is derived exclusively from observational studies, without the PBPK modeling. Therefore, current recommendations are supported by direct clinical evidence rather than mechanistic simulations. In this framework, dosing follows a reactive approach in which initial calculations based on TBW or BSA are subsequently titrated according to glycemic control.

Available evidence on semaglutide in pediatric obesity remains limited and heterogeneous. A retrospective observational study by van Boxel et al. [27] demonstrated significant reductions in BMI and body weight, concluding that semaglutide appears to be a safe and effective adjunct for weight management within a multidisciplinary setting, although gastrointestinal AEs and treatment discontinuations were reported. In parallel, PBPK modeling studies [28] suggest that fixed dosing may not be optimal, as younger and lower-weight children may experience higher systemic exposure, increasing the risk of AEs, while heavier patients may be underexposed. Despite the limitations inherent to its model-based design, these findings may indicate the need for individualized dosing strategies in pediatric patients with obesity, as PK variability may not be fully captured by current approaches. However, it is important maintain clinical monitoring, until further robust validation is available.

Regarding liraglutide, several studies [30,31,34] have shown that body weight is the main body size descriptor for drug exposure. A systematic review [33] highlighted that, due to the frequency of AEs—mainly gastrointestinal—dose escalation should be performed weekly, starting at 0.3 mg/day in children aged 7–11 years, with increments of 0.3 mg up to 1.2 mg and subsequently increases of 0.6 mg. However, a meta-analysis in adolescents with obesity [31] indicated that the maintenance dose should be 3 mg without weight adjustment. This finding is consistent with another review [116], which reported that drug exposure in adolescents is similar to that in adults. Furthermore, a prospective double-blind experimental study [29] recommends caution in children weighing <45 kg, in whom a maximum dose of 2.4 mg weekly may be considered.

Available evidence, although limited, suggests that for glucagon-like peptide-1 (GLP-1) receptor agonists such as liraglutide and semaglutide, a strictly TBW-based dosing approach may not be appropriate. While body weight appears to influence systemic exposure, this does not reflect a proportional increase in distribution or Cl with increasing adiposity. Their high protein binding, limited-to-moderate Vd, and predominantly proteolytic elimination indicate that excess adipose tissue likely has a minimal impact on distribution, supporting a cautious approach to TBW-based dose escalation.

Metformin is an oral antidiabetic agent extensively studied in pediatric patients. The majority of studies reviewed suggest that TBW is the main determinant of metformin Cl and Vd, with Cl in obese adolescents being higher than in non-obese individuals and comparable to that observed in adults. However, several authors [35,41] reported that dosing in obese pediatric patients is often based on fixed age-related regimens rather strictly weight-based approaches.

Evidence supporting these dosing strategies is predominantly based on empirical clinical data, with only a minor contribution from PK modeling approaches. In this context, findings from PK modeling [36] and prospective studies [35] suggest that adult dosing might be considered in cases where pediatric regimens are ineffective. This approach is broadly consistent with current guidelines, which recommend dose escalation in line with TBW to compensate for increased Cl [117], and suggest that, as Cl in obese children approaches that of healthy adults, the conventional upper limit of 2000 mg may be reconsidered, allowing adult maximum doses (2550–3000 mg) when clinical response is inadequate [10,12].

An important consideration is the delay of approximately 4–8 weeks before a clinically meaningful reduction in BMI is observed. This should be communicated to families, as adherence has been identified as the most important determinant of successful weight loss, with maximal effect typically achieved between 6 and 9 months of treatment, after which BMI tends to stabilize [37].

Metformin dosing should be interpreted in light of its hydrophilic profile, minimal protein binding, extensive distribution into lean tissues, and primarily renal elimination. As it does not substantially distribute into adipose tissue, a strictly TBW-based dosing approach may not be mechanistically appropriate. Available data suggest that higher body weight is associated with increased apparent Cl and reduced exposure, likely reflecting obesity-related renal changes rather than proportional scaling with TBW. Therefore, higher or adult-like doses may be considered when the clinical response is insufficient, although dose adjustments should be made cautiously and guided by renal function and tolerability rather than linear TBW-based escalation.

### 4.2. Oral Anticoagulants

Within the group of oral anticoagulants, only one observational study focused on warfarin was identified. The authors reported that obese patients require lower doses per TBW than normal-weight children and suggested that dose capping should be avoided to prevent delays in achieving a therapeutic INR. In addition, a review by Kendrick et al. [118] recommends initial dosing based on TBW with close monitoring of coagulation parameters. Additionally, it is worth noting that warfarin is a drug with relative lipophilicity, high plasma protein binding, and metabolism mediated by the CYP2C9 enzyme, whose activity has been reported to be increased in obese patients [7,119]; therefore, the use of IBW may a priori lead to situations of underdosing.

### 4.3. Heparin and Other Antithrombotic Agents

LMWH are hydrophilic drugs with limited distribution into adipose tissue. According to the studies reviewed, dosing based on TBW in obese pediatric patients results in higher exposure, as reflected by anti-Xa levels, and may increase the risk of bleeding. For prophylaxis, BMI-based dosing has been standardized with favorable outcomes [49,50], and two retrospective observational studies have proposed dose reduction strategies to avoid supratherapeutic anti-Xa levels [52,53]. In addition, three population PK model-based studies—one in prophylaxis and treatment [54], and two in treatment [55,56]—suggest that FFM may be an appropriate dosing descriptor; however, these findings are derived exclusively from modeling approaches and should therefore be considered exploratory, requiring prospective validation given the limited supporting empirical evidence. These findings are generally in line with dosing guidelines that recommended the use of AjBW with a correction factor of 0.35–0.4 [13,118,120]. Nevertheless, due to the marked variability in response, TDM using anti-Xa activity is essential to guide dose adjustments and ensure target exposure for both prophylaxis and treatment. Clinically, these findings support avoiding exclusive TBW-based dosing for LMWH in obese pediatric patients due to the risk of overexposure, favoring FFM- or AjBW-based approaches combined with anti-Xa monitoring. This behavior is explained by their hydrophilic nature and confinement to the intravascular and extracellular compartments, which do not expand proportionally with adipose tissue in obesity.

Studies on UFH are mainly retrospective observational studies with small sample sizes and without multivariate analyses, limiting the strength of the evidence. However, based on the available data, UFH dosing in obese children may require adjustment to achieve the desired therapeutic targets. Given its hydrophilic nature, some authors recommend dosing based on AjBW with a correction factor of 0.4 [12,13,120], whereas others advocate TBW for both the initial bolus and maintenance dosing [118,121]. Nevertheless, TBW-based dosing may lead to supratherapeutic anticoagulation due to limited distribution into adipose tissue and non-linear elimination pathways, while variability in protein binding and reticuloendothelial clearance further underscores the need for individualized, anti-Xa-guided dosing.

### 4.4. Antihypertensive Agents

ANGI and CCB represent key therapeutic options for hypertension associated with pediatric obesity. For ANGI and ARB, such as ramipril or valsartan, available studies suggest that obese children exhibit a similar antihypertensive response to their normal-weight counterparts at equivalent doses when dosing is based on BSA [65,122]. Some authors propose TBW-based dosing, starting with low doses and titrating according to response; for ramipril, a proposed regimen is 0.05–0.15 mg/kg/day (maximum 40 mg), based on TBW [116].

In contrast, CCB such as amlodipine and nifedipine have shown a reduced systolic response in obese pediatric patients [65,118,122]. This lower efficacy may be explained by an increased Vd in peripheral compartment due to excess adipose mass, we should be aware that these data are based on PK modeling with limited empirical evidence [66]. Consequently, these patients often require higher doses or combination therapy to achieve optimal blood pressure control. These findings are broadly aligned with a narrative review that considers TBW as a possible preferred dosing descriptor [116]. These findings can be explained by the high lipophilicity of these drugs and increased hepatic clearance associated with liver enlargement and enhanced hepatic blood flow in pediatric obesity.

### 4.5. Neuromuscular Blocking Agents

Within the group of neuromuscular blocking agents, only one study with succinylcholine was identified, in which neuromuscular blockade was associated with TBW. This observation appears to be in line with two reviews suggesting TBW-based dosing, supported by rapid clearance mediated by plasma cholinesterase and by the expansion of extracellular fluid volume in obese patients [118,123]. However, other authors suggest that, in critical situations, AjBW with a correction factor of 0.8 may be considered [13].

### 4.6. Antibiotics

Dosing of aminoglycosides in obese patients is complex due to their hydrophilic nature and narrow therapeutic index. Only two studies on gentamicin were identified, including one retrospective observational study and one based on a PopPK modeling approach. In addition, one study on tobramycin identified through cross-referencing, despite being published prior to the predefined review period, was also considered [124]. This study evaluated the Pk of tobramycin (2 mg/kg) in five obese children aged 1–9 years to estimate Vd. The results showed a significant reduction in Vd/TBW for tobramycin (197 ± 26 mL/kg) compared with normal-weight children, leading to the conclusion that the loading dose should be adjusted based on IBW plus extracellular fluid. Overall, findings for both gentamicin and tobramycin suggest that weight adjustment strategies, such as AjBW or FFM, may be required, consistent with recommendations from several authors [12,13,118,120]. In practice, TBW-based dosing may not be appropriate for aminoglycosides in obese children, and adjusted strategies such as AjBW or FFM are preferred to reduce the risk of toxicity. This is consistent with their low lipophilicity and limited penetration into adipose tissue, resulting in a smaller Vd relative to TBW and an increased risk of overexposure when TBW-based dosing is used.

The available evidence on vancomycin dosing in pediatric patients shows divergent findings depending on the body size descriptor used. A group of studies based on TBW [70,72,74] did not identify significant differences in initial trough concentrations between children with obesity and those with normal weight, suggesting that actual body weight may be an acceptable empirical basis for initial dosing. In contrast, larger studies [71,73] and meta-analyses [76] demonstrated that strict TBW-based dosing is associated with significantly higher trough concentrations in children with obesity. This finding was further supported by Khare et al. [75], who additionally highlighted that TBW is a reliable predictor of drug exposure as measured by AUC, whereas trough concentrations may be less informative. These findings likely reflect alterations in Cl and distribution associated with increased kidney size, renal blood flow, and hyperfiltration in obese children, along with variability in renal function, a major determinant of vancomycin elimination.

Given this variability, alternative PK-based approaches have been proposed; however, caution should be exercised when extrapolating these findings to clinical practice. Le et al. [77] recommended the use of allometrically scaled body weight to better estimate Cl, whereas Moffett et al. [78] suggested that in larger pediatric patients (≥70 kg), FFM may better predict vancomycin PK than TBW. Furthermore, Zhang et al. [80] reported increased Cl in obese adolescents, cautioning against direct extrapolation from adult dosing. In line with this, Smit et al. [79] proposed dosing strategies that integrate TBW with renal function to safely achieve the PD target of AUC/MIC. However, although TBW-based dosing is commonly applied, obesity-related increases in renal Cl and Vd are not fully proportional, leading to potential AUC/MIC overshooting; thus, renal function and TDM are critical to ensure target attainment while minimizing toxicity [10,12,13,116,120].

### 4.7. Antiepileptic Drugs

Five studies were identified within the antiepileptic group, data on valproic acid PK in the context of obesity are contradictory, with no consistent differences in exposure or steady-state serum concentrations across BMI categories reported. Some studies suggest a closer association with IBW or adjusted dosing strategies [81,83]. Conversely, other authors advocate for TBW-based dosing combined with TDM to individualize therapy and ensure target attainment [13,120,125]. This observation may be explained by its moderate lipophilicity and predominant glucuronidation-mediated Cl, a metabolic pathway that has been shown to be increased in obese patients, potentially offsetting changes in distribution [7,119].

PFH requires a differentiated approach between loading and maintenance dosing due to its distribution characteristics and saturable metabolism. Based on the studies included in this review [84,85], the loading dose in status epilepticus may be based on TBW, in line with treatment guidelines, as no relevant changes in Vd have been observed and adequate concentrations appear to be achieved [12,13,118]; this finding may be explained by its high plasma protein binding, which may attenuate the impact of increased adipose tissue on drug distribution despite its relative lipophilicity. On the other hand, obesity may be associated with increased CYP2C9/2C19-mediated metabolism [119,126], this does not translate into a proportional increase in clearance due to the saturable, nonlinear PK of phenytoin; therefore, AjBW with a correction factor of 0.3 is generally recommended for maintenance dosing [113,117,119]. Nevertheless, TDM remains essential for individualized dose adjustment, and plasma albumin levels should be considered, as they may influence the free fraction of PFH [13,14].

### 4.8. Anesthetic Agents

Ketamine is a lipophilic drug; however, the reviewed literature suggests that excess fat mass does not justify a proportional increase in the initial dose, likely because target organs and highly perfused tissues do not increase to the same extent as adipose tissue in obesity. Accordingly, clinical guidelines suggest that ketamine dosing in obese pediatric patients may not need to be strictly based on TBW, as this could increase the risk of overdose and toxicity [115,123]. In this context, the findings by Street et al. [86] support this observation, demonstrating that obese patients achieve adequate sedation with lower doses per kg of TBW than normal-weight patients, and that effective doses are closer to IBW-based dosing rather than TBW-based approaches [86,125]. Additionally, the study suggests that a fixed-dose regimen with titration to effect may represent a practical and safe approach in emergency settings [86,125].

However, as the available evidence is limited and derived from a small number of studies conducted in specific clinical settings, these findings should be interpreted with caution and do not allow for definitive conclusions regarding optimal dosing strategies in obese pediatric patients.

In contrast, for propofol, obesity-related physiological changes and its high lipophilicity influence both distribution and Cl, which has implications for dosing strategies. Propofol dosing should be interpreted by distinguishing between induction and maintenance phases. For induction, both clinical studies and guidelines suggest that unadjusted TBW-based dosing may be associated with an increased risk of overdosing, as Vd does not increase linearly with body weight, thereby increasing the risk of peri-induction hypotension and exaggerated PD response [13,87,90,93,127]. This reflects its lipophilicity and rapid redistribution to highly perfused tissues, while the increase in adipose tissue does not proportionally contribute to the initial PK effect.

In this context, the use of IBW or LBM, as well as a conservative approach of 2 mg/kg based on TBW, may represent to be safer approaches for the initial bolus [13,87,127]. Conversely, for maintenance infusion, the evidence appears to indicate that TBW may be an appropriate descriptor of Cl when applied using allometric scaling, since propofol elimination increases with body weight in a non-linear manner and the use of simple linear mg/kg/h regimens may lead to accumulation and overexposure [13,92,94,127,128]. Model-based approaches [91,92,93,94] further support these observations, indicating non-linear relationships between body size and PK parameters. Nevertheless, these conclusions are informed by a combination of empirical observations and model-based analyses, and simulation-based findings should therefore be interpreted with caution.

### 4.9. Analgesics

Pediatric obesity alters the PK of acetaminophen, as established by observational studies and narrative reviews showing that Cl increases in a non-proportional manner relative to TBW, thereby influencing the selection of the body size descriptor according to the treatment phase [95].

Evidence from PK modeling studies suggests that TBW is the most appropriate descriptor for the loading dose, as it reflects initial Vd, whereas for maintenance dosing, NFM combined with allometric scaling has been proposed to better account for the nonlinear increase in clearance [96,97]. These model-based approaches consistently identify TBW with allometric scaling as the main predictor of Cl and NFM as a more accurate descriptor of distribution, reflecting the partial contribution of adipose tissue. However, these recommendations are largely derived from simulations and lack robust clinical validation.

From a PK perspective, acetaminophen’s low protein binding and moderate lipophilicity support the use of TBW for the loading dose, while its predominantly hepatic metabolism explains the nonlinear scaling of Cl with body size. Increased CYP2E1 activity in obesity may also enhance NAPQI formation, introducing a safety concern not captured by PK models [7,119].

From a clinical perspective, the use of standard dosing without adjustment may lead to underexposure, whereas empirical dose escalation may increase the risk of toxicity [95]. Other authors recommend the use of AjBW with a correction factor of 0.35–0.4 for both loading and maintenance dosing, with the aim of minimizing hepatic adverse effects related to the formation of hepatotoxic metabolites such as NAPQI [13,116,120,129].

Integrating both empirical findings and model prediction suggest a phase-dependent dosing strategy: TBW may be used for the initial (loading) dose, while maintenance dosing should avoid simple linear TBW-based calculations and instead consider adjusted descriptors (e.g., AjBW) or nonlinear scaling approaches suggested by modeling. Given the predominance of model-based evidence over primary clinical data, any dose adjustment should be applied cautiously, with close clinical monitoring to balance efficacy and hepatotoxicity risk.

Regarding opioid analgesics, in obese pediatric patients, available evidence indicates that TBW is not a universally appropriate descriptor for opioid dosing [105], and a drug-specific approach is required. These differences are explained by heterogeneous PK profiles influenced by lipophilicity, metabolism, and the formation of active metabolites.

Evidence derived from clinical and observational PK studies indicates that opioid disposition in obese pediatric patients does not scale proportionally with total body weight. For fentanyl, clinical data suggest that dosing based on TBW may lead to higher peak concentrations, whereas lean body size descriptors such as FFM or IBW are associated with more predictable exposure. In parallel, although absolute Cl may increase, weight-normalized Cl decreases, reflecting that hepatic metabolism—primarily via CYP3A4—does not scale proportionally with excess adipose tissue [98,99,100].

These PK characteristics may help explain the phase-dependent dosing strategies observed across studies. For bolus dosing, FFM or IBW are preferred descriptors to reduce the risk of excessive peak concentrations and respiratory depression, whereas TBW-based dosing may lead to overexposure. For maintenance dosing, approaches based on nonlinear scaling of TBW, fixed dosing (µg/h), or lean body mass have been proposed to limit accumulation during continuous infusion.

Evidence from observational studies and clinical practice therefore supports the use of alternative body size descriptors such as FFM or IBW for initial dosing, along with reduced maintenance doses [99,100]. Current guidelines are consistent with these findings, recommending AjBW with a correction factor of 0.4 or IBW for loading doses [13,130,131], and LBW/FFM for maintenance dosing, given the stronger correlation between fentanyl Cl and lean body weight [10,118,123].

From a mechanistic perspective, PopPK and PBPK modeling studies have been used to further characterize fentanyl PK in obesity. In these models, fentanyl PK are typically described using multi-compartment approaches, with a Vd that increases more than proportionally with body weight. However, early distribution after a bolus is more closely related to lean mass, which explains why dosing based on TBW results in significantly higher peak concentrations compared to FFM-based dosing. As a high hepatic extraction drug, fentanyl Cl is closely linked to hepatic blood flow, further contributing to this nonlinear behavior. Model-based simulations further support that linear TBW dosing is associated with an increased probability of supratherapeutic concentrations as body weight increases [101,102].

Overall, while the available evidence consistently shows that TBW-based dosing increases the risk of overexposure in obese pediatric patients, many of these findings are derived from PK modeling and simulation studies. Therefore, dosing strategies should be applied cautiously, with close clinical monitoring to balance adequate analgesia and the risk of accumulation and prolonged effects.

For methadone, also a highly lipophilic drug, data on dosing in obese children are very limited. Available recommendations are primarily based on extrapolation from adult studies and expert consensus rather than robust empirical pediatric data. However, due to the risk of accumulation and toxicity, several sources recommend IBW or AjBW as the preferred descriptors for both loading and maintenance dosing [13,115,130]. In addition, a PBPK model developed by Gerhart et al. [103]. suggests that variability in drug exposure may be influenced by genetic factors, highlighting interindividual variability not captured by body size descriptors alone. These findings should be interpreted with caution, as they are based on a single PBPK study, and the consistency of the conclusions is therefore very limited.

Morphine is an opioid with higher hydrophilicity and a limited Vd that does not significantly increase with excess adipose tissue. Due to these properties, dosing based on TBW may overestimate the required dose, as the drug does not distribute extensively into adipose tissue, resulting in higher intravascular concentrations and an increased risk of toxicity. Given the increased risk of toxicity and respiratory depression associated with TBW-based dosing, which may overestimate dosing requirements, IBW or LBW are recommended as more appropriate body size descriptors [13,116,118,131]. This approach may be broadly aligned with current pediatric practice, where IBW-based dosing is used in obese children, although the available clinical evidence remains limited.

Careful dose titration according to analgesic response and continuous clinical monitoring of respiratory function are essential in these patients. In this context, IBW-based dosing should be considered a starting point rather than a definitive strategy, with subsequent dose adjustments guided by clinical response and safety parameters. Thus, IBW-based dosing is considered more appropriate to limit overexposure. In addition, clinical factors such as obstructive sleep apnea increase opioid sensitivity [104]. Given the observed variability, dose reduction and closer monitoring may be required in high-risk patients, particularly those with comorbid conditions affecting respiratory function.

### 4.10. Benzodiazepines

Midazolam is a lipophilic benzodiazepine, which promotes its distribution into adipose tissue and results in PK in obese adolescents that are better described by a two-compartment model [107,108]. Regarding the intravenous route, current evidence indicates that obesity primarily affects drug distribution rather than Cl what is consistent with the lipophilic nature. Gade et al. [107] demonstrated, in an exploratory microdose PK study, that BMI SDS significantly increases peripheral Vd. Similarly, the prospective PopPK study by van Rongen et al. [106] suggest that Cl remains unchanged, whereas peripheral volume increases proportionally to excess body weight. In line with this, McCann et al. [108], using combined PopPK and PBPK modeling with real-world data, confirmed that TBW is the most appropriate descriptor for scaling clearance, the robustness of this finding is constrained by the nature of the study and the considerable interindividual variability, which may limit its generalizability

Accordingly, several authors recommend using TBW for the loading dose or administering multiple “mini-loading” doses to avoid high central concentrations associated with apnea risk. For maintenance during continuous infusion, IBW-based dosing or fixed infusion rates (mg/h) may be considered, as Cl does not increase proportionally with body weight [13,118,120,131,132]. Close clinical monitoring of respiratory and cardiac function is essential, and dosing should be titrated according to individual therapeutic response. In addition, in cases of status epilepticus, buccal administration at fixed age-based doses may lead to subtherapeutic exposure [107,131]. These results are aligned with the PK profile of midazolam, a highly lipophilic drug, in which Vd increases in obese patients due to distribution into adipose tissue, although not proportionally to TBW, while clearance does not scale with excess body weight. This imbalance may prolong half-life and increase the risk of accumulation, supporting the use of TBW for loading doses and lean-based descriptors (IBW or FFM) for maintenance dosing, with careful clinical titration.

Regarding diazepam, findings from real-world PopPK modeling by McCann et al. [109] suggest that TBW, when combined with allometric scaling, may adequately describe Cl and Vd in pediatric patients with obesity. However, current dosing recommendations for status epilepticus, which include an 8 mg dose cap, were associated with subtherapeutic concentrations, and simulations indicated that higher weight-based doses (up to 32 mg) may be required to achieve target exposure. In line with several reviewed guidelines, TBW is generally recommended for loading doses, as it better reflects the expanded Vd associated with increased adipose tissue. In contrast, maintenance dosing may be more appropriately based on IBW, as Cl is primarily determined by hepatic metabolism and does not appear to scale proportionally with excess body weight. This distinction is particularly relevant for lipophilic drugs such as diazepam, in which accumulation in adipose tissue may prolong elimination and increase the risk of sustained or excessive pharmacological effects with repeated dosing [13,115].

It should be acknowledged that the studies on midazolam and diazepam are primarily based on population PK models; therefore, the available evidence is limited and should be interpreted with caution.

### 4.11. Hypnotics and Sedatives

For dexmedetomidine, obesity-related physiological changes, particularly the increase in adipose tissue, may influence drug distribution without a proportional increase in clearance, given that Cl is more closely related to lean body mass than to total body weight [13,112,130]. From a PK perspective, this may explain why TBW-based dosing for maintenance infusion could lead to overexposure in obese pediatric patients, whereas descriptors such as FFM may better reflect clearance-related processes [112].

However, although model-based analyses support FFM as the most appropriate descriptor for scaling clearance [111], in clinical practice, more pragmatic approaches such as IBW are often used, as they may represent a more feasible and safer alternative [13,115,130].

In contrast, findings for loading doses or single boluses are less consistent. Wu et al. [110] reported no relevant differences in the dose required to achieve adequate sedation between obese and non-obese children when dosing was based on TBW, suggesting that, in this specific context, TBW-based dosing may be acceptable.

Other authors [13,115] adopt a more conservative approach due to the narrow therapeutic index and the risk of bradycardia and hypotension. In this context, the recommendation to administer dexmedetomidine slowly or in divided doses to avoid peak concentrations [112] is consistent with safety warnings in clinical guidelines [115,116]. These considerations are derived from a combination of limited clinical data and model-based analyses. Nevertheless, the available evidence remains limited and heterogeneous. While PK models provide useful insights into the influence of body composition on drug disposition, direct clinical data are scarce, and therefore dosing considerations should remain cautious, clearly distinguishing between model-derived findings and empirically supported evidence.

### 4.12. Immunosuppressants

Cyclosporine shows increased exposure in obese pediatric patients when dosed based on TBW, which may increase the risk of adverse effects [113]. Consequently, these patients require lower maintenance doses to achieve therapeutic levels comparable to those in normal-weight patients, regardless of the body size descriptor used. These findings suggest that TBW is not an appropriate descriptor for cyclosporine dosing in this population.

These observations are supported by PK considerations in an observational study. Cyclosporine is a highly lipophilic drug with extensive binding to lipoproteins (90–98%) and a large Vd; however, the available evidence suggests that its distribution does not increase proportionally with excess adipose tissue. This is reflected in the consistent finding that obese and overweight patients require significantly lower doses per kilogram of body weight (mg/kg) to achieve similar concentrations compared with lean individuals. Similarly, dose requirements normalized by BMI or BSA are also reduced, reinforcing that drug exposure does not scale linearly with TBW. From a metabolic perspective, cyclosporine is predominantly metabolized by hepatic CYP3A4, and hepatic metabolic capacity does not increase in proportion to TBW in obesity. In addition, its primarily biliary excretion implies that the reduced renal function observed in obese patients does not directly influence cyclosporine Cl, although it may increase susceptibility to toxicity, further emphasizing the need for cautious dosing.

Importantly, although the use of IBW has been proposed in adult populations to mitigate these effects, pediatric evidence does not formally establish IBW as the standard descriptor. Nevertheless, the observed PK behavior in adolescents parallels that described in adults, supporting the need for dose reduction when TBW is used and highlighting the risk of overexposure if no adjustment is applied.

Current evidence supports these observations and suggests the use of IBW as the preferred body size descriptor, together with close TDM, given its narrow therapeutic index [13,115].

Despite the growing body of literature, important inconsistencies across studies were identified. These discrepancies may be explained by differences in study design, small and heterogeneous populations, variable definitions of obesity, and the use of different PK methodologies and outcome measures. In particular, variability in the selection of body size descriptors and dosing strategies complicates direct comparison across studies.

Common limitations across the included studies include small sample sizes, the predominance of observational and retrospective designs, and the lack of standardized reporting of PK and clinical outcomes. These factors limit the generalizability of the findings and contribute to uncertainty in the interpretation of dosing recommendations.

Overall, practical considerations emerging from this review include: (i) avoiding TBW-based dosing in hydrophilic drugs with limited adipose distribution; (ii) favoring FFM or AjBW for drugs with narrow therapeutic indices; and (iii) systematically incorporating TDM where available to guide individualized dosing.

This review has several limitations. First, there is substantial methodological heterogeneity across studies, including differences in the definition of obesity, age ranges, dosing regimens, and PK analysis methods. Second, the limited availability of data in specific obesity subgroups—particularly in cases of severe obesity—limits the generalizability of the findings. Finally, the overall quality of the available evidence is low, which limits the strength of the conclusions that can be drawn, and most studies lack clinical outcome data regarding the consequences of underdosing or overexposure to medications.

## 5. Conclusions

Despite representing a clinically relevant population, there is a marked scarcity of studies rigorously evaluating dosing requirements in children with excess body weight. This limited evidence base constitutes a potential risk for pharmacotherapy in this group and highlights an important safety gap compared with the general pediatric population.

Future research should prioritize well-designed prospective PK studies in obese pediatric patients, with standardized definitions of obesity and consistent use of body size descriptors. While PopPK and PBPK approaches may offer a valuable framework for hypothesis generation and for exploring sources of variability, their findings should not be considered equivalent to direct clinical evidence. Given the scarcity, heterogeneity, and variable quality of the available empirical data, model-based results should be interpreted with caution and require further prospective validation before informing clinical decision-making.

Overall, our findings highlight the need for higher-quality evidence to support safer and more effective dosing strategies in obese pediatric patients. At present, dosing decisions should be guided primarily by direct empirical evidence, TDM, careful clinical monitoring, and clinical judgment. In this context, individualized and model-informed approaches may serve as complementary tools to support clinical decision-making but should not replace direct clinical data or routine monitoring until further robust prospective validation becomes available. These conclusions should be interpreted in the context of the limited and heterogeneous evidence base and should not be considered definitive for clinical decision-making.

## Figures and Tables

**Table 1:** Availability of published studies on pediatric obesity among 100 HAM used in children and adolescents.

Pharmacological Group/Drug	Studies	Pharmacological Group/Drug	Studies	Pharmacological Group/Drug	Studies
Parenteral antidiabetics	3	Antihypertensives	1	Non-opioid analgesics	
IV and SC insulin (all release forms)		Amlodipine	1	IV paracetamol	3
Other antidiabetics		Captopril		Opioids	1
Glibenclamide		Clonidine		Codeine	
Glimepiride		Hydralazine		Fentanyl	5
Liraglutide	6	Labetalol		Methadone	1
Metformin	12	Methyldopa		Morphine	1
Semaglutide	2	Nifedipine		Oxycodone	
Oral anticoagulants		Sodium nitroprusside		Pethidine	
Acenocoumarol		Verapamil		Remifentanil	
Dabigatran		Diuretics		Tramadol	
Rivaroxaban		Furosemide		Antiepileptics	
Warfarin	1	Vasopressin and analogues		Valproic acid	3
Heparin and other antithrombotics		Desmopressin		Phenytoin	2
Alteplase		Aminoglycoside antibiotics		Phenobarbital	
Argatroban		Amikacin		Benzodiazepines	
Fondaparinux	1	Gentamicin	2	Clobazam	
Bivalirudin		Tobramycin		Clonazepam	
Heparin (unfractionated and low molecular weight)	14	Glycopeptide antibiotics		Clorazepate	
Urokinase		Vancomycin	11	Diazepam	1
Cardiac stimulants (including glycosides)		Antifungals: liposomal amphotericin B		Lorazepam	
Epinephrine/adrenaline	2	Antivirals		Midazolam	3
Digoxin		Acyclovir		Antipsychotics	
Dobutamine		Ganciclovir		Chlorpromazine	
Dopamine		Immunosuppressants		Haloperidol	
Phenylephrine		Mycophenolic acid		Risperidone	
Isoprenaline		Cyclosporine	1	Contrast agents	
Milrinone		Methotrexate		Meglumine amidotrizoate	
Norepinephrine/noradrenaline		Mycophenolate mofetil		Iopromide	
Antiarrhythmics		Sirolimus			
Adenosine		Tacrolimus		Electrolytes	
Amiodarone		Neuromuscular blockers		IV calcium (gluconate, chloride)	
Atenolol		Atracurium		IV sodium chloride (>0.9%)	
Esmolol		Cisatracurium		IV potassium (chloride and phosphate)	
Flecainide		Rocuronium		IV magnesium sulfate	1
Lidocaine		Succinylcholine	1	Others	
Procainamide		Vecuronium		Cardioplegic solution	
Propranolol				Hypertonic glucose (>20%)	
Anesthetics		Hypnotics-sedatives		Parenteral nutrition	
Etomidate		Dexmedetomidine	3	Intrathecal medication (any active substance)	
Ketamine	1	Chloral hydrate		Intrathecal baclofen	
Thiopental		Zolpidem			
Propofol	8				

IV: intravenous administration; SC: subcutanea administration

**Table 2:** Quality of evidence of the studies included in the systematic review.

	Observational Studies *n* = 38	Experimental Studies *n* = 21	PopPK/PBPK *n* = 20	Reviews *n* = 12
Low	5.26%	19.05%	5.00%	0.00%
Moderate	78.95%	71.43%	70.00%	50.00%
High	15.79%	9.52%	25.00%	50.00%

*n*: sample size; PBPK: physiologically based pharmacokinetic model; PopPK: population phamacokinetics.

**Table 3:** Summary of evidence type, amount, methodological quality, and consistency across pharmacological groups.

Pharmacological Group	Main Drugs	Type of Evidence	Amount of Evidence	Methodological Quality	Consistency of Evidence	Main Dosing Implication
Antidiabetics	Insulin, metformin, liraglutide, semaglutide	Observational/experimental/Review/PopPK/PBPK	Moderate–high	Moderate	Limited/heterogeneous	TBW appropriate for insulin; metformin may require adult-equivalent dosing; GLP-1 analogues require cautious, individualized approaches
Oral anticoagulants	Warfarin	Observational	Very low	Low	Limited	TBW-based dosing acceptable; avoid dose capping; close INR monitoring required
LMWH and antithrombotics	Enoxaparin, UFH	Observational, PopPK/PBPK	Moderate	Moderate	Consistent	Avoid TBW-based dosing; prefer FFM or AjBW; anti-Xa monitoring essential
Antihypertensives	Amlodipine, ramipril, valsartan	Observational/PBPK	Low	Low–moderate	Limited	TBW or BSA-based dosing; dose titration required due to PK variability
Neuromuscular blockers	Succinylcholine	Experimental	Very low	Low	Limited	TBW appropriate; AjBW may be considered in specific scenarios
Antibiotics–aminoglycosides	Gentamicin, tobramycin	Observational/PopPK	Low	Moderate	Consistent	Avoid TBW; prefer AjBW or FFM due to limited adipose distribution
Antibiotics–glycopeptides	Vancomycin	Observational, PopPK/Review	Moderate	Moderate	Moderate/partially consistent	TBW acceptable with TDM (preferably AUC-guided); variability requires monitoring
Antiepileptics	Valproic acid, phenytoin	Observational	Low	Low–moderate	Limited/heterogeneous	AjBW or TBW depending on drug; TDM essential
Anesthetics	Propofol, ketamine	Observational/PopPK/Review	Moderate	Moderate	Limited/mixed	Avoid TBW for induction (propofol); IBW/LBW preferred; maintenance may use TBW with scaling
Analgesics (non-opioid)	Paracetamol	Observational/PopPK/Review	Low	Moderate	Limited	TBW for loading dose; adjusted descriptors (e.g., NFM) for maintenance
Opioids	Fentanyl, morphine, methadone	Observational/PopPK/PBPK	Low	Low–moderate	Limited/heterogeneous	Avoid TBW (especially lipophilic drugs); prefer IBW or FFM to reduce accumulation risk
Benzodiazepines	Midazolam, diazepam	Observational, PopPK/PBPK	Moderate	Moderate	Moderate	TBW for loading; IBW/FFM for maintenance; high interindividual variability
Hypnotics and sedatives	Dexmedetomidine	Observational, PopPK/Review	Low	Moderate	Limited	Avoid TBW for maintenance; prefer IBW or FFM
Immunosuppressants	Cyclosporine	Observational	Very low	Low	Limited	Avoid TBW; prefer IBW; TDM essential

AjBW: Adjusted Body Weight; AUC: Area Under the Plasma Concentration–Time Curve; BSA: Body Surface Area; FFM: Fat-Free Mass; GLP-1: Glucagon-like peptide-1; IBW: Ideal Body Weight; INR: international normalized ratio; LBW: Lean Body Weight; LMWH: low molecular weight heparins; NFM: Normal Fat Mass; PBPK: Physiologically Based Pharmacokinetic; PK: pharmacokinetics; PopPK: Population Pharmacokinetic; TBW: Total Body Weight; TDM: Therapeutic Drug Monitoring; UFH: unfractionated heparin.

**Table 6:** Summary of studies describing dosing and PK and/or PD aspects of antihypertensive agents, cardiac stimulants, and neuromuscular blocking agents.

	Study/Design	Patients/Sample Size	Methods	Results	Dosing Conclusions
Epinephrine	Duong et al., 2016 [63] Observational Prospective Cross-sectional	Population: 200 children No stratification by obesity Age range: from 0.13 to 17 years. Obesity criterion: BMI ≥ P95 Administration of intramuscular adrenaline in patients with anaphylaxis	DS-MB was determined in children of varying weights and BMIs using ultrasound Measurements: Distance from the skin to the muscle fascia and to the femoral bone. Mean depth of the vastus lateralis muscle The following data were recorded: age (in months), weight (kg), height (cm), BMI, sex, and ethnicity	Key ultrasound findings: Higher BMI → greater distance from skin to muscle and bone. Significant linear regression: Skin-to-muscle distance: R^2^ = 0.3515, *p* < 0.001, r = 0.6. Skin-to-bone distance: R^2^ = 0.6429, *p* < 0.001, r = 0.8. African American women and children showed higher trends in BMI, as well as in distance to muscle and bone. Needle length adequacy of devices: EpiPen Junior (1.3 cm needle): 1 of 110 patients (0.9%) had DS-MB > 1.3 cm EpiPen adult (1.6 cm needle): 4 of 77 patients (5%) had DS-MB > 1.6 cm CDC (2.54 cm needle for <69 kg): 19 of 169 patients (11%) had DS-MB > 2.54 cm	It is not conclusive. Children with a higher BMI have a greater likelihood that the auto-injector needle will not reach the muscle, with a risk of SC administration
	Brown et al., 2018 [64] Narrative Review	Children <15 kg (including infants), Children and adolescents between 15–30 kg, Adults ≥ 30 kg, Obese populations (children and adults) Obesity is not defined. Administering adrenaline IM in patients with anaphylaxis	Review questions: Standardized doses of EAIs in relation to body weight, Effective injection depth according to needle length and patient anatomy (including obesity and underweight)	Children <15 kg receive an overdose with the 0.15 mg device. Children close to 30 kg receive a suboptimal dose with the same device Patients ≥40 kg using 0.3 mg receive only ~77% of the ideal dose for their weight Obese patients: 47% of obese children had a DS-MB > needle length; compared to only 13% of children of healthy weight	Inconclusive Children with obesity are much more likely to receive the dose SC The standard IM dose of adrenaline (0.3 or 0.5 mg) may be suboptimal in obese individuals, although this hypothesis requires confirmation in studies specifically designed for this purpose.
	**Empirical studies (PK and/or clinical data)**				
Nifedipine, amlodipine, enalapril y ramipril	Hanafy et al., 2009 [65] Observational retrospective cohort	Patients < 18 years 23 normal-weight patients 25 obese patients: For children 2 to 18 years: BMI ≥ P95 and for children under 2 years: weight ≥ P95 for age and sex Treatment with: CCB: nifedipine (short- and extended-release) or amlodipine ANGI: captopril, ramipril, enalapril andARB: losartan, valsartan, or candesartan As monotherapy or in combination with both in patients with nephrotic syndrome, renal insufficiency, or essential hypertension	Anthropometric data were recorded: age, sex, weight, height, BMI, and percentiles Pre- and post-treatment blood pressure measurements were taken. The primary response criterion was a ≥10% reduction in systolic and/or diastolic blood pressure Two outcomes were calculated: Classification of patients as responders or non-responders. Percentage reduction in systolic and diastolic blood pressure	Drugs used (CCB: 33 patients Angiotensin-interfering agents (ANGIs): captopril, ramipril, enalapril, losartan: 6 patients CCB + ANGI combination: 9 patients Dosage: per BSA (mg/m^2^/day) There were no significant differences in the average dose between the obese and non-obese groups. EFFICACY: Response to CCB Systolic blood pressure reduction ≥ 10%: Obese: 12.5% Non-obese: 52.9% → significant difference (*p* < 0.05) Diastolic blood pressure reduction ≥10%: Obese: 25% Non-obese: 58.8% → not significant, but a trend was observed. The absolute percentage reduction in blood pressure (systolic and diastolic) was also significantly lower in obese patients. EFFICACY: Response to ANGI There were no significant differences between obese and non-obese individuals in either systolic or diastolic blood pressure reduction Other factors influencing the response to CCBs: Corticosteroids: significantly reduced the diastolic response. Age, sex, and diagnosis were not significant in the multivariate model	Obesity reduces the effectiveness of CCBs in children with kidney disease, particularly in lowering systolic blood pressure Obesity does not affect the response ANGI
	PopPK/PBPK modeling studies				
Amlodipine	Burhanuddin et al., 2024 [66] PBPK modeling study with virtual clinical trials	It is not applicable because it is an FC model with multiple simulations.	PBPK-based simulation study using virtual clinical trials. Simcyp^®^ software (version 21). The study was divided into four main stages: 1. Development of a pediatric population with obesity. 2. Validation of this simulated population using known drugs (metformin and ceftazidime). 3. Verification of the amlodipine PBPK model in healthy, obese, and pediatric populations. 4. Evaluation of the impact of obesity on the PK of amlodipine in children, including analysis of the need for dose adjustments.	PBPK simulations were performed in 3 age groups: 2–6, 6–12, and 12–18 years. Cmax and AUC Fixed doses (2.5, 5, 10 mg/day), obese patients:Decreased 35.3% in AUC Decreased 20.5% in Cmax These decreases were statistically significant (*p* < 0.05) in all age groups. Doses based on TBW: There were no significant differences in Cmax or AUC between obese and non-obese children aged 2–6 years. In older age groups (9–12 years), there were significant differences in Cmax (*p* < 0.05). Vss was significantly higher in obese children than in non-obese children in all age groups. The Cl was also significantly higher in obese individuals compared to non-obese individuals, both with fixed doses and with TBW-based doses.	Dosage by TBW (in the age range of 2–12 years) may be considered. If administered by fixed dose, the dose to obese patients may require doses 1.25–1.5 times higher than that administered to non-obese patients; without exceeding 10 mg per day.
Succinylcholine	Rose et al., 2000 [67] Controlled Randomized controlled trial	Patients aged 6 to 15 years 30 adolescents Obesity BMI > 30	A prospective clinical trial was conducted to construct a dose-response curve for succinylcholine in obese adolescents under general anesthesia. The study evaluated the effect of three doses (100, 150, and 250 µg/kg) on neuromuscular function. 20 patients received either 100 µg/kg or 250 µg/kg of succinylcholine. The final 10 patients received 150 µg/kg. Neuromuscular blockade was monitored using ED50, ED90, and ED95.	Effective doses to achieve different levels of blockade: ED50: 152.8 µg/kg (95% CI: 77.8–299.5) ED90: 275.4 µg/kg (95% CI: 142–545.7) ED95: 344.3 µg/kg (95% CI: 175.3–675.3) These figures are very similar to those previously reported in non-obese adolescents, especially for ED50.	TBW

ARB: angiotensin II receptor blockers; ANGI: angiotensin-converting enzyme inhibitors; BMI: body mass index; BSA: body surface area; CCB: calcium channel blockers; CDC: Centers for Disease Control and Prevention; Cl: total body clearance; Cmax: maximum plasma concentration; DS-MB: distance from skin to muscle and bone; EAIs: epinephrine auto-injectors; ED50,90,95: dose that produces 50, 90, 95% of the maximum effect; IM: intramuscular administration; PBPK: physiologically based pharmacokinetic models; PD: pharmacodynamics; PK: pharmacokinetics; SC: subcutaneous administration; TBW: total body weight.

**Table 7:** Summary of studies describing dosing and PK and/or PD aspects of antibiotics.

	Study/Design	Patients/Sample Size	Methods	Results	Dosing Conclusions
Gentamicin	Empirical studies (PK and/or clinical data)				
	Choi et al., 2011 [68] Retrospective Cohort Observational Study	Patients aged 2 to 18 years. 50 patients: 25 obese/25 normal weight Obesity BMI ≥ P95 Scheduled gentamicin treatment	Compare serum concentrations (peak and trough) and Vd between obese and non-obese children	Obese patients showed significantly higher peak (8.17 vs. 7.06 µg/mL) and trough concentrations; *p* < 0.05 Lower doses per TBW: 1.86 vs. 2.25 mg/kg; *p* < 0.01 The Vd was significantly lower in obese patients (0.20 vs. 0.28 L/kg); *p* < 0.01 No significant differences in nephrotoxicity were found	Obese children require lower doses per mg/kg of actual weight due to a lower Vd. Empirical dose reduction and individualization through monitoring of serum levels are suggested
	PopPK/PBPK modeling studies				
	Moffett et al. [69] 2018 Retrospective Cohort Observational Study with PopPK Analysis	Patients aged 2 to 18 years 520 patients: 21.3% obese, 15.8% overweight Obesity: BMI ≥ P95 Overweight: ≥P85 Indications: febrile neutropenia, appendicitis, sepsis, among others	Population PK analysis (NONMEM) comparing dosage by TBW, AjBW and FFM	The two-compartment PK model with allometrically scaled FFM Cl (L/h) = 5.77 (FFM/70)^0.75^ × 0.516 ^LOG(SCR/0.47)^ × (years/9.9)^0.138^ Vd central (L) = 23.7 · (FFM/70) Vd peripheral (L)= 18.3 · (FFM/70) FFM reduced dosage weight by 32.1% in obese patients compared to AjBW. Doses of 3–3.5 mg/kg/8 h reach peaks of 6–10 mg/L or 7 mg/kg/24 h (based on FFM)	The use of FFM may be considered for weight adjustment in empirical gentamicin dosing in children aged 2 to 18 years, regardless of their body habitus
Vancomycin	Empirical studies (PK and/or clinical data)				
	Moffett et al. [70] 2011 Retrospective observational case-control study	Patients aged 2 to 18 years 24 matched pairs. Obesity: BMI ≥ P95 Indications: neutropenic fever, central line infections, pneumonia.	Comparison of trough concentrations starting from the third dose. Dose: 15 mg/kg/per TBW	PK parameters (measured in 4 obese patients) T_½_: 2.9 ± 0.29 h Vd: 0.35 ± 0.15 L/kg Dose and levels Dose: 14.1 ± 1.5 mg/kg (obese) vs. 14.9 ± 0.9 mg/kg (controls) *p* = 0.03 Trough levels: 6.9 ± 4.3 µg/mL (obese) vs. 4.8 ± 0.05 µg/mL (controls) *p* = 0.052	Obesity did not significantly alter trough concentrations; overweight children should receive TBW-based doses
	Miller et al., 2011 [71] Retrospective cohort observational study	Patients aged 2–17 years 187 patients (232 diets): 129 normal weight, 23 overweight, 35 obese. Overweight BMI ≥ P85 and obesity ≥ P95 Indication: respiratory, skin, and soft tissue infections	Comparison of doses and trough therapeutic levels Target: Trough concentrations of 5–15 µg/mL between normal weight and overweight/obese groups Dose: 20 mg/kg/6–8–12 h with monitoring at the 3rd dose	Initial dose Normal weight: 17.5 ± 4.4 mg/kg Overweight/obese: 16.6 ± 3.9 mg/kg; *p* = 0.295 Therapeutic levels: Obese: (9.6 ± 8.9 µg/mL) vs. normal weight group (7.4 ± 5.7 µg/mL); *p* = 0.03 Obesity was associated with a lower likelihood of achieving therapeutic levels (OR 0.34; *p* = 0.037). Nephrotoxicity showed no significant difference (8.5% vs. 3%)	Consider TBW-based dosing without limiting to 1 g. Empirical dosing every 8 h (trough 5–15 µg/mL) or every 6 h in complicated infections (trough 15–20 µg/mL) is recommended
	Nassar et al., 2012 [72] Prospective cohort study	Patients: 2 months–18 years. 51 children: 26 overweight, 34 normal weight, and 15 underweight. Obesity in children <2 years: ≥P98 Overweight/obesity in children > 2 years: BMI ≥ P85 Indication: Empirical treatment of suspected catheter-related infections, neutropenic fever, and pneumonia	Evaluate the suitability of a 20 mg/kg every 12 h regimen and calculate PK parameters (CL, Vd, t_1/2_) Target trough concentration: >10 mg/L and AUC/MIC ratio > 400	Mean trough concentration: 3.36 ± 2.58 mg/L; only 3% of samples reached the therapeutic range. Only one child achieved an AUC/MIC > 400 PK parameters (Vd, CL, t_1/2_): There were no significant differences between weight groups Efficacy: Positive clinical response in all patients. Adverse effects: No renal or ototoxicity	Dosing by TBW shows no difference between obese and non-obese individuals without limiting the dose to 1 g More frequent and higher doses (possibly every 6 h) are needed to achieve current guideline targets in all weight groups Perform TDM
	Heble et al., 2013 [73] Retrospective observational case-control study	Patients aged 2–18 years. 42 overweight/obese patients matched with 84 normal-weight patients Obesity: BMI ≥ P95 Indications: skin infections, pneumonia, sepsis, meningitis	Vancomycin dosage by TBW and stratified by age: 2–8 years: 20 mg/kg/6 h, 9–13 years: 20 mg/kg/8 h, and 14–18 years: 15 mg/kg/8 h. Maximum initial dose: 1500 mg. Target levels: >10 mg/L	Plasma concentrations: Median concentration in obese patients: 14.4 µg/mL vs. 10.5 µg/mL in normal weight patients (*p* < 0.001) Concentrations > 20 µg/mL were more frequent in the obese group (*p* = 0.016). Adverse effects: no significant differences were observed	Obese pediatric patients may have elevated trough levels with TBW-based doses. Close monitoring of plasma levels is recommended
	Eiland et al., 2014 [74] Retrospective Cohort Observational	Patients aged 2–18 years. 98 patients: 48 normal weight/50 overweight/obese Obesity: BMI ≥ P95 Indications: skin, soft tissue, and respiratory tract infections; osteomyelitis; meningitis	Review of medical records from 2005–2010 in patients aged 2 to 18 years and describe the initial dosage and resulting serum concentrations in normal weight versus overweight/obese children	Only 14.2% reached the target valley (10–20 mg/L) Mean dose: 53.6 mg/kg/day (normal weight) vs. 51.6 mg/kg/day (obese). No significant difference There was no significant association between demographic characteristics and target achievement. One obese patient presented with renal dysfunction at levels > 20 mg/L	The initial dosage for overweight or obese patients should not be different from that for normal weight patients; use the TBW
	Khare et al., 2021 [75] Multicenter retrospective cohort observational study	Patients aged 2–18 years 1099 children: 162 underweight, 599 normal weight, 153 overweight, 185 obese. Obesity: BMI ≥ P95 Indication: TBW-guided vancomycin treatment	Study conducted in three hospitals. Examination of the influence of body weight metrics (TBW, IBW, AjBW, BSA, and allometric weight) on vancomycin concentrations and evaluation of AUC/MIC as a measure of exposure	Cmin: 75% had subtherapeutic baseline concentrations (<10 g/mL) AUC/MIC: 63% of patients did not reach the therapeutic target ≥ 400 Cmin in obese children: 19% higher (9.2 vs. 7.7 g/mL) receiving lower doses per kg Predictive Capacity of Weight Metrics: TBW and allometric weight predicted AUC with 80% accuracy in all weight groups	TBW is a reliable predictor of AUC exposure. A dose of 60 mg/kg/day based on TBW is recommended Children under 12 years of age may require higher doses due to their increased Cl
	Khare et al., 2020 [76] Systematic review and meta-analysis	Patients aged 30 days–18 years 7 studies included with 521 children Obesity (BMI ≥ P95) and overweight (BMI ≥ P85) Various indications of serious infections	Medline search Quality of studies using the Newcastle-Ottawa scale Dosage: 40 and 60 mg/kg by TBW (in most studies every 6 or 8 h)	4 studies were included in the meta-analysis. Trough levels: a small but significant difference was observed, with levels 2.2 µg/mL higher in children with overweight/obesity than in those with normal weight when TBW was used Adverse effects: no significant differences in nephrotoxicity were observed	Using TBW for dosing in obese children may lead to higher concentrations and potential toxicity Further studies are needed to guide targeted dosing strategies
	PopPK/PBPK modeling studies				
	Le et al., 2015 [77] Observational matched case-control study.	Patients aged 3 months-21 years 174 participants (87 overweight/obese and 87 controls) Obesity: BMI ≥ P95. Matched for age, creatinine and nephrotoxic use	Population PK modeling to compare body mass index (BMI, AjBW, LBW, IBW, allometric weight, BMI, BSA) that estimate Vd and Cl Dose for obese subjects: 41.9 mg/kg/day Dose for control subjects: 47.4 mg/kg/day for at least 48 h	Mean Cl was 0.11 L/kg/h in cases versus 0.12 L/kg/h in controls Allometric weight (*p* ^0.75^) was the best final descriptor Mean Vd was 0.56 L/kg in cases and 0.58 L/kg in controls TBW was the final descriptor t_1/2_: 4.1 h in cases and 3.4 h in controls Detection of 4 cases in the obese group with a significant increase in SCR (34.8%)	TBW and allometric weight may be appropriate estimates for Vd and CL, respectively PK differences are small and unlikely to require dose adjustments compared to non-obese children
	Moffett et al., 2019 [78] Retrospective cohort observational study with PopK modeling	Patients < 19 years old and weighing ≥ 70 kg 196 patients: 75% with obesity BMI ≥ P95	Population PK analysis using NONMEM v.7.3 software to determine the best dosing strategy (weight-based vs. fixed dose) to achieve an AUC ≥ 400 mg·h/L	FFM was the best PK descriptor. Cl: 18.6 L/h per 70 kg of FFM Simulations showed that 20 mg/kg/6 h based on FFM achieved greater success (AUC/MIC ≥ 400 and trough < 20 mg/L)	Dosage in pediatric patients weighing ≥ 70 kg may be based on FFM, with additional adjustments according to Clcr
	Smit et al., 2021 [79] Retrospective cohort observational study with PopPK modeling	Patients aged 1–18 years 1892 children: 1344 normal weight/247 overweight/301 obese Severe infections with varying levels of renal function.	PK simulations using NONMEM 7.4 software Dosage: 5 and 20 mg/kg, administered 2, 3, or 4 times daily as a 60-min infusion Covariates: age, sex, TBW, CLcr, race, and neutropenic status Target AUC_24_: 400–700 mg·h/L	Two-compartment model Cl for a 22.1 kg patient: 2.12 L/h; increased significantly with TBW (exponent of 0.745) and CLcr. Vd central (8.9 L) and peripheral (12.3 L) increase with TBW. Dosage guidelines based on weight and renal function: for patients with Clcr > 90 mg/dL: If <30 kg: 15 mg/kg/6 h If 30–70 kg: 15 mg/kg/8 h If >70 kg: 18 mg/kg/12 h Current guidelines (IDSA/BNFc) may lead to potentially toxic exposures in obese patients or those with renal impairment	A guideline based on TBW and CLcr is proposed, according to the bedside Schwartz equation
	Zhang et al., 2023 [80] Observational with comparative PopPK modeling	Patients aged 10–18 years 125 adolescents and 81 adults with overweight/obesity Obesity in adolescents was defined as BMI ≥ P85 Adults: overweight BMI ≥ 25 kg/m^2^ and obesity as BMI ≥ 30 kg/m^2^. They received vancomycin as per routine clinical practice	PK analysis using NONMEM 7.4 to investigate whether CL in obese adults predicts CL in obese adolescents. Adolescent dose: 15–20 mg/kg every 6–12 h. Adult dose: 500–1500 mg every 8–12 h or continuous infusion of 1000–3500 mg/day Covariates: standard body weight and excess body weight	Vancomycin Cl was higher in adolescents than in adults (*p* < 0.01) Cl: 6.03 L/h with a mean body weight (TBW) of 100 kg and age 12 years In adolescents, males had a Cl 21% higher than females. Cl correlated better with standard weight than with excess weight	Vancomycin dosages may not be directly extrapolated from obese adults to obese adolescents It should be considered that the Cl is generally higher in the adolescent population

AjBW: adjusted body weight; AUC: area under the curve BMI: body mass index; BNFc: British National Formulary for Children; BSA: body surface area; Cl: total body clearance; CLcr: creatinine clearance; Cmin: trough concentration; FFM: fat-free mass; IBW: ideal body weight; IDSA: Infectious Diseases Society of America; LBW: lean body weight; MIC: minimum inhibitory concentration; percentile; NONMEM: nonlinear mixed effects modeling; P: percentile; PBPK: Physiologically based pharmacokinetic model; PD: pharmacodynamics; PK: pharmacokinetics; PopPK: population pharmacokinetics; SCr: serum creatinine (mg/dL); t½: elimination half-life; TBW: total body weight; TDM: therapeutic drug monitoring; Vd: volume of distribution.

**Table 8:** Summary of studies describing the dosing and PK and/or PD aspects of antiepileptic drugs.

	Study/Design	Patients/Sample Size	Methods	Results	Dosing Conclusions
Valproic acid	Suemaru et al., 1998 [81] Cross-sectional prospective observational study	Children > 12 years old receiving VPA and CBZ VPA: 5 obese/12 normal weight Low group: BMI < 20 kg/m^2^ Normal weight group: BMI between 20 and 25 kg/m^2^ Moderately obese group: BMI > 25 kg/m^2^	VPA: 800 mg per day. Serum measurements 2–3 h post-administration of the morning dose. Study of the correlation between plasma concentrations and BMI, IBW, or TBW	VPA A significant correlation was found between IBW and VAL concentrations (r = 0.643, *p* < 0.05), but no correlation was found with TBW or BMI No significant differences were found in serum concentrations between the BMI groups (lean, normal, obese)	The loading and maintenance doses of VPA should be based on IBW. However, monitoring of levels is necessary in patients with BMI < 20 or >25 (lean and obese)
	Arya et al., 2016 [82] Observational study of a randomized clinical trial cohort	Children with childhood absences, from age 2 to adolescents Overweight group: 147: (BMI ≥ P85 overweight and BMI ≥ P95 obese) 297: US child population reference group: NHANES	This study is a secondary analysis of a clinical trial conducted at 32 centers. The original trial compared the effectiveness of ETX and VPA 24-h dietary intake was monitored. The study explored whether BMI affected the response to treatment with the drugs: ETX, VPA, and LTG Response was assessed at 16–20 weeks of SF and FFF. Minimum Cmin and AUC were determined	Prevalence of excess weight: Overweight: 19.3% in CAE vs. 13.8% in NHANES PK data: There were no significant differences in AUC and Cmin between children with BMI ≥ P85 and those with BMI below that for any of the three drugs: ETX, LTG, VPA Effectiveness: ETX (obese): OR for FFF: 2.75 (*p* = 0.047) VPA (obese): OR for SF: 4.89 (*p* = 0.040) LTG (obese): Worst clinical response, significantly less effective (OR for FFF = 0.24, SF = 0.19)	It is not conclusive
	Good et al., 2001 [83] Retrospective Cohort Observational Study	16 patients aged 6–12 years with psychiatric disorders. Overweight: patients with a weight > 15% above the IBW	Dosage of 15 mg/kg/day, divided into two daily doses Treatment groups by dose type: Dose according to TBW Dose adjusted according to IBW: IBW + 40% × (TBW − IBW) Cmin is determined on day 5 of treatment The therapeutic range was considered to be between 50–120 µg/mL	Plasma levels Observed range: 46–146 µg/mL. 13 of 16 children (81.3%) achieved levels within the therapeutic range (50–120 µg/mL) Dosing and comparison by weight type used: Overweight children receiving TBW-based doses: Had significantly higher serum levels, and some reached supratherapeutic levels (>120 µg/mL) with a higher incidence of side effects Overweight children receiving AjBW doses: Had more appropriate levels within the therapeutic range and fewer adverse effects	AjBW with a factor of 0.4
Phenytoin	Messinger et al., 2015 [84] Retrospective Cohort Observational Study	Children aged 2–18 years 15 overweight patients (BMI ≥ P85) 52 normal weight patients (BMI < P85) Fosphenytoin loading dose (PFH prodrug) in patients with status epilepticus	Determine the correlation between BMI and serum concentrations at 2 or 4 h post-administration (total and free) Demographic and clinical data: Age, sex, weight, height Fosphenytoin dose (mg/kg) Albumin concentration Presence of drug interactions Subsequent use of antiepileptic drugs (phenobarbital or levetiracetam), as an indicator of therapeutic failure	PK Data Mean total PFH concentration: Normal weight: 21.9 ± 6.7 mg/L Obese: 21.9 ± 6.9 mg/L Albumin-adjusted concentration: Normal weight: 24.1 ± 8.1 mg/L Obese: 23.5 ± 7.1 mg/L There were no statistically significant differences in PFH levels between groups (neither total nor adjusted) (*p* > 0.05) BMI was not a significant predictor of serum PFH concentrations Clinical efficacy No significant differences between BMI groups with respect to additional medication (*p* > 0.05)	Dose calculated by TBW without the need for dose adjustments in obese patients
	Prusakov et al., 2018 [85] Observational Retrospective Cohort	Children aged 2–18 years 12 obese patients (BMI ≥ P95 25 normal-weight patients Administration of a fosphenytoin loading dose of at least 10 mg PhE/kg in patients with status epilepticus	Determination of serum PFH level after dosing, weight, and height The apparent Vd was calculated by dividing the total administered dose of fosphenytoin by the total serum PFH concentration Therapeutic levels were considered to be between 10 and 20 mg/mL	Mean fosphenytoin dose: 23.9 ± 8.1 mg PhE/kg. Mean total serum PFH level: Non-obese: 25.3 ± 6.5 mg/mL Obese: 29.5 ± 7.6 mg/mL (*p* = 0.09 → not significant) Free serum level (only available in some cases): Non-obese: 2.9 mg/mL Obese: 3.75 mg/mL (*p* = 0.3) Apparent Vd: Non-obese: 0.92 ± 0.26 L/kg Obese: 0.97 ± 0.48 L/kg No significant difference (*p* = 0.76) No significant correlation was found between BMI and Vd (Pearson’s coefficient = 0.08, *p* = 0.63)	Dosage calculated by TBW and adjusting the fosphenytoin loading dose based on obesity status in pediatric patients is not justified

AE: childhood absence epilepsy; AjBW: adjusted body weight; BMI: body mass index; CAE: childhood absence epilepsy; CBZ: carbamazepine; Cmin: trough concentration; ETX: ethosuximide; FFF: freedom from failure; IBW: ideal body weight; LTG: lamotrigine; NHANES: national health and nutrition examination survey; PFH; phenytoin; PhE: phenytoin equivalent; PK: pharmacokinetics; SF: seizure freedom; TBW: total body weight; Vd: volume of distribution; VPA: valproic acid.

**Table 9:** Summary of studies describing the dosing and PK and/or PD aspects of anesthetic drugs.

	Study/Design	Patients/Sample Size	Methods	Results	Dosing Conclusions
Ketamine	Street and Gerard, 2014 [86] Prospective cohort study	43 patients Age: 12–17 years 17 with obesity (BMI > 25 kg/m^2^) vs. 26 with normal weight (BMI ≤ 25 kg/m^2^) IV procedural sedation with ketamine in the pediatric emergency department	Fixed-dose ketamine protocol comparing performance in patients with normal weight vs. obesity: Initial 50 mg IV (over 30–60 s) + 25 mg IV boluses as needed to achieve/maintain RSS ≥ 5 before/during the procedure Continuous monitoring and structured recording of AEs during sedation and 12–24 h post-sedation follow-up	Clinical sedation results: RSS ≥ 5 in 35/43 patients (81.4%) with the initial bolus; the remainder achieved this level after an additional 25 mg Required dose: Obesity: median total dose 100 mg (1.16 mg/kg AjBW; 1.35 mg/kg IBW) Normal weight: median total dose 75 mg (1.31 mg/kg AjBW; 1.31 mg/kg IBW) Patients with obesity received fewer mg/kg based on AjBW than normal-weight patients (1.16 vs. 1.31 mg/kg, *p* = 0.01), whereas doses based on IBW were similar (*p* = 0.67) AEs: nausea and vomiting, occasional agitation, and 1 episode of transient airway obstruction	The fixed-dose protocol (50 mg IV + 25 mg as needed) achieved adequate sedation in most patients and can be used regardless of BMI. In patients with obesity, this regimen provides exposure similar to that estimated using IBW and may avoid potential overdosing TBW weight is used
Propofol	Empirical studies (PK and/or clinical data)				
	Olutoye et al., 2012 [87] Prospective observational study	80 patients Age: 3–17 years Obese: n = 40 vs. normal weight: n = 40. Obesity: BMI ≥ P95 Normal weight: BMI 25th–84th P. Healthy ASA I-II children aged 3–17 years undergoing induction of general anesthesia for surgical procedures (propofol as the induction agent)	Prospective study using a Biased Coin Design dose-finding approach. Intervention: IV bolus of propofol administered over 10 s Primary endpoint: blinded observer assessment of loss of eyelash reflex at 20 s Doses adjusted in 0.25 mg/kg increments according to the response of the previous patient Objective: to estimate the ED95 of propofol for loss of consciousness in obese vs. normal-weight patients and to derive a practical induction dosing rule	ED_95_ in obese patients: 2.0 mg/kg (approximate 95% CI 1.8–2.2) ED_95_ in normal-weight patients: 3.2 mg/kg (approximate 95% CI 2.7–3.2) AEs: both groups showed a significant decrease in blood pressure after propofol administration (decrease ≥ 10 mmHg at 2 min). Standard dosing in obese patients may increase the risk of hypotension	In patients with obesity (BMI ≥ 95th percentile), the recommended induction dose is 2.0 mg/kg. In contrast, in patients without obesity (BMI < P85), the recommended induction dose is 3.2 mg/kg (within the range recommended in the prescribing information)
	Chidambaran et al., 2013 [88] Prospective observational study	20 evaluable patients. Age: 9–18 years 19/20 met criteria for morbid obesity. Obese: BMI > P95 Morbidly obese BMI >P99 Elective surgery lasting at least 60 min, including laparoscopic bariatric surgery, laparoscopic cholecystectomy/appendectomy, and orthopedic procedures	Prospective evaluation of the quality of TIVA with propofol in adolescents with morbid obesity Clinically titrated propofol TIVA (BIS masked) Recorded variables: dose/infusion rates, hemodynamics, induction times, postoperative RSS, and adverse respiratory events. Intraoperative and postoperative plasma concentrations were measured	Clinical results: Mean propofol concentration: 6.2 mg/L during maintenance and 1.8 mg/L at emergence. Excessive depth: BIS < 40 during >90% of the maintenance period Delayed emergence: eye-opening time 25.8 min. Postoperative somnolence: RSS ≥ 4 in the first 30 min. AEs postoperative events in 6/20 patients (30%); probability increased by 14% for each BMI unit	Recommendation: titrate propofol to BIS targets to minimize overdosing The induction dose correlated better with LBM than with TBW
	Rogerson et al., 2017 [89] Retrospective observational study	1976 patients Age: 2–21 years. BMI percentile categories: underweight < 5, normal 5–84, overweight > 85, obese > 95 Deep procedural sedation in pediatric oncology patients undergoing lumbar puncture and/or bone marrow aspiration	All patients received adjunctive ketamine + intermittent propofol boluses. Objective: to assess the association between BMI category and propofol requirement and adverse event rates Variables: cumulative propofol dose (mg/kg) and AEs (hypoxemia < 90% with O_2_, apnea requiring bag-mask ventilation, bradycardia, or hypotension > 20% from baseline)	Clinical results: Overweight and obese children required less propofol (mg/kg TBW) than normal-weight children (*p* < 0.01). 100% of procedures were completed successfully AEs: no increase in overweight/obese vs. normal-weight patients. Underweight patients had more overall AEs (10.6% vs. 3.5% in normal-weight patients) and a higher risk of hypoxia and apnea in logistic regression	IBW may be a more appropriate scaling metric than TBW to guide dosing in obesity
	Sahinovic et al., 2018 [90] Review	Eleveld model integrating data from 1033 patients (672 men and 361 women) from 30 previous studies. Age: 0–88 years Obesity descriptors: BMI, TBW, and AjBW Propofol used for anesthesia and sedation	Evidence update based on MEDLINE/PubMed regarding propofol PK/PD, including population and unified models (Eleveld) Objective: to synthesize recent advances and their practical application to dosing/modeling, including extremes such as pediatrics and obesity	Supports unified models (Eleveld) with good performance in pediatric and obese populations. V1 (central compartment) does not increase linearly with body weight, but rather follows a sigmoidal function, resulting in a lower induction dose per kg in obesity TBW better describes Vd (loading dose). FFM better describes CL (maintenance dose). Serious adverse effects: PRIS at high doses; avoid >5 mg/kg/h for >48 h	Induction: do not scale linearly by TBW, as V1 shows sigmoidal behavior and reaches a plateau; the dose per kg should therefore be lower in patients with obesity. Maintenance: should be guided more by CL, which is better related to FFM
	PopPK/PBPK modeling studies				
	Diepstraten et al., 2012 [91] Prospective observational study	20 patients Age: 9–18 years. Pediatric/adolescent obese or morbidly obese patients: n = 20 Obesity: BMI >30 kg/m^2^, stated to be equivalent to body weight > P95 Children/adolescents scheduled for bariatric surgery or other elective procedures under propofol anesthesia for ≥60 min	Prospective PK study in pediatric obese/morbidly obese patients. Standard propofol anesthesia (induction followed by infusion at a standardized AjBW-based rate, then titrated according to clinical signs) Population modeling with NONMEM and covariate evaluation (TBW, BMI, IBW, LBW, and age) Objective: to characterize propofol Cl in pediatric morbid obesity and provide the basis for a dosing algorithm	PK results: Two-compartment model (a three-compartment model did not improve fit) TBW was the most predictive covariate for plasma Cl, with an allometric function: Cl (L/min) = 1.70 × (TBW/70)^0.8^ Compared with non-obese pediatric models, extrapolation of the Kataria model overestimated Cl in this TBW range	Maintenance doses of propofol in children/adolescents with morbid obesity should be based on TBW using allometric scaling rather than linear mg/kg dosing
	Diepstraten et al., 2013 [92] Meta-analysis	94 patients Age: 9–79 years; 37–184 kg. Obese adults: n = 20 Non-obese adults: n = 40 Obese pediatric patients: n = 20 Non-obese pediatric patients: n = 14 Obesity: cohorts labeled as obese vs. non-obese from 5 previous studies. BMI was evaluated as a descriptor Propofol use in different surgical procedures	Population PK meta-analysis combining data from 5 studies in obese and non-obese patients. PK model: 3 compartments (Cl, V1, V2, V3, Q2, Q3). Covariates: TBW, BMI, IBW, LBW, sex, and age	PK results: Cl increased with TBW according to an allometric relationship (exponent 0.77) Age: influenced Cl bilinearly, increasing up to 41 years and decreasing thereafter Q3 was linearly related to TBW No robust relationship was found between Vd and body-size descriptors Clinical results: the final model substantially reduced interindividual variability in Cl. Obese adolescents did not behave like adults with the same TBW (lower Cl)	Propofol maintenance dosing: dosing may be based on TBW plus age, as both better explain changes in Cl For induction dosing, the article suggests that the evidence is less robust in this meta-analysis and that further research is needed
	Eleveld et al., 2014 [93] Population PK modeling	660 patients (obese vs. non-obese) Age: 0.25–88 years Weight: 5.2–160 kg Obesity: BMI ≥ 30 kg/m^2^ Propofol for IV anesthesia (perioperative context)	Population PK modeling using pooled data from multiple studies to build a general model applicable across a wide range of ages and body weights Covariates included weight, age, sex, and patient status. Performance was evaluated across BMI subgroups.	PK results: Three-compartment model V1: reached a plateau and did not scale linearly with body weight (plateau around 30 kg) Performance in obesity: predictive performance was worse in BMI ≥ 30 vs. <30, indicating greater uncertainty in obesity	Induction: suggest avoiding TBW-based dosing Maintenance: suggest adjusting according to physiological allometric scaling of CL rather than 1:1 proportionality with TBW
	Chidambaran et al., 2015 [94] Prospective observational study	26 patients (children/adolescents with severe obesity) Age: 9–18 years (BMI 31–69 kg/m^2^). Obesity: BMI > P95 (inclusion criterion) Bariatric or other elective surgery under IV propofol anesthesia for ≥60 min	Prospective PK/PD study in severe obesity Clinically titrated propofol anesthesia (team blinded to BIS) using infusion schemes Development of a population PK/PD model with NONMEM and Monte Carlo simulations to design a maintenance regimen targeting BIS 50 ± 10	PK/PD results: Three-compartment model + sigmoidal PD model (inhibitory Emax) with ke0; adequately described 375 concentrations and 3334 BIS values TBW was the most predictive covariate for Cl with allometric scaling of 0.75 EC50 (effect site) = 3.19 μg/mL, suggesting no relevant effect of obesity on the concentration-BIS relationship	Induction dose: best correlation with AjBW (1.4 mg/kg based on AjBW) Maintenance: step-down infusion allometrically scaled according to TBW (155, 120, and 85 μg/kg/min in successive phases; exponent 0.75)

AEs: adverse events; AjBW: adjusted body weight; ASA: American Society of Anesthesiologists physical status classification; BIS: bispectral index; BMI: body mass index; Cl: total body clearance; EC50: effect-site concentration producing 50% of the maximum effect; ED95: 95% effective dose; Emax: Maximal reduction in BIS; PK/PD: pharmacokinetics/pharmacodynamics; FFM: fat-free mass; IBW: ideal body weight; IV: intravenous; ke0: first-order equilibration rate constant; LBM: lean body mass; LBW: lean body weight; NONMEM: nonlinear mixed-effects modeling; P: percentile; PRIS: propofol infusion syndrome; Q2: intercompartmental clearance between the central compartment (V1) and the first peripheral compartment (V2); Q3: intercompartmental clearance between the central compartment (V1) and the second/third peripheral compartment (V3) in a 3-compartment pharmacokinetic model; RSS: Ramsay Sedation Score; TBW: total body weight; TIVA: total intravenous anesthesia; Vd: volume of distribution; V1: volume of distribution of the central compartment; V2: volume of distribution of the first peripheral (rapid) compartment; V3: volume of distribution of the second peripheral (slow) compartment.

**Table 10:** Summary of studies describing dosing and PK and/or PD aspects of opioid and non-opioid drugs.

	Study/Design	Patients/Sample Size	Methods	Results	Dosing Conclusions
Acetaminophen	Empirical studies (PK and/or clinical data)				
	Zempsky et al., 2020 [95] Retrospective review	0 years–adolescents Sample size not specified Pediatric patients with obesity (acute pain, fever, NAFLD	Literature review on acetaminophen dosing in obesity	Increased Cl with TBW nonlinear; risk of overdosing when using TBW Variability in prescribing practices; risk of both underdosing and hepatotoxicity	No consensus. TBW-based dosing may lead to toxicity, but standard dosing may result in underexposure
	PopPK/PBPK modeling studies				
	Hakim et al., 2018 [96] Observational prospective cohort with PopPK modeling	Adolescents (>12 years) n = 11; obesity (BMI ≥ P95 or BMI ≥ 40 kg/m^2^) Severe obesity undergoing bariatric surgery	PopPK analysis Single IV dose (1000 mg) Serial acetaminophen concentrations.	Subtherapeutic concentrations (<10 µg/mL) within <2 h in all patients Cl best predicted by TBW with allometric scaling NFM better descriptor for Vd	Standard fixed dosage insufficient TBW (allometric) for Cl and NFM for distribution
	Anderson and Cortinez, 2023 [97] Population PK modeling study	6–12 years; pediatric patients with overweight/obesity Overweight/obesity (BMI ≥ 25–30 kg/m^2^). Sample size not specified. Undergoing surgery.	Loading dose (mg/kg TBW) and maintenance dosing modeled using allometry and body composition descriptors	Vd influenced by TBW Cl increases with size but non-linearly NFM better predicts Cl	TBW for loading dose. NFM with allometric scaling for maintenance dosing
Fentanyl	Empirical studies (PK and/or clinical data)				
	Okada et al., 2024 [98] Interventional pharmacokinetic study	2–18 years n = 30; 15 obesity (BMI ≥ P95), 15 normal weight (BMI P5–P85) Undergoing tonsillectomy	Single IV dose 1 mcg/kg based on TBW. Serial plasma concentrations measured up to 180 min Covariates: TBW, FFM, BMI, age	Plasma concentration at 5 min: obesity 0.88 ng/mL vs. normal weight 0.53 ng/mL (*p* = 0.01). Higher early exposure in obesity; concentrations converged after 30–120 min	TBW-based bolus dosing leads to higher peak concentrations. FFM better predictor of central volume. Recommend FFM-based dosing for bolus to reduce risk of respiratory depression
	Johnson et al., 2017 [99] Retrospective cohort observational study	2–17 years n = 75 18 obesity (BMI ≥ P95) 57 non-obesity (BMI < P95) Critically ill (PICU); requiring sedation	Initial infusion 1.0 (1.0–2.0) mcg/kg/h. Final dose 1.5 (1.5–2.4) mcg/kg/h. Sedation assessed using State Behavioral Scale	Median time to target sedation: 10.9 h. In non-obese, higher initial doses reduced probability of achieving target sedation (−19% per +10 mcg/h). No association in obesity	High interindividual variability. TBW-based dosing does not predict sedation response in obesity. Risk of oversedation due to accumulation; dose individualization required
	Vaughns et al., 2017 [100] Pharmacokinetic intervention study	>12 years. n = 6 All severe obesity (BMI ≥ P99) Undergoing bariatric surgery	IV fentanyl dosed by IBW (1–2 mcg/kg). Serial blood sampling up to 24 h	Vd: 4.7 ± 2.1 L/kg (TBW-based) CL: 1522 ± 310 mL/min (11.2 ± 2.6 mL/min/kg). t½: 11.2 ± 6.6 h. AUC: 1.5 ± 0.5 h·ng/mL	Loading dose may be based on TBW; maintenance dosing should use IBW or LBW to avoid accumulation and toxicity
	PopPK/PBPK modeling studies				
	Maharaj et al., 2020 [101] Prospective cohort. PopPK modeling	2–18 years n = 62; 30 obesity (BMI ≥ P95), 32 normal weight Undergoing analgesia/sedation	Population PK model developed exploring weight-based dosing strategies Fentanyl administered as bolus (mean 1 mcg/kg) and continuous infusion (mean 1.4 mcg/kg/h).	V1: 10.8 L; V2: 417 L Cl: 32.5 L/h (70 kg standardized); Q: 104 L/h. Therapeutic range: 1–3 ng/mL. Weight–CL relationship followed allometric scaling	TBW-based dosing increases risk of supratherapeutic exposure with increasing weight. Proposed model-based dosing: infusion rate (mcg/kg/h) = 2.283/(TBW^0.25)
	Lim et al., 2019 [102] Modeling study (PopPK simulation)	0 years–adolescents (simulated NHANES-based population); n = 4376 807 obesity (BMI ≥ P95 or Weight-for-length ≥ P97.7 < 2 years) 3569 non-obesity (BMI < P95) Receiving analgesia/sedation	Simulated population based on NHANES data. Continuous infusion 1 mcg/kg/h (TBW-based). Stratified by age and obesity status	Vss higher in obesity (e.g., 6–12 years: 17.2 vs. 7.2 L). CL absolute slightly higher >6 years, but weight-normalized CL 11–30% lower t½ 2–4× longer Css 25–77% higher in obesity	TBW-based dosing leads to drug accumulation and overexposure. Alternative descriptors (e.g., lean body mass or allometric scaling) should be considered
Fentanyl (and Methadone	Gerhart et al., 2022 [103] Modeling study PBPK	2–18 years Simulated population 52 obesity (BMI ≥ P95) 35 non-obesity (BMI < P95) PBPK model; analgesia/sedation context	PBPK model using pediatric data; simulated fentanyl infusions (1–3 mcg/kg/h) and methadone dosing (0.2 mg/kg q8h)	Fentanyl: Vss increased with weight; weight-normalized CL decreased; TBW-based dosing produced higher Css (target 1–3 ng/mL) Methadone: Slight increase in exposure with obesity; marked increase in exposure in CYP2B6*6/*6 genotype; target Css, min ≥30 ng/mL	Fentanyl: Dose reduction required in obesity. Suggested: 2–<6 y: 2 mcg/kg/h; 6–<12 y: 2 (non-obese) vs. 1 mcg/kg/h (obese); 12–18 y: 1 mcg/kg/h Methadone: Genotype-driven dosing. Standard 0.2 mg/kg q8h; reduces to 0.1 mg/kg in CYP2B6*6/*6, especially in obesity
Morphine	Dalesio et al., 2020 [104] Interventional pharmacokinetic study	Children; (BMI ≥ P95) with OSA vs. non-obese controls (BMI < P95), Undergoing surgery; obesity	Single dose morphine 0.05 mg/kg based on IBW. Serial blood sampling up to 9 h. Quantification of morphine, M3G, M6G (HPLC-)	Vd lower in obesity+OSA (0.09 vs. 0.18 mL/kg). Cmax higher in obesity+OSA (47.6 vs. 30.1 ng/mL). Similar CL between groups (~0.02 mL/min/kg). Increased M3G/morphine ratio and higher M3G Cmax. No significant differences in half-life	IBW-based dosing does not prevent higher exposure in obesity+OSA. Recommend avoiding TBW-based dosing, consider dose reduction and increased dosing frequency, and use multimodal analgesia to reduce respiratory risk
Sedative: fentanyl and morphine	Ward et al., 2025 [105] Secondary analysis of a multicenter randomized clinical trial	Patients (1–17 years) n: 1183 BMI ≥ P95 Obesity 22% (n = 265) Patients were managed either with protocolized sedation or usual care	The protocol indicated the use of TBW, although it suggested considering AjBW if the patient exceeded 130% of their IBW Fentanyl and morphine doses, as well as clinical outcomes, were analyzed	Drug Exposure Fentanyl (obese vs. non-obese): Total daily: 791 vs. 544 μg (*p* < 0.001) Cumulative: 7553 vs. 5496 μg (*p* < 0.001) Morphine (obese vs. non-obese): Total daily: 26.8 vs. 19.4 mg (*p* = 0.02) Cumulative: 417.6 vs. 238.1 mg (*p* = 0.01) Sedative response Inadequate sedation higher in obesity: 37% vs. 24% (*p* < 0.001) Longer mechanical ventilation: 8.5 vs. 6.4 days (*p* < 0.001) Longer PICU stay: 13.7 vs. 10.1 days (*p* = 0.01) Higher mortality (28-day): 10% vs. 6% (*p* = 0.01)	TBW-based dosing leads to higher total drug exposure in obese children; alternative approaches IBW-based initial dosing should be considered

AUC: area under the plasma concentration-time curve; AjBW: adjusted body weight; Cmax: maximum plasma concentration; BMI: body mass index; Cl: total body clearance; Css: steady-state concentration FFM: fat free mass; HPLC: high-performance liquid chromatography; IBW: ideal body weight; IV: intravenous administration; LBW: lean body weight; M3G: morphine 3-glucuronide; M6G: morphine 6-glucuronide; NAFLD: non-alcoholic fatty liver disease; NFM: normal fat mass; NHANES: national health and nutrition examination survey: OSA: obstructive sleep apnea; P: percentile; PBPK: physiologically based pharmacokinetic model PD: pharmacodynamic; PICU: pediatric intensive care unit; PK: pharmacokinetics; PopPK: population pharmacokinetic; OSA: obstructive sleep apnea; TBW: total body weight; Vd: volume of distribution.

**Table 11:** Summary of studies describing the dosing and PK and/or PD aspects of benzodiazepines, hypnotics and sedatives, immunosuppressants, and ions.

	Study/Design	Patients/Sample Size	Methods	Results	Dosing Conclusions
Midazolam	PopPK/PBPK modeling studies				
	Van Rongen et al., 2015 [106] Prospective cohort observational	Patients aged 12–18 years overweight (BMI P85–P95) or obesity (BMI ≥ P95) 19 patients: 3 overweight/16 obese Undergoing orthopedic surgery, tonsillectomy, bariatric surgery	Population PK model with NONMEM software: 7 covariate analysis were used. 3 mg midazolam bolus (3 patients) or 2 mg midazolam bolus (16 patients)	TBW had no influence on Cl (0.66 L/min), but did influence peripheral Vd, which increased in obese patients (154 L) The increase in peripheral Vd may result in a longer t_1/2_, delaying the time to reach steady state in continuous infusion	Doses based on TBW may increase the risk of adverse effects. Dose adjustment should take IBW into account
	Gade et al., 2020 [107] Prospective, experimental controlled trial	Patients > 12 years 36 obese (BMI SDS > 1.282) 30 non-obese (BMI SDS < 1.282) Status epilepticus	Population PK model with NONMEM software Midazolam microdose: 1 µg IV Simulations (status epilepticus): Continuous IV infusion (0.2 mg/kg repeated dose + 0.1 mg/kg/h infusion) Fixed oral dose (7.5 mg at age 9, 10 mg at age 15)	The two-compartment model best describes the PK of midazolam. BMI SDS Significantly affects peripheral Vd and intercompartmental Cl. Does not affect systemic Cl After IV infusion: the higher Vd observed in obese patients delayed reaching steady state; therefore, it increased the risk of accumulation and elevated concentrations with prolonged infusions Fixed buccal dose: in obese patients, concentrations were lower and of shorter duration. Risk of subtherapeutic concentrations	Dosage by total weight or by age can lead to overdoses after prolonged IV administration or underdoses after fixed oral doses
	McCann et al. 2025, [108] PopPK with PBPK modeling	Patients 2–21 years Children: 93 Samples: 164 BMI ≥ P95 Administered per standard of care	PK: NONMEM Two-compartment model Allometric scaling: Cl: exponent 0.75; Vd: exponent 1 Covariates tested: BMI, BMI percentile, obesity status, labs, age, sex, CYP3A4 modulators PBPK Included physiological differences (Increased liver/kidney size ~20%, decreased GFR ~15%) IV bolus and/or infusion	PopPK parameters Cl: 14.9 L/h/70 kg V1: 6.09 L/70 kg V2: 34.5 L/70 kg Interindividual variability (Cl): 185.4% PBPK simulations AUC_0_–∞ increased in obesity: Without obesity: 215.3 ng·h/mL With obesity: 255.8 ng·h/mL Cmax slightly higher in obesity: Differences generally <10% Exposure increase <20% with TBW-based dosing	Dose adjustment may not be necessary for initial dosing Obesity does not appear to significantly affect PK beyond body weight: are best characterized by scaling the TBW
Diazepam	PopPK/PBPK modeling studies				
	McCann et al., 2025 [109] Multicenter observational study using real-world data, with PopPK model	Patients 2–21 years Children: 61 Samples: 169 BMI ≥ P95 Moderate obesity: 100–<120% Severe obesity: ≥120%	External evaluation of an existing PopPK model Reference dosing 0.2 mg/kg IV bolus capped at 8 mg; optional second dose: 0.1 mg/kg (capped at 4 mg) Target: 200–600 ng/mL at 10 min post-dose	PK parameters (combined model) CL: 2.29 L/h/70 kg Vc: 58.2 L/70 kg Vp: 51.8 L/70 kg Interindividual variability CL: 59.8% CV Vc: 214% CV Impact of 0.2 mg/kg capped at 8 mg Children < 6 years: adequate exposure Children ≥ 6 years: plasma concentrations often <200 ng/mL Effect of dosing cap 8 mg cap: leads to significant underdosing in obese children	TBW; dose capping (e.g., 8 mg) leads to underexposure in obese/high-weight children, and higher or uncapped doses may be required to achieve target exposure
Dexmedetomidine	Empirical studies (PK and/or clinical data)				
	Wu et al., 2021 [110] Prospective experimental study	80 patients: 40 obese and 40 non-obese. Ages 3–17 years Obesity: BMI ≥ P95 Control group: BMI P25–P84 Preoperative pediatric sedation in patients scheduled for elective orthopedic surgery	Dexmedetomidine IV bolus over 10 min: 0.3–0.8 µg/kg, in 0.1 µg/kg increments (assigned using a biased coin design) Response: RSS ≥ 4 at 10 min after the end of the infusion ED95 estimation: isotonic regression (PAVA) and bootstrapping	ED95: 0.75 µg/kg (obese; 95% CI 0.638–0.780) vs. 0.74 µg/kg (non-obese; 95% CI 0.598–0.779) Clinical results: adequate sedation from 10 min; peak at approximately 15 min (non-obese) and 20 min (obese); significant RSS–BIS correlation	The ED95 was equivalent in obese and non-obese adolescent children when dexmedetomidine was administered according to TBW
	PopPK/PBPK modeling studies				
	Morse, J.D. et al., 2020 [111] FC Modeling	202 individuals Age: 40.6 weeks postmenstrual–70.8 years No obese pediatric group. Obese adults: Cortínez substudy: 20 obese vs. 20 non-obese. Obesity defined as BMI > 35 kg/m^2^ Administered under anesthesia for elective laparoscopic surgery	Use of NONMEM in pediatric and adult populations, comparing 2-compartment vs. 3-compartment models Identify the optimal Cl and Vd descriptor and generate a model applicable to a wide age/weight range	Three-compartment model with first-order elimination. PK results: V1 25.2 L/70 kg, V2 34.4 L/70 kg, V3 65.4 L/70 kg Cl 0.897 L/min/70 kg. Best described by FFM Vd is best described by NFM, incorporating a fat fraction (FFATV = 0.293)	FFM is suggested for maintenance dosing. Drug distribution may be described using the NFM, with an FfavV value of 0.293
	Morse, J. D. et al., 2021 [112] Narrative review and PK/PD modelling approach	Pediatrics: Body weight (BW) or body mass index (BMI) referenced to percentiles for age (e.g., an 8-year-old child with a BMI of 20 kg/m^2^ = Grade I obesity P95 and a BMI of 30 kg/m^2^ = Grade III obesity (140% of the 95th percentile) Pediatric sedation/anesthesia with dexmedetomidine and its administration via TCI pumps	Narrative review and PK/PD modelling approach to explain how to design/use TCI.	Rapid boluses: An IV bolus of 0.49 µg/kg over 5 s in children (5–9 years) was associated with a drop in heart rate; therefore, slower administration is recommended to avoid excessive early concentrations. Obesity: A 0.75 µg/kg bolus over 10 min in an 8-year-old child showed a higher peak Ce in obesity (2.6 µg/L in normal weight vs. 2.8 and 3.2 µg/L in Grade I and III obesity). Clinical outcomes: Accumulation in deep compartments in obesity, and recovery is slower.	Rapid IV boluses should be avoided; therefore, the loading dose should be administered slowly (10–20 min). When adjusting the loading dose to the desired clinical effect, the Vpe/Ce ratio can be used. In obese pediatric patients, direct dosing based on TBW may result in higher Ce values, with the consequent risk of overexposure.
Cyclosporine	Kasap et al., 2006 [113] Observational Retrospective cohort	0 years–adolescents n = 27; obesity (BMI ≥ P95), overweight (BMI P85–95) Renal transplant recipients	Cyclosporine dosing adjusted by TBW, BMI and BSA; trough (C0) and 2 h post-dose levels (C2); renal function (serum creatinine, GFR)	Lower cyclosporine dose requirements in overweight/obesity across all descriptors (TBW: 3.29 vs. 4.18 mg/kg; BMI: 6.54 vs. 9.56 mg; BSA: 115.35 vs. 136.92 mg/m^2^; *p* < 0.001) Similar C0 and C2 levels despite lower doses Reduced GFR and higher serum creatinine in obesity	Reduced dosage required in overweight/obesity; TBW-based dosing may lead to overexposure
Magnesium sulfate	Vaiyani et al., 2016 [114] Pharmacological intervention study	29 pediatric patients (0 years to adolescents) Number of obese patients not specified. With severe status asthmaticus	Group 1: HDMI: Loading dose: 50 mg/kg of magnesium sulfate over 20 min (up to 2 g max.). Continuous infusion: 40 mg/kg/h for 4 h Total: 210 mg/kg in 4 h 20 min Group 2: sHDMI: No loading dose, continuous infusion: 50 mg/kg/h for 5 h. Total: 250 mg/kg in 5 h. In both regimens, IBW was used for dose calculation in obese patients. Monitored clinical parameters: heart rate, mean arterial pressure, respiratory rate, oxygen saturation, serum magnesium levels, and signs of magnesium toxicity.	Magnesium Levels There were no statistically significant differences in final serum levels between HDMI and sHDMI. Adverse Effects Both regimens were well tolerated hemodynamically. No signs of magnesium toxicity, such as neuromuscular or cardiovascular disturbances, were reported. No arrhythmias, need to discontinue the infusion, or serious adverse effects occurred in any of the patients, including obese patients	IBW in obese patients

AUC: area under the plasma concentration-time curve; BIS: bispectral index; BMI: body mass index; BMI SDS: body mass index standard deviation score; BSA: body surface area; Ce: concentration in the effect Compartment; Cl: total body clearance; Cmax: maximum plasma concentration; ED95: dose producing 95% of the maximal effect; FfatV: factor for fat mass; FFM: fat free mass; GFR: glomerular filtration rate; HDMI: high-dose magnesium infusion; IV: intravenous administration; NFM. Normal fat mass; NONMEM: nonlinear mixed effects modeling; PAVA: the pool-adjacent-violators algorithm PBPK: physiologically based pharmacokinetic model; PK: pharmacokinetics; PopPK: population pharmacokinetic; RSS: Ramsay sedation score; sHDMI: simplified HDMI; TBW: total body weight; TCI: target-controlled infusion; Vd: volume of distribution; Vpe: volume of distribution at the time of peak effect site concentration.

## Data Availability

No new data were created or analyzed in this study. Data sharing is not applicable to this article.

## Supplementary Materials

The following supporting information can be downloaded at: https://www.mdpi.com/article/10.3390/ph19050766/s1. Supplementary File S1: PRISMA 2020 Checklist; Supplementary File S2: The full electronic search strategy for each database; Supplementary File S3: A standard operating procedure.

## Abbreviations

The following abbreviations are used in this manuscript:

ACT	Activated clotting time
AEs	Adverse events
AjBW	Adjusted body weight
ANGI	Angiotensin-Converting Enzyme Inhibitors
Anti-Xa	Anti-activated factor Xa
aPTT	Activated partial thromboplastin time
ARB	Angiotensin II Receptor Blockers
ASA	Classification of physical status of the American Society of Anesthesiologists
AUC	Area under the plasma concentration–time curve
BIS	Bispectral Index
BMI	Body mass index
BMI SDS	Body mass index standard deviation score
BNFc	British National Formulary for Children
BSA	Body surface area
CAE	Childhood absence epilepsy
CBZ	Carbamazepine
CCB	Calcium Channel Blockers
CDC	Centers for Disease Control and Prevention
Cl	Total body clearance
Cl/F	Apparent clearance
Cmax	Maximum plasma concentration
Cmin	Trough concentration
Css	Steady-state concentration
EAIs	Epinephrine auto-injectors
EC50	Concentration producing 50% of the maximum effect
ED95	Dose producing 95% of the maximum effect
Emax	Maximum effect
eGFR	Estimated glomerular filtration rate
EMA	European medicines Agency
F	Bioavailability
FDA	Food and Drug Administration
FfatV	Factor for fat mass
FFM	Fat-free mass
GLP-1	Glucagon-like peptide-1
HAM	High-alert medications
HIT	Heparin-induced thrombocytopenia
HOMA-IR	Homeostasis Model Assessment of Insulin Resistance
HPLC	High-performance liquid chromatography
IBW	Ideal body weight
IDSA	Infectious Diseases Society of America
IM	Intramuscular administration
INR	International Normalized Ratio
IV	Intravenous administration
JBI	Joanna Briggs institute
Ka	Absorption rate constant
LBW	Lean body weight
LDL	Low-Density Lipoprotein
LMWH	Low-molecular-weight heparin
M3G	Morphine 3-glucuronide
M6G	Morphine 3-glucuronide
MATE1	Multidrug And Toxin Extrusion Protein 1
n	Sample size
NAFLD	Non-alcoholic fatty liver disease
NFM	Normal fat mass
NHANES	National Health and Nutrition Examination Survey
NONMEM	Nonlinear Mixed Effects Modeling
OCT1	Organic Cation Transporter 1
OSA	Obstructive sleep apnea
P	Percentile
PAVA	The pool-adjacent-violators algorithm
PBPK	Physiologically based pharmacokinetic model
PD	Pharmacodynamics
PE	Pulmonary embolism
PFH	Phenytoin
PhE	Phenytoin equivalent
PICU	Pediatric Intensive Care Unit
PK	Pharmacokinetics
PopPK	Population pharmacokinetics
PRIS	Propofol infusion syndrome
PWS	Prader–Willi syndrome
RSS	Ramsay Sedation Score
SANRA	Scale for the assessment of narrative review articles assessment of narrative review articles
SC	Subcutaneous administration
SCR	Serum creatinine
SLC22A1	Solute carrier family 22 member 1 gene
t½	Elimination half-life
TBW	Total body weight
TCI	Target-controlled infusion
TDM	Therapeutic drug monitoring
TIVA	Total intravenous anesthesia
Tmax	Time to reach maximum plasma concentration
T1DM	Type 1 diabetes mellitus
T2DM	Type 2 diabetes mellitus
UFH	Unfractionated heparin
Vd	Volume of distribution
Vd/F	Apparent volume of distribution
VPA	Valproic acid
Vpe	Volume of distribution at peak effect site concentration
VTE	Venous thromboembolism
