# Supplementary material for: Dosing Strategies for High-Alert Medications in Obese Pediatric Patients: A Systematic Review
Source: Pharmaceuticals (Basel). 2026 May 13;19(5):766. doi: 10.3390/ph19050766 (PMC13209847; doi:10.3390/ph19050766)
Supplement: Supplementary file 1 [file pharmaceuticals-19-00766-s001.zip › Supplementary File S1 - PRISMA_checklist_completed.pdf]

PRISMA 2020 Checklist – Completed

Item	Checklist item	Location in manuscript
1 Title	Identify the report as a systematic review.	Page 1
2 Abstract	Structured summary including background, objectives, methods, results, conclusions.	Page 1
3 Rationale	Describe the rationale for the review.	Pages 1–3
4 Objectives	Explicit statement of objectives.	Page 3
5 Eligibility criteria	Inclusion/exclusion criteria.	Page 4
6 Information sources	Databases and dates of search.	Pages 4
7 Search strategy	Full search strategy.	Page 4; Supplementary Material
8 Selection process	Screening methods and reviewers.	Page 4
9 Data collection	Data extraction process.	Page 4-5
10a Outcomes	Definition of outcomes.	Page 4-5
10b Other variables	Other variables collected.	Page 4-5
11 Risk of bias	Methods for risk of bias.	Pages 5
12 Effect measures	Effect measures used.	Not applicable (narrative synthesis)
13a-f Synthesis methods	Methods of synthesis.	Page 4
14 Reporting bias	Assessment of reporting bias.	Page 5
15 Certainty of evidence	Assessment of certainty.	Not performed (see Methods justification)

16a Study selection	Flow of studies.	Page 5-6; Figure 1
16b Excluded studies	Reasons for exclusion.	Page 4
17 Study characteristics	Characteristics of included studies.	Pages 4–5; Tables
18 Risk of bias results	Results of bias assessment.	To be added
19 Results of studies	Results per study.	Tables and text
20a-d Synthesis results	Summary of synthesis.	Text
21 Reporting bias results	Results of reporting bias.	Not assessed
22 Certainty of evidence	Certainty results.	Not assessed
23 Discussion	Interpretation and implications.	Discussion section
24a Registration	PROSPERO registration.	Page 3
24b Protocol	Protocol access.	Page 3
24c Amendments	Protocol deviations.	Page 3
25 Funding	Sources of support.	Page 60
26 Competing interests	Conflicts of interest.	Page 61
27 Data availability	Data sharing.	Page 60
