# Supplementary material for: Dosing Strategies for High-Alert Medications in Obese Pediatric Patients: A Systematic Review
Source: Pharmaceuticals (Basel). 2026 May 13;19(5):766. doi: 10.3390/ph19050766 (PMC13209847; doi:10.3390/ph19050766)
Supplement: Supplementary file 1 [file pharmaceuticals-19-00766-s001.zip › Supplementary File S2 - Supplementary Search Strategy.pdf]

Dosing recommendations for high-alert medications in obese pediatric patients

Literature search strategies

Supplementary material — Search appendix

Date of execution: July 2024

Databases searched: MEDLINE (Ovid SP) · EMBASE (Ovid SP) · Cochrane CENTRAL · Web of Science

APPENDIX — MEDLINE (Ovid SP)

Coverage: 1966 to present

Date of execution: 05/07/2024

Search strategy executed in MEDLINE through the Ovid SP platform.The 158 search lines are presented without intermediate result counts, organized into three conceptual blocks. The combination and limit lines are shown highlighted in blue.

Filters: 1990–present | Clinical Queries: reviews/therapy (maximizes sensitivity) | Final line: 158

Line	Search terms
**BLOCK 1 — Pediatric population with obesity (lines 1-10)**	
1	exp Obesity/
2	(morbid obesities or obesities, morbid or obesities, severe or obesity, morbid or obesity, severe or severe obesities or severe obesity).mp.
3	(obesit* or obese or overweight).mp. [mp=title, book title, abstract, original title, name of substance word, subject heading word, floating sub-heading word, keyword heading word, organism supplementary concept word, protocol supplementary concept word, rare disease supplementary concept word, unique identifier, synonyms, population supplementary concept word, anatomy supplementary concept word]
**4**	**1 or 2 or 3**
5	p?ediatric*.mp. or exp Pediatrics/ or (kid or kids).tw. or minor*.tw. or (child* or schoolchild*).tw. or adolescen*.tw. or juvenil*.tw. or youth*.tw. or (teen* or preteen*).tw. or (underage* or under age*).tw. or pubescen*.tw. or p?ediatric*.tw. or boyhood.tw. or girl*.tw.
**6**	**4 and 5**
7	p?ediatric obesit*.mp. or exp Pediatric Obesity/
8	limit 4 to "all child (0 to 18 years)"
9	(adolescent obesity or adolescent overweight or child obesity or childhood obesity or childhood onset obesity or childhood overweight or infant obesity or infant overweight or infantile obesity or obesity, adolescent or obesity, child or obesity, childhood or obesity, childhood onset or obesity, infant or obesity, infantile or obesity, pediatric or obesity in adolescence or obesity in childhood or overweight, adolescent or overweight, childhood or overweight, infant or pediatric obesity).mp.

Line	Search terms
**10**	**6 or 7 or 8 or 9**
**BLOCK 2A — Pharmacokinetics and dosing (lines 11–19)**	
11	exp Pharmacokinetics/ or "Pharmaceutical Preparations"/ or pharmacokinetics.mp. or pharmaceutical prepartation*.mp. [mp=title, book title, abstract, original title, name of substance word, subject heading word, floating sub-heading word, keyword heading word, organism supplementary concept word, protocol supplementary concept word, rare disease supplementary concept word, unique identifier, synonyms, population supplementary concept word, anatomy supplementary concept word]
12	dos*.mp. [mp=title, book title, abstract, original title, name of substance word, subject heading word, floating sub-heading word, keyword heading word, organism supplementary concept word, protocol supplementary concept word, rare disease supplementary concept word, unique identifier, synonyms, population supplementary concept word, anatomy supplementary concept word]
13	((dose or dosing or dosage) adj10 (calculat* or effect* or response or adjust*)).mp. [mp=title, book title, abstract, original title, name of substance word, subject heading word, floating sub-heading word, keyword heading word, organism supplementary concept word, protocol supplementary concept word, rare disease supplementary concept word, unique identifier, synonyms, population supplementary concept word, anatomy supplementary concept word]
**14**	**11 or 12 or 13**
15	"Pharmaceutical Preparations"/
16	exp Drug Dosage Calculations/
**17**	**15 and 16**
**18**	**14 or 17**
**19**	**10 and 18**
**BLOCK 2B — Specific drugs (lines 20–116)**	
20	Glyburide.mp. or exp Glyburide/
21	Glimepiride.mp.
22	Liraglutide.mp. or exp Liraglutide/
23	Metformin.mp. or exp Metformin/
24	Semaglutide.mp.
25	Acenocoumarol.mp. or exp Acenocoumarol/
26	Dabigatran.mp. or exp Dabigatran/
27	Rivaroxaban.mp. or exp Rivaroxaban/
28	Warfarin.mp. or exp Warfarin/
29	Alteplase.mp. or exp Tissue Plasminogen Activator/
30	Argatroban.mp.
31	Fondaparinux.mp. or exp Fondaparinux/
32	Bivalirudin.mp.
33	Heparin.mp. or exp Heparin/
34	Urokinase.mp. or exp Urokinase-Type Plasminogen Activator/
35	Epinephrine.mp. or exp Epinephrine/
36	adrenaline.mp. or exp Epinephrine/

Line	Search terms
37	Digoxin.mp. or exp Digoxin/
38	Phenylephrine.mp. or exp Phenylephrine/
39	Isoproterenol.mp. or exp Isoproterenol/
40	Milrinone.mp. or exp Milrinone/
41	Norepinephrine.mp. or exp Norepinephrine/
42	exp Adenosine/ or Adenosine.mp.
43	Amiodarone.mp. or exp Amiodarone/
44	Atenolol.mp. or exp Atenolol/
45	Esmolol.mp.
46	Flecainide.mp. or exp Flecainide/
47	exp Lidocaine/ or Lidocaine.mp.
48	Procainamide.mp. or exp Procainamide/
49	Propranolol.mp. or exp Propranolol/
50	Etomidate.mp. or exp Etomidate/
51	Ketamine.mp. or exp Ketamine/
52	thiopental.mp. or exp Thiopental/
53	propofol.mp. or exp Propofol/
54	acetaminophen.mp. or exp Acetaminophen/
55	codeine.mp. or exp Codeine/
56	exp Fentanyl/ or fenatanyl.mp.
57	methadone.mp. or exp Methadone/
58	morphine.mp. or exp Morphine/
59	oxycodone.mp. or exp Oxycodone/
60	meperidine.mp. or exp Meperidine/
61	remifentanil.mp. or exp Remifentanil/
62	tramadol.mp. or exp Tramadol/
63	valproate.mp. or exp Valproic Acid/
64	phenytoin.mp. or exp Phenytoin/
65	phenobarbital.mp. or exp Phenobarbital/
66	clobazam.mp. or exp Clobazam/
67	clonazepam.mp. or exp Clonazepam/
68	clorazepate.mp. or exp Clorazepate Dipotassium/
69	exp Diazepam/ or diazepam.mp.
70	lorazepam.mp. or exp Lorazepam/
71	midazolam.mp. or exp Midazolam/
72	exp Amlodipine/ or amlodipine.mp.
73	captopril.mp. or exp Captopril/
74	clonidine.mp. or exp Clonidine/
75	hydralazine.mp. or exp Hydralazine/
76	labetalol.mp. or exp Labetalol/

Line	Search terms
77	methyldopa.mp. or exp Methyldopa/
78	nifedipine.mp. or exp Nifedipine/
79	Nitroprusside.mp. or exp Nitroprusside/
80	verapamil.mp. or exp Verapamil/
81	furosemide.mp. or exp Furosemide/
82	desmopressin.mp. or exp Deamino Arginine Vasopressin/
83	amikacin.mp. or exp Amikacin/
84	exp Gentamicins/ or gentamicin*.mp.
85	tobramycin.mp. or exp Tobramycin/
86	vancomycin.mp. or exp Vancomycin/
87	acyclovir.mp. or exp Acyclovir/
88	ganciclovir.mp. or exp Ganciclovir/
89	mycophenolate mofetil.mp. or exp Mycophenolic Acid/
90	cyclosporine.mp. or exp Cyclosporine/
91	methotrexate.mp. or exp Methotrexate/
92	sirolimus.mp. or exp Sirolimus/
93	tacrolimus.mp. or exp Tacrolimus/
94	atracurium.mp. or exp Atracurium/
95	cisatracurium.mp.
96	rocuronium.mp. or exp Rocuronium/
97	succinylcholine.mp. or exp Succinylcholine/
98	suxamethonium.mp. or exp Succinylcholine/
99	vecuronium.mp. or exp Vecuronium Bromide/
100	dexmedetomidine.mp. or exp Dexmedetomidine/
101	chloral hydrate.mp. or exp Chloral Hydrate/
102	zolpidem.mp. or exp Zolpidem/
103	chlorpromazine.mp. or exp Chlorpromazine/
104	haloperidol.mp. or exp Haloperidol/
105	risperidone.mp. or exp Risperidone/
106	diatrizoate meglumine.mp. or exp Diatrizoate Meglumine/
107	lopromide.mp.
108	calcium gluconate.mp. or exp Calcium Gluconate/
109	sodium chloride.mp. or exp Sodium Chloride/
110	potassium chloride.mp. or exp Potassium Chloride/
111	magnesium sulfate.mp. or exp Magnesium Sulfate/
112	Cardioplegic Solutions.mp. or exp Cardioplegic Solutions/
113	hypertonic glucose.mp. or exp Glucose Solution, Hypertonic/
114	parenteral nutrition.mp. or exp Parenteral Nutrition/
115	exp Baclofen/ or intrathecal baclofen.mp.

Line	Search terms
116	((intravenous or IV or subcutaneous or SC) adj1 insulin).mp. [mp=title, book title, abstract, original title, name of substance word, subject heading word, floating sub-heading word, keyword heading word, organism supplementary concept word, protocol supplementary concept word, rare disease supplementary concept word, unique identifier, synonyms, population supplementary concept word, anatomy supplementary concept word]
**BLOCK 2C — Intersection and pharmacological classes /ad [Administration & Dosage] (lines 117–150)**	
**117**	**or/20-116**
118	exp Hypoglycemic Agents/ad [Administration & Dosage]
119	exp Heparin/ad [Administration & Dosage]
120	exp Platelet Aggregation Inhibitors/ad [Administration & Dosage]
121	exp Cardiotonic Agents/ad [Administration & Dosage]
122	exp Glycosides/ad [Administration & Dosage]
123	exp Anti-Arrhythmia Agents/ad [Administration & Dosage]
124	exp Antihypertensive Agents/ad [Administration & Dosage]
125	exp Diuretics/ad [Administration & Dosage]
126	exp Vasopressins/ad [Administration & Dosage]
127	exp Aminoglycosides/ad [Administration & Dosage]
128	exp Aminoglycosides/ad [Administration & Dosage]
129	exp Glycopeptides/ad [Administration & Dosage]
130	exp Antifungal Agents/ad [Administration & Dosage]
131	exp Amphotericin B/ or Liposomal amphotericin.mp.
132	exp Antiviral Agents/ad [Administration & Dosage]
133	exp Immunosuppressive Agents/ad [Administration & Dosage]
134	muscle blockers.mp.
135	exp Mivacurium/
136	exp Pancuronium/
137	exp Neuromuscular Blocking Agents/ad [Administration & Dosage]
138	exp Anesthetics/ad [Administration & Dosage]
139	exp Analgesics, Non-Narcotic/
140	exp Analgesics, Opioid/ad [Administration & Dosage]
141	exp Anticonvulsants/
142	exp Benzodiazepines/ad [Administration & Dosage]
143	exp "Hypnotics and Sedatives"/
144	exp Antipsychotic Agents/ad [Administration & Dosage]
145	exp Contrast Media/ad [Administration & Dosage]
**146**	**or/118-145**
**147**	**117 or 146**
**148**	**19 and 147**
**149**	**limit 148 to yr="1990-Current"**

Line	Search terms
**150**	**limit 149 to ("reviews (maximizes sensitivity)" or "therapy (maximizes sensitivity)")**
**BLOCK 3 — Critical care / ICU (lines 151–158)**	
151	exp Cardiopulmonary Resuscitation/
152	exp Critical Care/
153	(critical care or resuscitation).mp. [mp=title, book title, abstract, original title, name of substance word, subject heading word, floating sub-heading word, keyword heading word, organism supplementary concept word, protocol supplementary concept word, rare disease supplementary concept word, unique identifier, synonyms, population supplementary concept word, anatomy supplementary concept word]
154	intensive care unit*.mp. or exp Intensive Care Units/
**155**	**151 or 152 or 153 or 154**
**156**	**147 or 155**
**157**	**19 and 156**
**158**	**limit 157 to yr="1990-Current"**

Note: The lines highlighted in blue correspond to Boolean combination operations and methodological/temporal limits.

APPENDIX — EMBASE (Ovid SP)

Embase <1974 to 2024 Week 26>

Date of execution: 05/07/2024

Search strategy executed in EMBASE through the Ovid SP platform. The 160 lines are presented without intermediate counts. Line 8 is not operational in EMBASE (records retained). The final filter uses specificity rather than sensitivity, and records already indexed in MEDLINE are excluded (line 159).

Filters: 1990–present | Clinical Queries: reviews/therapy (maximizes specificity) | Exclusion of MEDLINE records | Final line: 160

Line	Search terms
**BLOCK 1 — Pediatric population with obesity (lines 1–10)**	
1	exp Obesity/
2	(morbid obesities or obesities, morbid or obesities, severe or obesity, morbid or obesity, severe or severe obesities or severe obesity).mp.
3	(obesit* or obese or overweight).mp. [mp=title, abstract, heading word, drug trade name, original title, device manufacturer, drug manufacturer, device trade name, keyword heading word, floating subheading word, candidate term word]
**4**	**1 or 2 or 3**
5	p?ediatric*.mp. or exp Pediatrics/ or (kid or kids).tw. or minor*.tw. or (child* or schoolchild*).tw. or adolescen*.tw. or juvenil*.tw. or youth*.tw. or (teen* or preteen*).tw. or (underage* or under age*).tw. or pubescen*.tw. or p?ediatric*.tw. or boyhood.tw. or girl*.tw.
**6**	**4 and 5**
7	p?ediatric obesit*.mp. or exp Pediatric Obesity/
8	limit 4 to "all child (0 to 18 years)" *[Limit not valid in Embase; records were retained]*
9	(adolescent obesity or adolescent overweight or child obesity or childhood obesity or childhood onset obesity or childhood overweight or infant obesity or infant overweight or infantile obesity or obesity, adolescent or obesity, child or obesity, childhood or obesity, childhood onset or obesity, infant or obesity, infantile or obesity, pediatric or obesity in adolescence or obesity in childhood or overweight, adolescent or overweight, childhood or overweight, infant or pediatric obesity).mp.
**10**	**6 or 7 or 8 or 9**
**BLOCK 2A — Pharmacokinetics and dosing (lines 11–19)**	
11	exp Pharmacokinetics/ or "Pharmaceutical Preparations"/ or pharmacokinetics.mp. or pharmaceutical prepartation*.mp. [mp=title, abstract, heading word, drug trade name, original title, device manufacturer, drug manufacturer, device trade name, keyword heading word, floating subheading word, candidate term word]
12	dos*.mp. [mp=title, abstract, heading word, drug trade name, original title, device manufacturer, drug manufacturer, device trade name, keyword heading word, floating subheading word, candidate term word]
13	((dose or dosing or dosage) adj10 (calculat* or effect* or response or adjust*)).mp. [mp=title, abstract, heading word, drug trade name, original title, device manufacturer, drug manufacturer, device trade name, keyword heading word, floating subheading word, candidate term word]

Line	Search terms
**14**	**11 or 12 or 13**
15	"Pharmaceutical Preparations"/
16	exp Drug Dosage Calculations/
**17**	**15 and 16**
**18**	**14 or 17**
**19**	**10 and 18**
**BLOCK 2B — Specific drugs (lines 20–116)**	
20	Glyburide.mp. or exp Glyburide/
21	Glimepiride.mp.
22	Liraglutide.mp. or exp Liraglutide/
23	Metformin.mp. or exp Metformin/
24	Semaglutide.mp.
25	Acenocoumarol.mp. or exp Acenocoumarol/
26	Dabigatran.mp. or exp Dabigatran/
27	Rivaroxaban.mp. or exp Rivaroxaban/
28	Warfarin.mp. or exp Warfarin/
29	Alteplase.mp. or exp Tissue Plasminogen Activator/
30	Argatroban.mp.
31	Fondaparinux.mp. or exp Fondaparinux/
32	Bivalirudin.mp.
33	Heparin.mp. or exp Heparin/
34	Urokinase.mp. or exp Urokinase-Type Plasminogen Activator/
35	Epinephrine.mp. or exp Epinephrine/
36	adrenaline.mp. or exp Epinephrine/
37	Digoxin.mp. or exp Digoxin/
38	Phenylephrine.mp. or exp Phenylephrine/
39	Isoproterenol.mp. or exp Isoproterenol/
40	Milrinone.mp. or exp Milrinone/
41	Norepinephrine.mp. or exp Norepinephrine/
42	exp Adenosine/ or Adenosine.mp.
43	Amiodarone.mp. or exp Amiodarone/
44	Atenolol.mp. or exp Atenolol
45	Esmolol.mp.
46	Flecainide.mp. or exp Flecainide/
47	exp Lidocaine/ or Lidocaine.mp.
48	Procainamide.mp. or exp Procainamide/
49	Propranolol.mp. or exp Propranolol/
50	Etomidate.mp. or exp Etomidate/
51	Ketamine.mp. or exp Ketamine/
52	thiopental.mp. or exp Thiopental/

Line	Search terms
53	propofol.mp. or exp Propofol/
54	acetaminophen.mp. or exp Acetaminophen/
55	codeine.mp. or exp Codeine/
56	exp Fentanyl/ or fenatanyl.mp.
57	methadone.mp. or exp Methadone/
58	morphine.mp. or exp Morphine/
59	oxycodone.mp. or exp Oxycodone/
60	meperidine.mp. or exp Meperidine/
61	remifentanil.mp. or exp Remifentanil/
62	tramadol.mp. or exp Tramadol/
63	valproate.mp. or exp Valproic Acid/
64	phenytoin.mp. or exp Phenytoin/
65	phenobarbital.mp. or exp Phenobarbital/
66	clobazam.mp. or exp Clobazam/
67	clonazepam.mp. or exp Clonazepam/
68	clorazepate.mp. or exp Clorazepate Dipotassium/
69	exp Diazepam/ or diazepam.mp.
70	lorazepam.mp. or exp Lorazepam/
71	midazolam.mp. or exp Midazolam/
72	exp Amlodipine/ or amlodipine.mp.
73	captopril.mp. or exp Captopril/
74	clonidine.mp. or exp Clonidine/
75	hydralazine.mp. or exp Hydralazine/
76	labetalol.mp. or exp Labetalol/
77	methyldopa.mp. or exp Methyldopa/
78	nifedipine.mp. or exp Nifedipine/
79	Nitroprusside.mp. or exp Nitroprusside/
80	verapamil.mp. or exp Verapamil/
81	furosemide.mp. or exp Furosemide/
82	desmopressin.mp. or exp Deamino Arginine Vasopressin/
83	amikacin.mp. or exp Amikacin/
84	exp Gentamicins/ or gentamicin*.mp.
85	tobramycin.mp. or exp Tobramycin/
86	vancomycin.mp. or exp Vancomycin/
87	acyclovir.mp. or exp Acyclovir/
88	ganciclovir.mp. or exp Ganciclovir/
89	mycophenolate mofetil.mp. or exp Mycophenolic Acid/
90	cyclosporine.mp. or exp Cyclosporine/
91	methotrexate.mp. or exp Methotrexate/
92	sirolimus.mp. or exp Sirolimus/

Line	Search terms
93	tacrolimus.mp. or exp Tacrolimus/
94	atracurium.mp. or exp Atracurium/
95	cisatracurium.mp.
96	rocuronium.mp. or exp Rocuronium/
97	succinylcholine.mp. or exp Succinylcholine/
98	suxamethonium.mp. or exp Succinylcholine/
99	vecuronium.mp. or exp Vecuronium Bromide/
100	dexmedetomidine.mp. or exp Dexmedetomidine/
101	chloral hydrate.mp. or exp Chloral Hydrate/
102	zolpidem.mp. or exp Zolpidem/
103	chlorpromazine.mp. or exp Chlorpromazine/
104	haloperidol.mp. or exp Haloperidol/
105	risperidone.mp. or exp Risperidone/
106	diatrizoate meglumine.mp. or exp Diatrizoate Meglumine/
107	lopromide.mp.
108	calcium gluconate.mp. or exp Calcium Gluconate/
109	sodium chloride.mp. or exp Sodium Chloride/
110	potassium chloride.mp. or exp Potassium Chloride/
111	magnesium sulfate.mp. or exp Magnesium Sulfate/
112	Cardioplegic Solutions.mp. or exp Cardioplegic Solutions/
113	hypertonic glucose.mp. or exp Glucose Solution, Hypertonic/
114	parenteral nutrition.mp. or exp Parenteral Nutrition/
115	exp Baclofen/ or intrathecal baclofen.mp.
116	((intravenous or IV or subcutaneous or SC) adj1 insulin).mp. [mp=title, abstract, heading word, drug trade name, original title, device manufacturer, drug manufacturer, device trade name, keyword heading word, floating subheading word, candidate term word]
**BLOCK 2C — Intersection and pharmacological classes /ad [Administration & Dosage] (lines 117-150)**	
**117**	**or/20-116**
118	exp Hypoglycemic Agents/ad [Administration & Dosage]
119	exp Heparin/ad [Administration & Dosage]
120	exp Platelet Aggregation Inhibitors/ad [Administration & Dosage]
121	exp Cardiotonic Agents/ad [Administration & Dosage]
122	exp Glycosides/ad [Administration & Dosage]
123	exp Anti-Arrhythmia Agents/ad [Administration & Dosage]
124	exp Antihypertensive Agents/ad [Administration & Dosage]
125	exp Diuretics/ad [Administration & Dosage]
126	exp Vasopressins/ad [Administration & Dosage]
127	exp Aminoglycosides/ad [Administration & Dosage]
128	exp Aminoglycosides/ad [Administration & Dosage]

Line	Search terms
129	exp Glycopeptides/ad [Administration & Dosage]
130	exp Antifungal Agents/ad [Administration & Dosage]
131	exp Amphotericin B/ or Liposomal amphotericin.mp.
132	exp Antiviral Agents/ad [Administration & Dosage]
133	exp Immunosuppressive Agents/ad [Administration & Dosage]
134	muscle blockers.mp.
135	exp Mivacurium/
136	exp Pancuronium/
137	exp Neuromuscular Blocking Agents/ad [Administration & Dosage]
138	exp Anesthetics/ad [Administration & Dosage]
139	exp Analgesics, Non-Narcotic/
140	exp Analgesics, Opioid/ad [Administration & Dosage]
141	exp Anticonvulsants/
142	exp Benzodiazepines/ad [Administration & Dosage]
143	exp "Hypnotics and Sedatives"/
144	exp Antipsychotic Agents/ad [Administration & Dosage]
145	exp Contrast Media/ad [Administration & Dosage]
**146**	**or/118-145**
**147**	**117 or 146**
**148**	**19 and 147**
**149**	**limit 148 to yr="1990-Current"**
**150**	**limit 149 to ("reviews (maximizes specificity)" or "therapy (maximizes specificity)")**
**BLOCK 3 — Critical care / ICU (lines 151–160)**	
151	exp Cardiopulmonary Resuscitation/
152	exp Critical Care/
153	(critical care or resuscitation).mp. [mp=title, abstract, heading word, drug trade name, original title, device manufacturer, drug manufacturer, device trade name, keyword heading word, floating subheading word, candidate term word]
154	intensive care unit*.mp. or exp Intensive Care Units/
**155**	**151 or 152 or 153 or 154**
**156**	**147 or 155**
**157**	**19 and 156**
**158**	**limit 157 to yr="1990-Current"**
**159**	**limit 158 to "remove medline records"**
**160**	**limit 159 to ("reviews (maximizes specificity)" or "therapy (maximizes specificity)")**

Note: The lines highlighted in blue correspond to Boolean combinations and limits. The italic annotation in line 8 indicates that this limit is not operational in EMBASE.

APPENDIX — Cochrane CENTRAL Register of Controlled Trials

Search Name: CHILDHOOD OBESITY DOSING HIGH-RISK MEDICATIONS

Date of execution: 05/07/2024

Search strategy executed in Cochrane CENTRAL on July 5, 2024. Structured in three blocks: MeSH descriptors for population and dose calculation, free-text population with dosing, and intersection with drugs. Limited to records in the CENTRAL repository.

Characteristics: Includes records from MEDLINE, EMBASE, and other sources. No additional time filter. Results limited to CENTRAL. Final line: #21

Line	Search terms
**BLOCK 1 — Pediatric population with obesity (lines #1–#7, MeSH)**	
#1	MeSH descriptor: [Obesity] explode all trees
#2	MeSH descriptor: [Pediatrics] explode all trees
**#3**	**#1 and #2**
#4	MeSH descriptor: [Pediatric Obesity] explode all trees
**#5**	**#3 OR #4**
#6	MeSH descriptor: [Drug Dosage Calculations] explode all trees
**#7**	**#5 AND #6**
**BLOCK 2 — Free-text search: population and dosing (lines #8–#17)**	
#8	morbid obesities or obesities, morbid or obesities, severe or obesity, morbid or obesity, severe or severe obesities or severe obesity OR obesit* or obese or overweigh*
#9	pediatric* or paediatric or kid* or minors or child* or schoolchild* or adolescen*
#10	juvenil* or youth* or teen* or preteen* or underage* or under age* or pubescen* or boyhood or girl*
**#11**	**#9 or #10**
**#12**	**#8 AND #11**
#13	(pediatric obesity or adolescent obesity or adolescent overweight or child obesity or childhood obesity or childhood onset obesity or childhood overweight or infant obesity or infant overweight or infantile obesity or obesity, adolescent or obesity, child or obesity, childhood or obesity, childhood onset or obesity, infant or obesity, infantile or obesity, pediatric or obesity in adolescence or obesity in childhood or overweight, adolescent or overweight, childhood or overweight, infant or pediatric obesity)
**#14**	**#12 OR #13**
#15	DOS*
**#16**	**#14 AND #15**
**#17**	**#7 OR #16**
**BLOCK 3 — Drugs: classes and active substances (lines #18–#21)**	
#18	Hypoglycemic Agents or Heparin or Platelet Aggregation Inhibitors or Cardiotonic Agents or glycosides or anti-Arrhythmia Agents or Antihypertensive Agents or diuretics or Vasopressins or Aminoglycosides or Glycopeptides or Antifungal Agents or Amphotericin B or Liposomal amphotericin or Antiviral Agents or Immunosuppressive or muscle blockers or Mivacurium or Pancuronium or Neuromuscular Blocking Agents or

Line	Search terms
	Anesthetic or Analgesics, Non-Narcotic or Analgesics, Opioid or anticonvulsants or Benzodiazepines or Hypnotics or Sedatives or Antipsychotic Agents or Contrast Media
#19	Glyburide OR Glimepiride OR Liraglutide OR Metformin OR Semaglutide OR Acenocoumarol OR Dabigatran OR Rivaroxaban OR Warfarin OR Alteplase OR Tissue Plasminogen Activator OR Argatroban OR Fondaparinux OR Bivalirudin OR Heparin OR Urokinase OR Epinephrine OR adrenaline OR Digoxin OR Phenylephrine OR Isoproterenol OR Milrinone OR Norepinephrine OR Adenosine OR Amiodarone OR Atenolol OR Esmolol OR Flecainide OR Lidocaine OR Procainamide OR Propranolol OR Etomidate OR Ketamine OR Thiopental OR Propofol OR Acetaminophen OR Codeine OR Fentanyl OR Methadone OR Morphine OR Oxycodone OR Meperidine OR Remifentanil OR Tramadol OR Valproate OR valproic acid OR Phenytoin OR Phenobarbital OR Clobazam OR Clonazepam OR Clorazepate OR Diazepam OR Lorazepam OR Midazolam OR Amlodipine OR Captopril OR Clonidine OR Hydralazine OR Labetalol OR Methyldopa OR Nifedipine OR Nitroprusside OR Verapamil OR Furosemide OR Desmopressin OR Deamino Arginine Vasopressin OR Amikacin OR Gentamicin OR Tobramycin OR Vancomycin OR Acyclovir OR Ganciclovir OR Mycophenolate mofetil OR Mycophenolic Acid OR Cyclosporine OR Methotrexate OR Sirolimus OR Tacrolimus OR Atracurium OR Cisatracurium OR Rocuronium OR Succinylcholine OR suxamethonium OR Vecuronium OR Dexmedetomidine OR Chloral hydrate OR Zolpidem OR Chlorpromazine OR Haloperidol OR Risperidone OR Diatrizoate meglumine OR Iopromide OR Calcium gluconate OR Sodium chloride OR Potassium chloride OR Magnesium sulfate OR Cardioplegic solutions OR hypertonic glucose OR Parenteral nutrition OR intrathecal baclofen OR baclofen
**#20**	**#18 or #19**
**#21**	**#17 AND #20 [LIMITADO A CENTRAL]**

Note: The lines highlighted in blue correspond to Boolean combination operations (OR/AND). Line #21 constitutes the final result limited to CENTRAL.

APPENDIX — Web of Science (WOS) — Core Collection

WOS.SCI (1900–2024) · WOS.SSCI (1956–2024) · WOS.IC (1993–2024) and others

Date of execution: 05/07/2024

Search strategy executed in the Web of Science Core Collection on July 5, 2024. It uses the operators TS= (topic: title, abstract, keywords, and Keywords Plus) and ALL= (all fields). Structured into two blocks: pediatric population with obesity and dosing, and drugs. Time limit 1990–2024 in line 12.

Filters: PY=1990–2024 | No document type filter | Final line: 13

Line	Search terms
**BLOCK 1 — Pediatric population with obesity (lines 1-7)**	
1	TS=((morbid obesities or obesities, morbid or obesities, severe or obesity, morbid or obesity, severe or severe obesities or severe obesity OR obesit* or obese or overweight))
2	ALL=(p?ediatric* or (kid or kids) or minors or (child* or schoolchild*) or adolescen* or juvenil* or youth* or (teen* or preteen*) or (underage* or under age*) or pubescen* or p?ediatric* or boyhood or girl*)
**3**	**#2 AND #1**
4	ALL=(p?ediatric obesity or adolescent obesity or adolescent overweight or child obesity or childhood obesity or childhood onset obesity or childhood overweight or infant obesity or infant overweight or infantile obesity or obesity, adolescent or obesity, child or obesity, childhood or obesity, childhood onset or obesity, infant or obesity, infantile or obesity, pediatric or obesity in adolescence or obesity in childhood or overweight, adolescent or overweight, childhood or overweight, infant or pediatric obesity)
**5**	**#4 OR #3**
6	ALL=((Pharmacokinetic* OR Pharmaceutical Preparation* OR dos*))
**7**	**#6 AND #5**
**BLOCK 2 — Drugs and final intersection (lines 8-13)**	
8	TS=(Hypoglycemic Agents or Heparin or Platelet Aggregation Inhibitors or Cardiotonic Agents or glycosides or anti-Arrhythmia Agents or Antihypertensive Agents or diuretics or Vasopressins or Aminoglycosides or Glycopeptides or Antifungal Agents or Amphotericin B or Liposomal amphotericin or Antiviral Agents or Immunosuppressive or muscle blockers or Mivacurium or Pancuronium or Neuromuscular Blocking Agents or Anesthetic or Analgesics, Non-Narcotic or Analgesics, Opioid or anticonvulsants or Benzodiazepines or "Hypnotics and Sedatives" or Antipsychotic Agents or Contrast Media)
9	TS=(Glyburide OR Glimepiride OR Liraglutide OR Metformin OR Semaglutide OR Acenocoumarol OR Dabigatran OR Rivaroxaban OR Warfarin OR Alteplase OR Tissue Plasminogen Activator OR Argatroban OR Fondaparinux OR Bivalirudin OR Heparin OR Urokinase OR Epinephrine OR adrenaline OR Digoxin OR Phenylephrine OR Isoproterenol OR Milrinone OR Norepinephrine OR Adenosine OR Amiodarone OR Atenolol OR Esmolol OR Flecainide OR Lidocaine OR Procainamide OR Propranolol OR Etomidate OR Ketamine OR Thiopental OR Propofol OR Acetaminophen OR Codeine OR Fentanyl OR Methadone OR Morphine OR Oxycodone OR Meperidine OR Remifentanil OR Tramadol OR Valproate OR valproic acid OR Phenytoin OR Phenobarbital OR Clobazam OR Clonazepam OR Clorazepate OR Diazepam OR Lorazepam OR idazoxan OR Amlodipine OR Captopril OR Clonidine OR Hydralazine OR Labetalol OR Methyldopa OR Nifedipine OR Nitroprusside OR Verapamil OR Furosemide OR Desmopressin OR Deamino Arginine Vasopressin OR Amikacin OR Gentamicin OR Tobramycin OR Vancomycin OR Acyclovir OR Ganciclovir OR Mycophenolate mofetil OR Mycophenolic Acid OR Cyclosporine OR

Line	Search terms
	Methotrexate OR Sirolimus OR Tacrolimus OR Atracurium OR Cisatracurium OR Rocuronium OR Succinylcholine OR suxamethonium OR Vecuronium OR Dexmedetomidine OR Chloral hydrate OR Zolpidem OR Chlorpromazine OR Haloperidol OR Risperidone OR Diatrizoate meglumine OR Iopromide OR Calcium gluconate OR Sodium chloride OR Potassium chloride OR Magnesium sulfate OR Cardioplegic solutions OR hypertonic glucose OR Parenteral nutrition OR intrathecal baclofen OR baclofen)
**10**	**#9 OR #8**
**11**	**#10 AND #7**
12	PY=(1990-2024)
**13**	**#12 AND #11**

Note: The lines highlighted in blue correspond to Boolean combinations and the time limit. The operators TS= and ALL= are specific to the Web of Science syntax.
