# Supplementary material for: Dosing Strategies for High-Alert Medications in Obese Pediatric Patients: A Systematic Review
Source: Pharmaceuticals (Basel). 2026 May 13;19(5):766. doi: 10.3390/ph19050766 (PMC13209847; doi:10.3390/ph19050766)
Supplement: Supplementary file 1 [file pharmaceuticals-19-00766-s001.zip › Supplementary File S3 - SOP traslation.pdf]

Standard Operating Procedure (SOP) for the Article Screening and Selection Process in Rayyan

Version 1.0

Prepared: September 2024

Project: Dosing recommendations for high-risk medications in obese pediatric patients.

- Objective: to provide an SOP for the evaluation of articles on the RAYYAN platform during the systematic review process of this research proposal.
- Applicability: researchers involved in the review project.
- Software or materials required for the procedure: registered user accounts on the Rayyan platform.
- **Preliminary procedure:**
  - **Systematic review framework:**
    - P: pediatric patients with obesity and/or overweight.
    - I: effects of obesity on the disposition of high-risk medications (HRM) and on dosing.
    - C: non-obese pediatric patients.
    - O: changes in pharmacokinetic (PK) parameters and dosing recommendations in obese pediatric patients.
  - **Databases used:**
    - Medline.
    - Embase.
    - Woss.
    - Cochrane-Central.
  - Language: English and Spanish.

- **Screening or evaluation process of the articles**

The selection of articles will be performed in pairs, and in cases of discrepancy, the full text of the article will be reviewed by a third person, who will be responsible for deciding whether the study is included.

We will use the Rayyan tool (the attached file explains the possibilities it offers; however, we will hold an online meeting to explain its operation and the working methodology).

Initially, we will carry out a comprehensive reading of the title and abstract (sometimes, the title alone can provide a fairly accurate idea of the article).

We will also have all the information related to the article: type of publication, authors, topics, journal, year of publication, keywords, url ...

The options for screening or selection of each article are:

INCLUDE: when the title or abstract matches the PICO question. The inclusion criteria for the articles are detailed below:

- Included patients: obese and/or overweight pediatric patients.
- Studies referring to the HRM included in the study. (list of HRM attached)

"AND"

- Studies that determine PK parameters such as: Vd, Clp and t1/2, area under the curve and/or plasma concentrations in obese and/or overweight pediatric patients.

"OR"

- Studies that include dosing recommendations in obese and/or overweight pediatric patients.
- Study designs to be included:
    - Prospective and/or retrospective studies.
    - Comparative and randomized studies.
    - Non-randomized comparative studies (cohorts and case/control).
    - Without control group: case reports or case series.

MAYBE: when it is unclear whether it should be included or excluded.

In **Notes**, we can include any observations we consider relevant.

EXCLUDE: when the title and abstract do not match the pico question. The exclusion criteria for the articles are detailed below:

- **Excluded patients:** adult patients.
- TAll studies that do NOT focus on the description of PK data or dosing estimations in obese patients.
- All studies that do not include the HAM selected for the project.
- All studies not written in english or spanish.
- **Study types to be excluded:**
    - Editorials.
    - Letters to the editor.
    - Conference abstracts.

When an article is excluded, the reasons must be provided:

The exclusion reasons are already configured in Rayyan and are as follows:

- Background article.
- Foreign language.
- Wrong drug.
- Wrong outcome.
- Wrong population.
- Wrong publication type.
- Wrong study design.
- Wrong study duration.

The selection of the exclusion reason will be carried out according to the algorithm shown in the following figure:

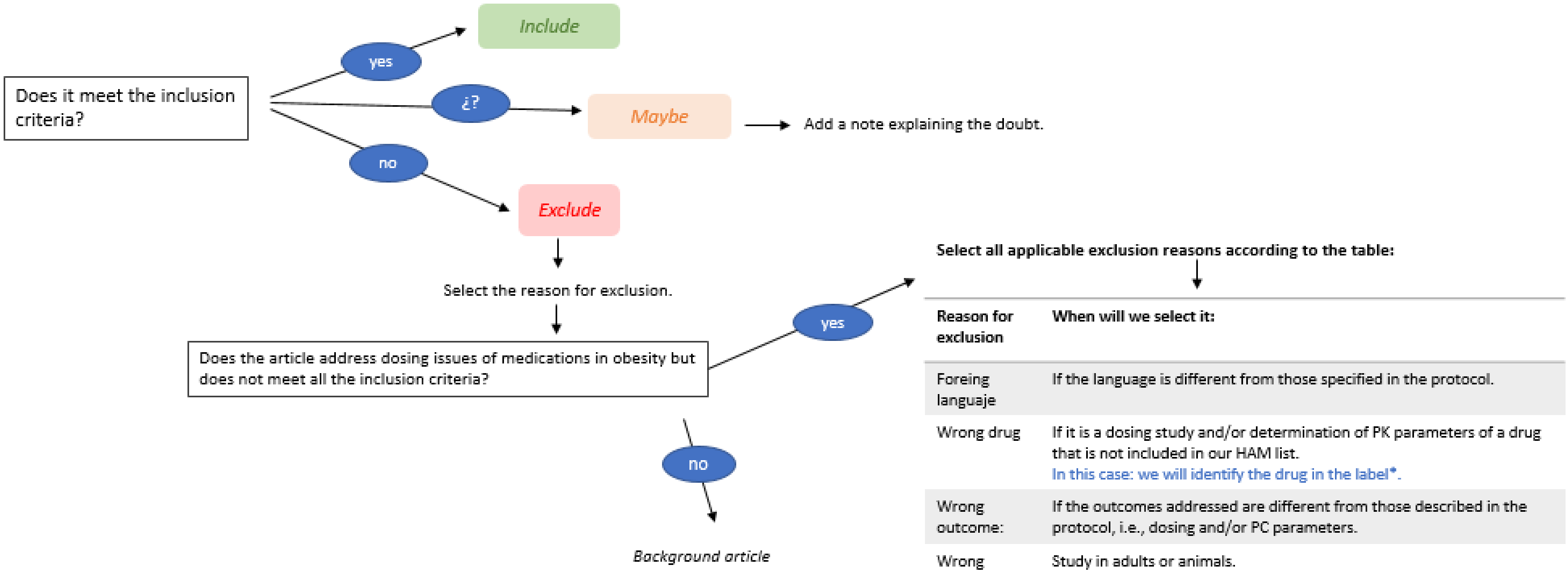

Select all applicable exclusion reasons according to the table:

Reason for exclusion	When will we select it:
Foreing languaje	If the language is different from those specified in the protocol.
Wrong drug	If it is a dosing study and/or determination of PK parameters of a drug that is not included in our HAM list.<br>In this case: we will identify the drug in the label*.
Wrong outcome:	If the outcomes addressed are different from those described in the protocol, i.e., dosing and/or PC parameters.
Wrong population	Study in adults or animals.<br>In this case: we will identify the drug in the label*.
Wrong publication type	If the study corresponds to any of the following publication types:<br>• Editorials.<br>• Letters to the editor.<br>• Conference abstracts.
Wrong study desing:	If it is NOT one of the following study designs:<br>• Experimental: clinical trials (randomized or not, with or without a control group).<br>• Observational: case-control and cohort studies.<br>• Case reports or case series.
Wrong study duration:	If the study duration is inadequate.

*See explanation on how to create a label

FIGURE:
ALGORITHM FOR ARTICLE CLASSIFICATION IN RAYYAN
DOSING OF HRM IN OBESE PEDIATRIC PATIENTS

Labels

We will use them in two situations:

- When it is a dosing study and/or definition of PK parameters in obese pediatric patients for a drug not included in our HAM list.
- When it is a dosing study and/or definition of PK parameters in obese adult patients for HAM.

In the label, we will include the DRUG involved.

Labels are NOT preconfigured; therefore, each time we use them, we will write the name of the drug and then select it.

That label (in this case, the drug name) will then be saved and can be used subsequently.

Notes

We will use them when we classify the article as doubtful ("maybe") to provide clarification for the focus team.

However, regardless of whether we classify the article as included, excluded, or doubtful, we may include any note we consider relevant.
